# Unveiling Patient-Level Harms and At-Risk Clinical Groups During Hospital Cyberattacks: Systematic Review of Global Case Studies and Social Media Data

**DOI:** 10.2196/88257

**Published:** 2026-09-28

**Authors:** Isabel Straw, Deepak Kumar, Seoyoung Kweon, Jordan Selzer, Christian Dameff, Jeffrey Tully

**Affiliations:** 1 Department of Computer Science and Engineering, University of California San Diego, 9500 Gilman Dr, San Diego, CA, 92093, United States, 1 858-534-2230; 2 Department of Emergency Medicine, George Washington University, Washington, DC, United States; 3 Department of Emergency Medicine, University of California San Diego, San Diego, CA, United States; 4 Department of Anesthesia, University of California San Diego, San Diego, CA, United States

**Keywords:** Digital health, medical informatics, cybersecurity, cyberattacks, public health, PRISMA

## Abstract

**Background:**

Cyberattacks against health care organizations are rising in frequency and shifting in nature from data theft to intentional denial of care.

**Objective:**

This systematic review identifies and characterizes patient-level harms occurring during hospital cyberattacks across international settings, determines which clinical populations are most vulnerable, and compares patient-level harms reported in peer-reviewed case studies with those described in patient and provider narratives on social media.

**Methods:**

Following PRISMA (Preferred Reporting Items for Systematic Reviews and Meta-Analyses) 2020, PRISMA-S (Preferred Reporting Items for Systematic Reviews and Meta-Analyses–Search), and the SWiM (Synthesis Without Meta-analysis) reporting guidelines, we searched MEDLINE, Embase, PubMed, Scopus, and Web of Science for peer-reviewed studies published between January 1, 2004, and July 1, 2026 describing cyberattacks affecting clinical care in health care delivery organizations. Quantitative synthesis was not performed because the included studies reported structurally incompatible outcome types for which no common effect measure existed; synthesis without meta-analysis was conducted according to SWiM. In parallel, a Python (Python Software Foundation) pipeline mined 3408 health care–related subreddits for cyberattack terms extracted during the academic review; posts were reviewed by clinical team members to ensure relevance and coded to clinical specialties and technical failure domains.

**Results:**

Fifty-nine studies were included, from which 316 patient-level harms were identified across the combined datasets. Two distinct categories of at-risk patients emerged: those harmed by time-criticality, including patients with stroke, cardiac arrest, and major trauma, for whom delays of minutes to hours are clinically decisive; and those harmed by the intersection of digital dependence and potential for rapid clinical decline, including patients with cancer, patients with diabetes, and those dependent on community prescribing. Specific technical failures were linked to distinct clinical consequences, including those stemming from (1) hardware (oncological harms due to compromised linear accelerators), (2) software (risks for patients with fractures due to failures in digital templating software), and (3) networks/connectivity (fetal deaths due to telemetry failures). Social media data exposed wider harms resulting from infrastructural issues, including compromised door access systems preventing life-saving treatment, failures in ward-based alarms, and closed-circuit television (CCTV) affecting staff/patient safety, and disrupted health-at-home services preventing community therapies.

**Conclusions:**

Our study is the first to systematically integrate global case study evidence with large-scale social media data to provide a clinically focused synthesis of patient-level harms during health care cyberattacks. The resulting open-source Cyberattack Impacts, Patient Harms & Emergency Response (CIPHER) dataset links technical failure domains to downstream clinical risks across care settings. As cyberattacks on health care organizations continue to rise in frequency and severity, the boundary between cybersecurity and patient safety has become increasingly blurred, demanding unified frameworks that treat digital resilience and clinical preparedness as inseparable expressions of the same obligation to patients.

## Introduction

In recent years, the health care sector has experienced a sharp increase in cyberattacks that cause measurable patient harm. For example, ransomware created a national blood shortage that delayed life-saving surgeries, a data breach exposed thousands of patients’ intimate medical images online, and courts settled the first lawsuit linking a cyberattack to an infant death [1-3]. These stories exemplify the harms that occur when cybersecurity and health care collide, where digital exploits translate into compromised care and adverse patient experiences.

The rise in health care cyberattacks has been described extensively in the literature, with Neprash et al [4] revealing that incidents more than doubled between 2016 and 2021. Alongside this increase in frequency, there has been a concomitant shift from cyberattacks focused on data theft for monetary gain to attacks that intentionally disrupt patient care [4,5]. Described by the American Hospital Association as *“*threat-to-life” crimes, hackers have weaponized their ability to disrupt clinical care in order to apply pressure for ransom payments to maximize financial benefits [5]. In addition to financially incentivized attacks, these “threat-to-life” crimes have emerged as a central component of geopolitical conflicts, highlighted by the European Parliament in its reports on the hybrid war in Ukraine [6]. The rising number of health care cyberattacks, paired with the shift toward “denial of care,” has resulted in diverse incidents of cyberattack-induced patient harms occurring in hospitals worldwide [1-7].

News outlets reported the death of a woman in Germany due to cyberattack-induced delays in emergency care, while in the United Kingdom the 2024 Synovis attack was reported to cause 170 incidents of patient harm from canceled transfusions and surgeries and one confirmed patient death [7-9]. Despite the clear patient safety implications of cyber threats, there is limited research translating cyberattack events into downstream clinical effects, limiting our ability to quantify clinical risks, optimize clinical preparedness within hospitals, and effectively safeguard patients [9-16]. Overwhelmingly, the literature on health care cybersecurity reflects computing and technical perspectives, creating a critical knowledge gap in understanding how digital exploits manifest as patient illness and injury [14-17]. Furthermore, reviews of health care cybersecurity published in the medical literature tend to focus on the vulnerabilities present in digital health systems, as opposed to quantifying the clinical effects that emerge when these vulnerabilities are exploited [18-21].

To bridge this translational gap, this research evaluates health care cyberattacks through a clinical lens. This review adopts a global perspective, evaluating case studies of hospital cyberattacks occurring worldwide, to identify transnational themes and draw international comparisons. In doing so, this paper approaches cyberattacks in a similar manner to other evolving public health threats, where international comparative analysis was vital to enriching scientific understanding and informing evidence-based policy making for safeguarding population health (eg, COVID-19) [22].

While the academic literature is an essential source of information for evaluating public health threats, population health researchers have exposed the limitations of peer-reviewed articles for detection of rapidly evolving public health harms [23]. Since the COVID-19 pandemic, we have seen the integration of informal sources of data (eg, social media) into population health models to enrich surveillance programs and improve public health forecasting of rapidly changing threats [23-26]. The value of such data has been particularly highlighted for analyzing cyberattacks, as websites and online chat rooms frequently become venues for discussion of cyberattack effects [27,28]. Furthermore, social media data provide a mechanism for circumventing the existing barriers to reporting patient harms during cyberattacks, such as the silencing of staff, issues relating to confusion over escalation pathways, and technological downtime disrupting electronic reporting platforms [17,29].

With each new health care cyberattack, novel forms of patient harm continue to emerge, with practitioners citing insufficient clinical preparedness and limited understanding of the clinical implications of IT disruptions as key factors in adverse events [17,21,30]. Public health research, clinical case reports, and media coverage document compelling patient and family narratives that illuminate how these cyberattacks materially affect care delivery and clinical outcomes [7-10,29-32]. In this study, we consolidate these stories, comprehensively analyzing documented experiences of cyber-induced clinical harm across nations, establishing a framework for understanding patient-level impacts of hospital cyberattacks across diverse clinical contexts. Specifically, this review addresses three questions: (1) what patient-level harms, categorized by clinical specialty, occur during hospital cyberattacks? (2) Which clinical populations are disproportionately vulnerable to these harms? And (3) how do patient-level harms identified in patient and provider narratives on social media compare with those reported in peer-reviewed case studies?

Through a systematic review of internationally published case studies supplemented by social media data mining, we present the first evidence synthesis of patient-level harms from health care cyberattacks. In doing so, this research aims to enhance population health modeling of cyberattack effects and reduce clinical uncertainty for frontline health care providers working under these conditions.

## Methods

### Part 1: Systematic Review, Search Strategy, and Information Sources

A PRISMA (Preferred Reporting Items for Systematic Reviews and Meta-Analyses; Checklist 1)-compliant systematic search was conducted across 5 databases: MEDLINE, Embase, PubMed, Scopus, and Web of Science (January 1, 2004-July 1, 2026; full search strategies available in Multimedia Appendix 1). Searches were conducted on July 1, 2026, and covered studies published between January 1, 2004, and July 1, 2026. Database-specific date limits were applied according to each platform’s available functionality. MEDLINE and Embase were selected as the 2 primary databases in accordance with Cochrane guidance; CENTRAL was not searched, as it indexes controlled trials rather than observational case studies [33]. Controlled vocabulary terms were mapped across MeSH and Emtree prior to the development of the search strategies. MeSH terms were applied in MEDLINE (Ovid) and PubMed, and Emtree terms were applied in Embase (Ovid), and free-text searching was used in Scopus and Web of Science, where subject headings were not supported [33]. No methodological search filters were applied because validated filters are primarily designed for randomized or comparative study designs and would likely reduce sensitivity for observational case reports and case studies. The search strategy is reported in accordance with the PRISMA-S (Preferred Reporting Items for Systematic Reviews and Meta-Analyses–Search) extension, with the completed checklist provided in Checklist 2 [34].

The review question followed an adapted PICO (Population, Intervention, Comparator, and Outcome) framework. The Comparator and Outcome elements were omitted because no unexposed control exists in naturalistic cyberattack research, and outcome terms may reduce recall [35,36]. Search strategies used 2 concept blocks—cyberattack types and health care settings—combined with Boolean operators (Multimedia Appendix 1). No study registries, gray literature databases, citation searching, or author contacts were used, and the search was not peer-reviewed by an information specialist. Prior to title and abstract screening, papers were filtered to ensure that they were peer-reviewed articles (excluding retracted studies and preprints), focused on human subjects (excluding veterinary research), and available in English.

### Eligibility Criteria

Studies were included if they described an empirical case study of a health care delivery organization (HDO) affected by a cyberattack and reported effects on clinical care or patient outcomes. Studies were excluded for the following reasons:

- Reason 1 (the domain focus was not health care): the study had to focus on cybersecurity breaches in the health care domain, thus excluding studies focused on other sectors (eg, water and energy) and digital health studies focused on tangential topics (eg, quality assurance [QA] and digital safety).
- Reason 2 (the study was not a case study focused on a clinical environment): the study had to focus on a cyberattack affecting a true clinical environment (hospital or health care services eg, laboratories), thus excluding papers written from a hypothetical perspective (eg, thought pieces on potential risks) and proof-of-concept articles on cybersecurity innovations or mitigations.
- Reason 3 (the study did not cover clinical impact or patient harms): the study had to focus on clinical impacts and potential patient harms (eg, morbidity and mortality) and therefore excluded studies focused purely on economic impacts or reputational damage.

### Data Collection, Selection, and Synthesis Process

Citations were imported into Rayyan (Rayyan Systems Inc) [37] for analysis by 3 independent reviewers (IS, JT, and CD), who processed abstracts under blind review and compared results to form the final list of included studies (Multimedia Appendix 2). Deduplication was performed in Rayyan using its automated and manual matching functions, and the search was not externally peer-reviewed. Included studies underwent full review for identification of study design, attack details, organizational characteristics, and direct patient harms and clinical care disruptions identified by the clinical members of the research team as a risk to patient safety (Multimedia Appendix 3). During data extraction, each identified patient-level harm was allocated to the most relevant clinical specialty by clinical members of the research team, with harms affecting multiple specialties assigned to each relevant specialty. Grouping by clinical specialty was chosen because it directly reflects our objective of identifying vulnerable patient groups and is clinically actionable for preparedness planning. This grouping was determined inductively from the evidence rather than prespecified.

Meta-analysis with quantitative synthesis was not performed because the included studies reported structurally incompatible outcome types—continuous metrics (eg, cardiac arrest survival rates), count data (eg, appointment cancellations), and qualitative narratives (firsthand accounts)—for which no common effect measure exists. A meaningful meta-analysis requires all studies to share essentially the same index of treatment effect and requires the combined analysis to yield a clinically interpretable result; neither condition is met here [38]. In addition, clinical diversity across 13 countries and the inclusion of mixed study designs further precluded a meaningful pooled estimate.

Synthesis without meta-analysis was therefore conducted in accordance with the SWiM (Synthesis Without Meta-analysis) reporting guideline, and the completed checklist is provided in Checklist 3 [39]. Rather than estimating intervention effects, this review catalogs patient-level harms reported across the included studies, which were synthesized as descriptive frequency counts per clinical specialty and data source. All 59 included studies contributed equally to this synthesis; no studies were prioritized or excluded on study design or risk of bias (Multimedia Appendix 2). Heterogeneity across the evidence base was examined informally by comparing the distribution of harms across sources, countries, and clinical domains as presented in Figure 1. The complete database of identified patient-level harms from these sources is published in a GitHub repository alongside this paper [31]. The complete study-level data extraction table is provided in Multimedia Appendix 3, while a summary of study characteristics is presented in Table S1 in Multimedia Appendix 2.

**Figure 1.**
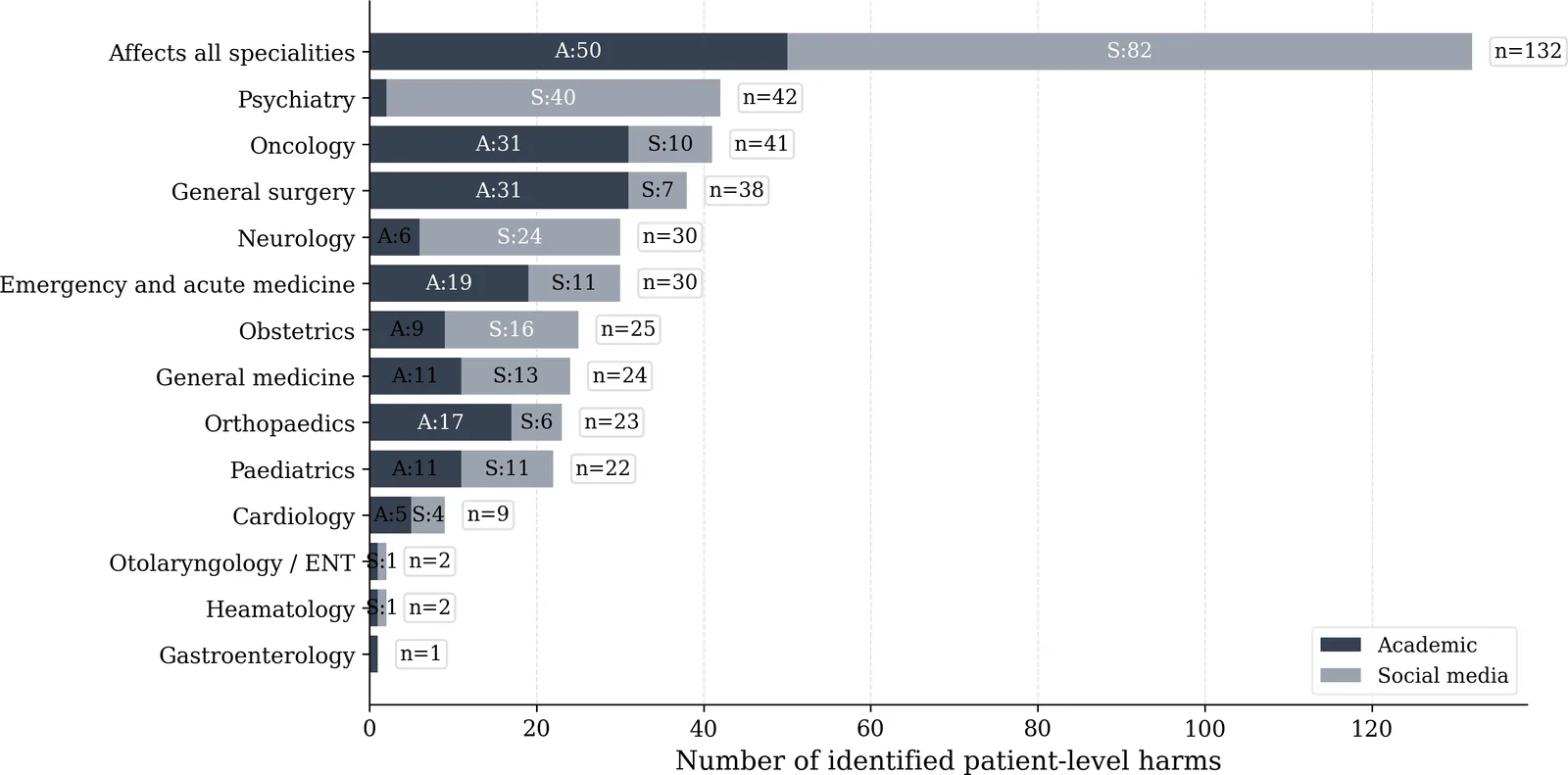
The distribution of reported patient-level harms (N) during health care cyberattacks by clinical specialty, comparing evidence from published case studies (academic literature) with harms identified from Reddit patient and provider narratives (health care subreddits, same period). Patient harms relevant across all specialties (eg, blood test delays) were categorized under “Affects all specialties,” while harms that may affect more than one specialty (eg, compromised operating room software) were allocated to multiple relevant specialties (eg, general surgery and obstetrics).

### Study Quality and Bias Assessment

To evaluate the quality of the extracted studies, we used the Mixed Methods Appraisal Tool (MMAT) for critical appraisal [40]. The MMAT provides a flexible framework for appraising qualitative, quantitative, and mixed methods studies and has been widely used in similar research [40,41]. Three researchers took part in the data collection, study selection, and approval process, with any disagreements resolved through discussion among the team. The results of the quality assessment are presented in the “Quality and Bias” subsection in the Results.

### Part 2: Social Media Data and Web Mining

To identify reported harms in social media data, we focused on Reddit (Reddit, Inc) patient support forums, as Reddit has previously been identified as a rich source of content for health care discussions [42,43]. To identify relevant posts, we first created a list of relevant “cyber terms” to act as the main search keywords for the mining algorithm and then collated a list of target “health care subreddits” with a medical or patient support theme for searching.

First, the “cyber terms” were selected from keywords extracted during the academic systematic review. During the evaluation of the academic papers in part 1, we noted the names of particular health care cyberattack exploits (eg, Conti Ransomware and DeepBlueMagic; Multimedia Appendix 3). Second, to form the list of target “health care subreddits,” clinical members of the research team formed a medical vocabulary list, which was used to identify relevant Reddit forums. A list of medical terms was initially formed from:

1. The American Medical Association list of medical specialties (eg, gastroenterology)
2. The Centers for Disease Control and Prevention (CDC) list of most common conditions in the United States (eg, asthma)
3. A list of medical treatments that were related to each condition identified in items 1 and 2 [2,44]

This approach resulted in a list of 2487 medical terms. Automated scripts were then used to interact with the Reddit API, searching for subreddits that contained the 2487 medical terms, returning a list of 3408 “health care subreddits” (eg, *r/dialysis* and *r/autismadhd*).

The codebook for data mining was created in Jupyter Notebook (version 7.3.2; Project Jupyter) and written in Python (the associated code is published in the open-source GitHub [31]). We mined the 3408 health care subreddits for posts that included the cyber terms, limiting the time span to match the academic review. A clinical member of the team reviewed each post to ensure clinical relevance and exclude bot activity. In keeping with similar social media research, all posts were deidentified, with usernames and any personal information removed, including details of location, hospital system, and employers.

The final list of social media posts describing cyberattack-induced patient harms was added to the database of academic data to form the full database of cyberattack-induced patient harms, published as the Cyberattack Impacts, Patient Harms & Emergency Response (CIPHER) dataset in the accompanying repository [31]. For consistency across the database, each identified patient-level harm was assigned to the most relevant clinical specialty (eg, loss of fetal monitoring systems assigned to obstetrics), except in cases where the harm may affect multiple or all specialties (eg, delays in coagulation profiles affecting multiple medical and surgical specialties). The database was structured to provide the following information on each patient-level harm: reference source (academic vs social), reference link, short title, description of harm, original quote from source, and clinical specialty [31]. A descriptive analysis was performed on the database to identify the total count of harms within each specialty and from each source (social media vs academic data) to identify medical domains with a higher frequency of reported harms.

### Ethical Considerations

The study methodology received a waiver of informed consent from the University of California San Diego Institutional Review Board (Protocol number: 812189), as all data were publicly available and were anonymized before integration into the dataset.

## Results

### Overview

The systematic review of academic data returned 16,829 publications, of which 5359 were duplicates. Following title and abstract screening, 878 reports underwent full-text eligibility assessment, of which 59 studies [11,13,45-101] met the inclusion criteria (Figure 2). Articles reported health care cyberattacks in Canada, Czechia, Finland, France, Ireland, India, Israel, Italy, Lebanon, New Zealand, South Africa, the United Kingdom, and the United States; the full list of case studies can be found in Table S1 in Multimedia Appendix 2. Across these articles, diverse components of the hospital infrastructure were affected, including loss of the computers themselves, access to the electronic health record, internal telephone, paging, electronic dictation systems, internet and intranet access, digital medication systems, anesthetic machines, patient and fetal monitoring systems, digital radiology, and computerized laboratory and pathology services [11,45-51]. From the evaluation of the 59 studies, 132 distinct patient-level harms were identified. In the same time period, we identified 779 Reddit posts, of which 184 were validated to contain information on patient harms relating to hospital cyberattacks, leading to a total database of 316 patient-level harms [31].

On examining the distribution across clinical specialties, we found that social media data revealed a different clinical distribution of patient harms compared to the academic sources, indicating that academic data alone may not suffice in painting the true landscape of patient-level harms during cyberattacks (Figure 1). In the academic data, patient harms were most commonly identified in emergency and acute medicine, general surgery, and oncology, while harms affecting patients in psychiatry, neurology, general medicine, and obstetrics/women’s health more frequently arose on social media (Figure 1). An extract from the full database of patient-level harms within each specialty can be found in Multimedia Appendix 4, where harms are categorized by data source and each social media quote is numbered (eg, Q1.1), facilitating their reference throughout the text that follows.

**Figure 2.**
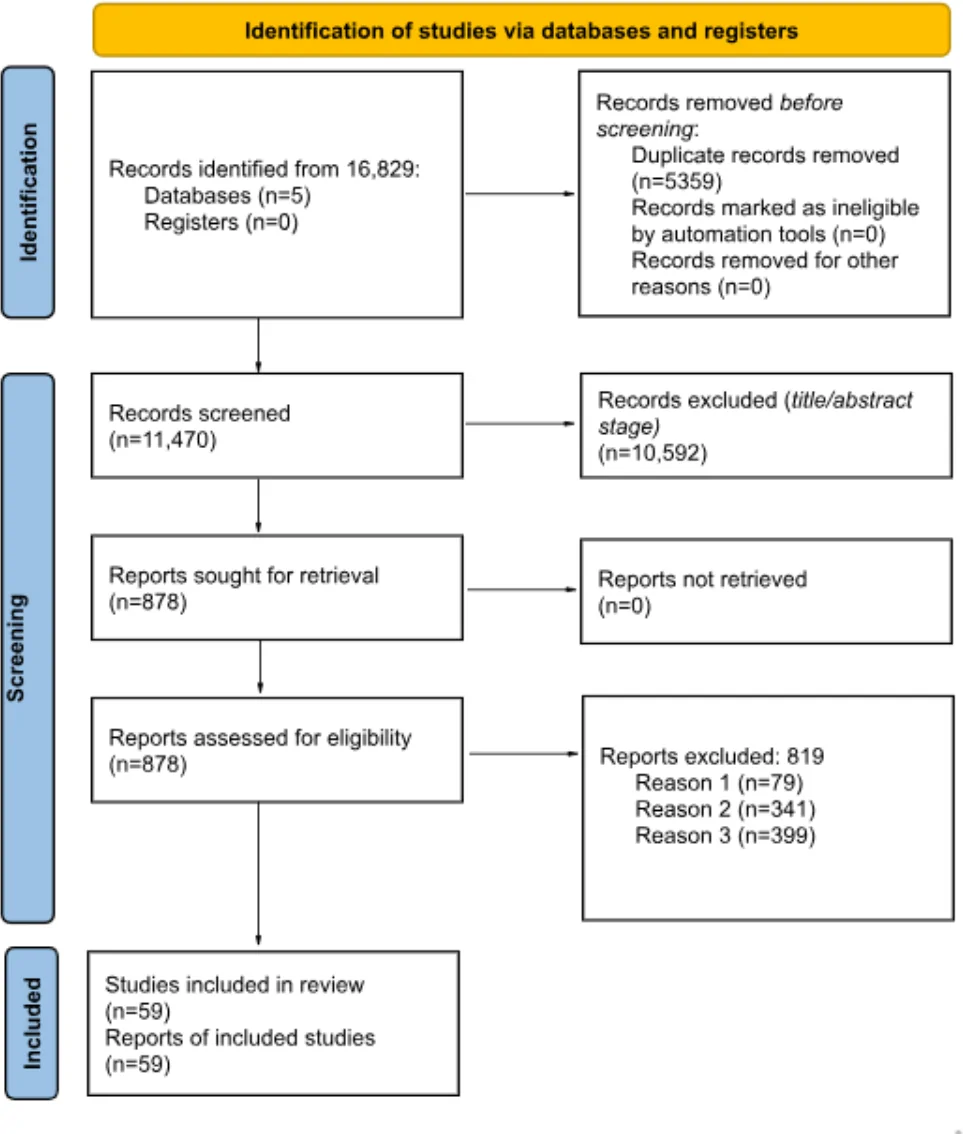
PRISMA (Preferred Reporting Items for Systematic Reviews and Meta-Analyses) 2020 flow diagram for systematic reviews including searches of databases and registers only. Broad title and abstract exclusion categories were (1) not focused on the health care domain, (2) not an empirical case study of a health care delivery organization, or (3) not reporting clinical impacts or patient harms (full details provided in Methods, Part 1).

### Cyberattack-Sensitive Conditions and At-Risk Patient Groups

Across the academic and social media data, specific clinical groups recurred, highlighting their vulnerability to cyberattack-induced effects. Multimedia Appendix 4 provides granular information on the identified harms within the clinical groups, which included patients with chronic pain, patients receiving obstetric care, emergency and trauma cases, those receiving end-of-life care, newborns, patients with cancer (especially those dependent on blood transfusions), patients receiving surgical care, patients with orthopedic conditions, and patients with psychiatric conditions.

### Emergency, Acute, Intensive Care, and Blood Transfusion Services

Academic studies emphasized the risks faced by patients with time-sensitive conditions during cyberattacks (eg, stroke) [45-61]. Pham et al [55] detailed the shift in cardiac arrest survival outcomes at facilities neighboring the victim hospital, demonstrating that “survival with favorable neurological outcomes” for out-of-hospital cardiac arrests (OHCAs) fell from 40% to 4.5% during the cyberattack period. These statistical insights are complemented by parallel papers that provide narrative accounts of the heightened risks posed to patients dependent on timely interventions, including those experiencing strokes, sepsis, and myocardial infarctions [11,55,56]. Neprash et al [62] examined in-hospital mortality across all clinical conditions during hospital ransomware attacks affecting the United States between 2016 and 2021 and estimated that cyberattacks resulted in the deaths of 69‐76 patients with Medicare, or an estimated 1 Medicare death per month, over the course of the study period.

Dameff et al [59] reveal the regional spread of these harms, identifying that ransomware disruptions extend to emergency rooms adjacent to the target hospital, resulting in inflated waiting room times, increased ambulance arrivals, and higher rates of patient self-discharge. These insights are supported by further research from Abouk and Powell [60], who found that while there was a temporary decrease in emergency department (ED) visits at hospitals targeted by ransomware attacks, these shifts manifested in increased ED visits and inpatient admissions at unattacked nearby hospitals, exposing the regional consequences that must be considered in cyberattack response planning [59]. Neprash et al [56] examined the increased risk for acute patients in rural settings, finding that “travel time and distance to the closest nonattacked hospital was 4‐7 times greater for rural ransomware-attacked hospitals than for urban ransomware-attacked hospitals” [56].

Researchers reported further acute care disruptions resulting from (1) the loss of intranet protocols for urgent conditions, (2) increased blood test turnaround times delaying diagnosis and management, (3) overcrowding in the emergency room, and (4) the need to transfer patients with trauma away from victim hospitals [11,48-59,63] (Multimedia Appendix 4). The patients with trauma were identified as being at heightened risk in instances where blood transfusion services were impacted, preventing effective delivery of major hemorrhage protocols [13,64]. Social media data revealed acute care staff discussing these challenges in real-time, with practitioners detailing (1) the loss of catheter labs and anesthesia services necessitating patient transfers (Q1.1; Multimedia Appendix 4), (2) laboratory issues preventing treatment of heart attacks (Q1.2: “Troponin delayed by 4 hours*”),* (3) CT radiographers not being able to check for contrast allergy (Q1.3), (4) delayed time-critical results for patients with “brain hemorrhages and blood clots” (Q1.4), (5) the need to treat people “in the lobby” due to overcrowding (Q1.5), and (6) delays in administering medication due to electronic medical record (EMR) downtime (Q1.6). Alongside delays in medication administration, academic data highlighted the increased risk of medical allergies, showcasing that if allergy details were not detected at admission, they were unlikely to be picked up on the ward [49]. Patients on cytotoxic drug regimens were at particular risk, exemplified by oncological specialists, who described the critical challenge of laboratory delays when monitoring high-dose methotrexate protocols [51]. The challenge of laboratory delays was further compounded by heightened errors during this period, as lab staff described increased QA issues related to incorrect names and case numbers on specimens requiring evaluation [65].

These challenges are particularly pertinent in intensive care units (ICUs), where dose calculations, titration logic, and trend interpretation are frequently delegated to computing systems, raising the risk of medication safety incidents during downtime [66]. Choudhuri et al [66] also note that ICUs are often a hospital’s most concentrated internet of medical things (IoMT) environment, with heavy reliance on networked ventilators, monitors, infusion pumps, and dialysis machines, resulting in a high-risk attack surface. Neprash et al [62] described a 34%‐38% increase in in-hospital mortality during cyberattack-induced system downtime, also highlighting the heightened impact on patients in the ICU and those with multiple comorbidities, who were demonstrated to have larger mortality effect sizes in subgroup analysis. In cases where the cyberattack disrupted hospital communication systems (eg, paging and telephones) and access control systems (eg, door access), staff described patient deaths occurring while practitioners were unable to contact the necessary medical staff during life-threatening emergencies (Multimedia Appendix 4).

This study revealed a further dimension to cyberattack-sensitive conditions, uncovering the heightened vulnerability of patients who are both (1) dependent on digital systems for medical maintenance and (2) have the potential to rapidly decline. For instance, social media data described outpatients with diabetes losing access to Dexcom sensors for their maintenance care (Q1.25), while in inpatient settings clinicians reported the need to transfer patients with diabetic ketoacidosis who were critically ill [102] (Q1.14). Patients with diabetes are thus hit by 2-fold risks during cyberattacks, they may lose access to vital maintenance care in the community and face suboptimal life-critical care in the acute setting if they deteriorate. Similarly, patients with cancer were at a raised risk due to their dependence on digitally enabled radiation and chemotherapy systems, without which patients risked loss of tumor control and deterioration [45,48,51] (Multimedia Appendix 4). Finally, delays in outpatient care lead to clinical deterioration for diverse chronic medical conditions, captured in the case of one patient with asthma, who described how “breathing burns” after their HDO was hit by ransomware, leading to inaccessible medications and worsening of their condition (Multimedia Appendix 4).

### Oncology Services, Patients With Cancer, and Palliative Care

Several studies detailed cyberattack disruption to linear accelerators for radiation treatment and lost imaging services preventing adequate tumor site localization [46-48] (Multimedia Appendix 4). Across multiple studies, researchers detailed the heightened risks for patients with cancer who were in the middle of their radiation course of fractionated doses, where lost data on historic fractions and treatment interruptions potentiated worse oncologic outcomes [51,58,67-69]. O’Shea et al [69] described 5 case studies of patients with cancer receiving original external beam radiotherapy (EBRT) during the 2021 cyberattack in Ireland, who experienced treatment disruption, putting them at risk of rapid tumor cell repopulation and decreased tumor control probability (TCP). Beyond radiation technology, compromised cloud-based chemotherapy platforms meant clinicians had to reconstruct doses from memory, creating risks of toxic drug errors and the cancellation of care for many [57,68-71]. One paper described a framework for “Ethical rationing of chemotherapy” that was adopted during their cyberattack due to limited resources, using a tiered approach for prioritizing patients based on impending organ compromise and escalating pain [51]. Furthermore, issues with operating room software, oncology information systems, and restrictions on intraoperative pathology led to widespread cancellation of tumor surgeries [46-48,57,68-71] (Multimedia Appendix 4). Ades et al [51] showcased this graphically, demonstrating the dip in both in-person and telemedicine appointments, alongside the average infusion visit rate, during the cyberattack period and the weeks that followed. The authors reported a 41% decrease in total outpatient volume overall immediately following the cyberattack, with infusion center visits initially dropping by 63% [51].

Multiple researchers identified the heightened impact on patients with breast cancer due to canceled imaging screening and biopsies and lost biopsy results preventing specialists from determining whether definitive surgeries for tumors had been effective (running the risk of recurrence) [50,72,73]. Perry et al [72] detailed the reduction in care quality at a breast imaging unit during a cyberattack, where staff were unable to interpret screening mammography scans, leading to delays that breached the Food and Drug Administration’s (FDA’s) Mammography Quality and Standards Act (MQSA) and 30-day limit for patient results. For patients with severe and advanced disease, cyberattacks significantly reduced clinical trial access and potential life-saving treatment [74]. One relative on social media explained how a cyberattack led to the cancellation of a patient’s follow-up screening, following which there was a recurrence of the patient’s small cell lung cancer that was missed and the patient died shortly after (Q1.17). Ades et al [51] identified further oncological subpopulations who endured increased harms during the cyberattack period, including (1) neuro-oncological cognitively impaired populations (who rely heavily on digital automated systems eg, appointments and prescriptions), (2) stem cell transplant patients (due to delays in transplant initiation), and (3) research patients (due to pauses in trial activity).

Issues providing end-of-life comfort care were reported across both the academic and social media data. Keogh et al [48] described the difficulties encountered by staff trying to treat symptomatic dying patients during EMR downtime, a problem that was reflected on social media with clinicians detailing delays in transitioning their patients to palliative protocols, “It took me 6 hours to get my patient transitioned to comfort care and get morphine orders” [Multimedia Appendix 4]. Finally, one report from Devi [75] described previously unseen confidentiality harms for patients with cancer through the case of the BlackCat attackers who targeted Lehigh Valley Health Network and leaked intimate medical images onto the dark web. The images were sensitive, containing nude photographs of patients with breast cancer imaged from the waist up, with their release causing significant distress for victims [75,103].

### Surgical Cases and Patients With Orthopedic Conditions

Beyond cancer operations, reports from multiple countries demonstrated that immediate operating room downtime affected patients undergoing diverse surgical procedures, due to outages in (1) surgical scheduling software, (2) intraoperative imaging, (3) preoperative note systems, (4) pyxis/drug delivery systems, and (5) anesthetic machines [48,61,76,77] (Multimedia Appendix 4). Zhao et al [77] described this for patients scheduled to undergo angiography, “If you are going to do an angio on someone who has previous angios and can’t look at those images, that is a significant negative impact.” Hoffman and Baker [49] summarized challenges for spinal surgeons unable to access magnetic resonance imaging (MRI) or computed tomography (CT) imaging during spinal procedures, presenting the significant risk of wrong-level surgery or suboptimal decompression. Feeley et al [61] reported barriers for patients with orthopedic conditions when digital templating software was lost, preventing selection of implant size and position among patients with orthopedic conditions.

Zhao et al [77] highlighted harms for patients with orthopedic conditions from the elimination of EMR safety nets, detailing cases of missed injuries in patients where it was not possible to readily review the relevant imaging. McCarthy et al [78] examined orthopedic subpopulations, diving into the deterioration in ward-based care for patients with hip fracture, who experienced an increased rate of hospital-acquired pressure ulcers between the precyberattack (2.1%) period to the postcyberattack aftermath (3.1%). Further papers written from a surgical perspective revealed key cases in which missed or confused medication histories could have significant adverse consequences, such as missing a history of anticoagulation in an older adult patient with a fracture [49]. Feeley et al [61] detailed these challenges at the regional level, where patients who would usually be reviewed by trauma on-call teams at regional units were severely limited in their off-site assessment due to the impacted imaging servers and laboratory results systems. In South Africa, researchers noted that the specialties of general surgery and nephrology faced the highest rate of delayed diagnosis due to compromised testing facilities [79]. Social media data revealed wider issues in the outpatient setting, where relatives reported they could not obtain postoperative pain medications for recently discharged patients undergoing surgical procedures due to compromised e-prescribing systems (Multimedia Appendix 4).

### Patients Receiving Obstetric Care, Birth Plans, and Pediatric Cases

The harms affecting patients receiving obstetric care spanned issues obtaining fetal growth scans during pregnancy, disruptions on the labor ward during ransomware attacks, and difficulties obtaining care for miscarriages (Multimedia Appendix 4). Gabbay-Benziv et al [76] detail specific challenges in the delivery room and, similar to other academic sites, describe the decision to cancel cesarean sections locally and transfer high-risk patients receiving obstetric care elsewhere. On social media, we saw patients discussing the impact of ransomware on their labor experiences, with patients describing delays in ward-based administration of medications and inability to contact health care practitioners (Q1.31‐1.35). In one narrative, a patient was at 38 weeks’ gestation when their local maternity services went down, leading to cancellation of their appointments and inability to contact the birthing center, resulting in significant concerns as the patient developed contractions and signs consistent with labor (Q1.34). Outside the hospital setting, women reported distress from not being able to access birth control, captured in Q1.30, “on day 2 with no birth control and I’m freaking out a lot,” and concerns regarding delayed cervical smear results (Q1.30 and Q1.38).

Staff also reported concerning barriers to life-critical care on obstetric and neonatal wards, with one post alleging an infant death related to issues with fetal monitors, “the monitor was tracking the mother’s heart rate, and they didn’t realize it wasn’t the baby’s for a while. The baby was down for so long it had no brain activity after birth*”* (Q1.36). Additional staff reflected on the case of a *“*baby ransomware death*”* in the United States that was reported in several media outlets, describing similar cases within their own clinical practice (Q1.36‐1.37) [2]. Alongside disruptions to administering treatment, staff struggled to provide birth certificates for newborns, meaning that patients left the hospital without newborn registration, raising safeguarding risks for newborns from vulnerable backgrounds (Multimedia Appendix 4).

### Patients With Neuropsychiatric, Psychiatric, and Neurological Conditions

The high frequency of neurology posts (n=30) stemmed from patients with migraine and chronic pain unable to access regular treatments in the community (Figure 2 and; Multimedia Appendix 4). Similarly, prescription issues caused significant difficulties for patients with mental health conditions, who were unable to obtain maintenance medications, with some of whom reported new side effects after clinicians switched them to alternative treatments (Q1.57‐1.58). In addition to lost medication access, patients reported a worsening of symptoms due to (1) cyberattack-induced anxiety and trauma symptoms, (2) barriers to obtaining psychotherapy as virtual therapy platforms collapsed, and (3) decisions to opt out of therapy due to fears of data breaches and compromised confidentiality (Multimedia Appendix 4). In some cases, such data breaches involving therapy records have been linked to patient deaths, with the Vastaamo hack in particular being highlighted in academic data and discussed among patients receiving psychiatric care on Reddit [52] (Q1.60). In this case, the hacker targeted psychotherapy platforms and then blackmailed patients after obtaining their therapy notes, contributing to one patient’s suicide, a case that has now been covered by wider news outlets and literature in Finland [52,104] (Q1.60).

### Cyberattack Effects on Hospital Infrastructure, Staff, and External Settings

Reports from frontline staff and health care managers revealed wider impacts of the cyberattack on hospital infrastructure and outpatient services, which contributed to additional downstream effects (Multimedia Appendix 5). In France, the Ryuk ransomware attack on Dax General Hospital disabled the hospital’s sterilization service, such that the loss of digital traceability precluded the verification of safe and sterilized equipment, leading to surgical cancellations [70]. In South Africa, Cassim and Chapanduka [50] described clinicians turning to unconventional methods when hospital communication systems were lost (eg, Gmail and social media), violating data protection protocols and risking patient privacy. Social media data uncovered wider issues across hospital infrastructure: (1) practitioners expressed safety concerns around lost closed-circuit television (CCTV) on high-risk psychiatry wards, (2) the loss of laboratory networked fridges affected treatment availability (eg, blood products), (3) staff lost patients in the hospital due to failures in bed monitoring systems, (4) broken paging and door access systems prevented life-saving care, (5) failures in the pneumatic tube system stalled specimen evaluation, and (6) inoperable air conditioning units worsened patient care environments [63,78,80].

Several papers provided details on the communication breakdown that occurred during a cyberattack within their hospitals, describing failures in internal communication systems (email servers, centralized paging, landline phones, and electronic dictation systems), external communication with patients (centralized centers for incoming patient calls and calls from clinics to patients), and external communication with other health care sites (faxes and Wi-Fi networks and wired networks for all devices) [51,65]. On the wards, nurses reported issues with failures in patient call alarms, causing staff to resort to less effective measures such as providing patients with bells and whistles to gain medical attention (Q1.73). In several instances, staff stated they were dissuaded from reporting issues related to the cyberattack due to fears of losing their jobs (Q1.70‐71). Other staff members turned to Reddit to discuss issues of burnout and their intentions to quit, as the cyberattack pressures made clinical environments unbearable (Multimedia Appendix 4).

Outside the hospitals, data revealed cyberattack impacts on emergency services, with frontline US staff reporting loss of “the 911 phone line” and UK practitioners detailing all systems going down in primary care (Multimedia Appendix 5). Additionally, patients reported loss of “Health at Home” services, with one patient describing the suspension of home treatments for her inflammatory bowel disease during the cyberattack period (Multimedia Appendix 5).

### Study Quality and Bias Assessment

Our study quality assessment, performed using the MMAT, indicated generally high quality across the 59 included studies, with the breakdown across MMAT subsections provided in Table 1 [40]. Of the 59 studies, 40 used qualitative approaches, 14 used quantitative descriptive methods, and 5 used mixed methods. A total of 50 of the 59 studies presented clear research questions or objectives, and where this was not the case, this was because the authors provided a narrative of their cyberattack encounter, where the implicit aim was to share experience with the community, as opposed to addressing specific research questions [68,74]. Across the studies, appropriate data were collected for addressing the aims and questions, and the included studies demonstrated a low risk of bias (Table 1).

**Table 1.** Quality and bias assessment of included studies describing the impact of health care cyberattacks on clinical operations and patient outcomes, assessed using the MMAT^a^ [40]. Article number corresponds to those listed in the full table of articles provided in Multimedia Appendix 3.

Number	Reference	MMAT components																
		All studies		Qualitative Studies					Quantitative descriptive Studies					Mixed methods studies				
		S1^b^	S2^c^	1.1^d^	1.2^e^	1.3^f^	1.4^g^	1.5^h^	4.1^i^	4.2^j^	4.3^k^	4.4^l^	4.5^m^	5.1^n^	5.2^o^	5.3^p^	5.4^q^	5.5^r^
1	Moore et al [68]	1^s^	1	1	1	1	1	1	—^t^	—	—	—	—	—	—	—	—	—
2	Ghafur et al [53]	1	1	—	—	—	—	—	1	1	1	1	1	—	—	—	—	—
3	Stowman et al [73]	1	1	1	1	1	1	1	—	—	—	—	—	—	—	—	—	—
4	Stowman et al [81]	1	1	1	1	1	1	1	—	—	—	—	—	—	—	—	—	—
5	Goodwin et al [80]	1	1	1	1	1	1	1	—	—	—	—	—	—	—	—	—	—
6	Perry et al [72]	1	1	1	1	1	1	1	—	—	—	—	—	—	—	—	—	—
7	Klatt [82]	1	1	1	1	1	1	1	—	—	—	—	—	—	—	—	—	—
8	Haase et al [83]	2^u^	3^v^	3	3	1	1	1	—	—	—	—	—	—	—	—	—	—
9	Fink and Gresko [84]	2	3	3	3	3	1	1	—	—	—	—	—	—	—	—	—	—
10	Powell et al [85]	2	3	3	3	3	1	1	—	—	—	—	—	—	—	—	—	—
11	Devi [75]	2	3	3	3	3	1	1	—	—	—	—	—	—	—	—	—	—
12	Pontier et al [47]	1	1	1	1	1	1	1	—	—	—	—	—	—	—	—	—	—
13	Cassim and Chapanduka [50]	1	1	1	1	1	1	1	—	—	—	—	—	—	—	—	—	
14	Keogh et al [48]	1	1	—	—	—	—	—	—	—	—	—	—	1	1	1	1	1
15	Santalo et al [86]	1	1	1	1	1	1	1	—	—	—	—	—	—	—	—	—	—
16	Van boven et al [11]	1	1	1	1	1	1	1	—	—	—	—	—	—	—	—	—	—
17	Harrison et al [71]	1	1	1	1	1	1	1	—	—	—	—	—	—	—	—	—	—
18	Nelson et al [57]	1	1	1	1	1	1	1	—	—	—	—	—	—	—	—	—	—
19	Zhao et al [77]	1	1	1	1	1	1	1	—	—	—	—	—	—	—	—	—	—
20	Gourd [87]	2	3	3	3	3	1	1	—	—	—	—	—	—	—	—	—	—
21	Zhao et al [88]	1	1	1	1	1	1	1	—	—	—	—	—	—	—	—	—	—
22	Leonard et al [89]	1	1	—	—	—	—	—	1	1	1	1	1	—	—	—	—	—
23	Strong et al [90]	1	1	1	1	1	1	1	—	—	—	—	—	—	—	—	—	—
24	Hoffman and Baker [49]	1	1	1	1	1	1	1	—	—	—	—	—	—	—	—	—	—
25	Lacobucci [64]	2	3	3	3	3	1	1	—	—	—	—	—	—	—	—	—	—
26	Aljaidi et al [91]	1	1	—	—	—	—	—	1	1	1	1	1	—	—	—	—	—
27	Dameff et al [59]	1	1	—	—	—	—	—	1	1	1	1	1	—	—	—	—	—
28	Abouk and Powell [60]	1	1	—	—	—	—	—	1	1	1	1	1	—	—	—	—	—
29	Pham et al [55]	1	1	—	—	—	—	—	1	1	1	1	1	—	—	—	—	—
30	Chen et al [92]	1	1	1	1	1	1	1	—	—	—	—	—	—	—	—	—	—
31	Zhao et al [101]	1	1	1	1	1	1	1	—	—	—	—	—	—	—	—	—	—
32	Duffy et al [93]	1	1	—	—	—	—	—	1	1	1	1	1	—	—	—	—	—
33	Stowman and Kalof [94]	2	3	3	3	3	1	1	—	—	—	—	—	—	—	—	—	—
34	Tarka et al [95]	1	1	—	—	—	—	—	—	—	—	—	—	1	1	1	1	1
35	Filipec and Plasil [46]	1	1	1	1	1	1	1	—	—	—	—	—	—	—	—	—	—
36	Slattery et al [96]	1	1	—	—	—	—	—	1	1	1	1	1	—	—	—	—	—
37	Oliver et al [45]	1	1	1	1	1	1	1	—	—	—	—	—	—	—	—	—	—
38	Harvey et al [74]	1	1	—	—	—	—	—	1	1	1	1	1	—	—	—	—	—
39	Russell et al [97]	1	1	—	—	—	—	—	—	—	—	—	—	1	1	1	1	1
40	Kelly et al [54]	2	3	3	3	3	3	1	—	—	—	—	—	—	—	—	—	—
41	Feeley et al [61]	1	1	1	1	1	1	1	—	—	—	—	—	—	—	—	—	—
42	Neprash et al [56]	1	1	—	—	—	—	—	1	1	1	1	1	—	—	—	—	—
43	Abbou et al [13]	1	1	1	1	1	1	1	—	—	—	—	—	—	—	—	—	—
44	Mashinchi et al [98]	1	1	1	1	1	1	1	—	—	—	—	—	—	—	—	—	—
45	Gabbay‐Benziv et al [76]	1	1	—	—	—	—	—	1	1	1	1	1	—	—	—	—	—
46	Looi et al [52]	1	1	1	1	1	1	1	—	—	—	—	—	—	—	—	—	—
47	Chen et al [99]	1	1	—	—	—	—	—	1	1	1	1	1	—	—	—	—	—
48	Ades et al [51]	1	1	—	—	—	—	—	—	—	—	—	—	1	1	1	1	1
49	Lippi and Ferrari [58]	1	1	1	1	1	1	1	—	—	—	—	—	—	—	—	—	—
50	Choudhuri et al [66]	1	1	3	3	3	1	1	—	—	—	—	—	—	—	—	—	—
51	Neprash et al [62]	1	1	—	—	—	—	—	1	1	1	1	1	—	—	—	—	—
52	Davids et al [79]	1	1	—	—	—	—	—	—	—	—	—	—	1	1	1	1	1
53	McCarthy et al [78]	1	1	—	—	—	—	—	1	1	1	1	1	—	—	—	—	—
54	Faul et al [67]	1	1	1	1	1	1	1	—	—	—	—	—	—	—	—	—	—
55	Frisch et al [65]	1	1	1	1	1	1	1	—	—	—	—	—	—	—	—	—	—
56	Al Haddad et al [63]	1	1	1	1	1	1	1	—	—	—	—	—	—	—	—	—	—
57	O’Shea et al [69]	1	1	1	1	1	1	1	—	—	—	—	—	—	—	—	—	—
58	Rajput et al [100]	2	3	3	3	3	1	1	—	—	—	—	—	—	—	—	—	—
59	Fabrice et al [70]	1	1	1	1	1	1	1	—	—	—	—	—	—	—	—	—	—

a MMAT: Mixed Methods Appraisal Tool.

b Question for S1: are there clear research questions?

c Question for S2: do the collected data allow addressing the research questions?

d Question for 1.1: is the qualitative approach appropriate to answer the research question?

e Question for 1.2: are the qualitative data collection methods adequate to address the research question?

f Question for 1.3: are the findings adequately derived from the data?

g Question for 1.4: is the interpretation of results sufficiently substantiated by data?

h Question for 1.5: is there coherence between qualitative data sources, collection, analysis, and interpretation?

i Question for 4.1: is the sampling strategy relevant to address the research question?

j Question for 4.2: is the sample representative of the target population?

k Question for 4.3: are the measurements appropriate?

l Question for 4.4: is the risk of nonresponse bias low?

m Question for 4.5: is the statistical analysis appropriate to answer the research question?

n Question for 5.1: is there an adequate rationale for using a mixed methods design to address the research question?

o Question for 5.2: are the different components of the study effectively integrated to answer the research question?

p Question for 5.3: are the outputs of the integration of qualitative and quantitative components adequately interpreted?

q Question for 5.4: are divergences and inconsistencies between quantitative and qualitative results adequately addressed?

r Question for 5.5: do the different components of the study adhere to the quality criteria of each tradition of the methods involved?

s 1: Yes.

t Not applicable.

u 2: No.

v 3: Can’t tell.

## Discussion

### Principal Findings

The evidence identified in this review demonstrates that health care cyberattacks are associated with diverse patient-level harms extending well beyond service disruption, affecting multiple clinical specialties and care settings. Health care cyberattacks cause measurable, clinically significant patient harms across diverse care settings and specialties. This review synthesizes evidence from 59 empirical case studies spanning 13 countries, supplemented by social media narratives from 3408 health care subreddits, providing the first transnational, clinically focused analysis of patient-level harms during hospital cyberattacks and resulting in an open-source database of cyberattack-induced patient harms categorized by clinical departments and technical domains. We uncovered reports of patients experiencing respiratory distress when ransomware blocked their access to asthma drugs, heard about the physical challenges faced by laboring mothers when attacks interrupted their birth plans, and learned of untimely patient deaths due to missed screenings during IT outages [72,73,76]. Across both academic and social media data sources, patient groups found to be at repeatedly at risk of cyberattack-induced harm included patients with emergency and trauma conditions, patients receiving obstetric care, patients with chronic pain, patients receiving palliative care, newborns, patients with cancer, patients receiving surgical and orthopedic care, and those requiring long-term psychiatric care.

### The Threat Landscape and Shifting Vulnerabilities

Across nations, clinical teams that had experienced cyberattacks reported that cybersecurity had long been considered a secondary issue in their hospitals, a perspective that shifted dramatically following the cyberattack events [46-51,77]. Consistently, researchers highlighted pressing concerns relating to patient safety, describing delays in the treatment of emergency conditions; disruption to ward-based, outpatient, and palliative care; and suboptimal clinical management due to loss of imaging and laboratory systems, operating rooms, and compromised treatment modalities [46-60]. While the rising number of cyberattacks was identified as a key concern, researchers also highlighted the shifting vulnerabilities among the health care workforce. Zhao et al [77] described the challenges faced by more junior members of the team (residents), trained entirely on digital systems, who struggled with the transition to paper-based methods. Generational differences also emerged on social media, where younger staff on nursing threads discussed the distress imposed by cyberattacks, which they felt was less understood by older adult nurses who were more adept at returning to nondigital workflows. As health care becomes increasingly digitized, this generational vulnerability will deepen, and workforce preparedness for IT downtime must be incorporated into institutional resilience planning.

### Cyberattack-Sensitive Conditions and at-Risk Patient Groups

Our comparative analysis of academic and social media data revealed 2 distinct categories of cyberattack-sensitive patients. The first consisted of patients harmed by time-critical conditions, such as those experiencing stroke, cardiac arrest, sepsis, or major trauma, for whom delays of minutes to hours are clinically decisive [46-59]. The evidence for this group was strongest in the academic literature, drawn from the highest-quality quantitative studies in our appraisal (Table 1), including Pham et al [55], who found a fall in survival with favorable neurological outcomes for patients with cardiac arrest presenting to hospitals neighboring a ransomware-affected institution.

The second and less recognized category comprised patients defined by the intersection of digital dependence and the potential for rapid clinical decline. Unlike the patient with trauma who may be unlucky in experiencing their injury during a cyberattack, the patient entering the compromised hospital with diabetic emergencies (eg, diabetic ketoacidosis) may have landed there due to the cyberattack disrupting their outpatient monitoring and medication access. Similarly, patients with cancer dependent on digitally enabled chemotherapy and radiotherapy platforms encountered compounded risk, where disruption to outpatient clinics and infusion services may potentiate acute presentations and oncological emergencies [45,48,51]. Understanding these cascading pathways, through which downtime across one part of the health care system generates acute presentations elsewhere, is essential for identifying which patients may be at most risk and for anticipating demand surges during an attack

This framework has direct implications for clinical preparedness. The most severe patient harms result not from a single system failure but from the unanticipated combined effects of diverse digital failures occurring simultaneously across different points in the health care system. Early cross-departmental communication to identify cyberattack-sensitive patient groups and anticipate the downstream effects of one department’s disruption on another should be a core component of institutional cyberattack response planning.

### Population Health, Long-Term Effects, and Health Equity

The population health consequences of health care cyberattacks extend well beyond the acute period of disruption. The deferral of diagnostic testing, screening, and nonurgent investigations during cyberattacks has implications for the long-term monitoring of chronic disease that current academic metrics fail to capture [47,86,88]. Missed mammograms, delayed cervical smears, and canceled cancer reviews create latent harms that may only become apparent months later; social media data included one account of a patient whose small cell lung cancer recurrence was missed following a cyberattack-related cancellation of follow-up screening [72,73]. In Israel, Abbou et al [13] described “whoever arrives*”* policies adopted when patient lists became inaccessible, highlighting how the loss of scheduled care creates conditions in which patients most in need of proactive surveillance are likely to be missed. The cancellation of elective procedures, a common mitigation strategy deployed by sites, carries its own downstream risks, with Hoffman and Baker [49] documenting a 25% increase in patients waiting 4 months for elective surgery in New Zealand following a ransomware attack. The authors also highlighted the physical risks these delays impose, alongside the psychological stress, whereby these decisions can lead to downstream clinical effects such as previously “minor” complaints progressing to more severe and inoperable conditions (eg, rotator cuff injuries) [49]. At the population level, Powell et al [85] identified the downstream population health risks associated with loss of infection control systems in hospitals during cyberattacks, where reduced capacity for prevalence testing and loss of COVID-19 screening results heightened the risk of inappropriate placement of patients and potential exposure to infectious diseases on wards. Finally, multiple authors referenced the risks to patients stemming from staff burnout and long-term implications of quitting and workforce attrition [85] (Q1.75‐1.76).

Another cornerstone of public health is health equity [105]. In this article, we have identified the heightened risk during hospital cyberattacks of medication errors, missed injuries, and misdiagnosis, phenomena known to fall along demographic lines in standard clinical practice [105,106]. In terms of socioeconomic disparities, Multimedia Appendix 5 demonstrated issues stemming from prescription prices dramatically increasing during cyberattacks due to coupon failures, and patients with Medicaid finding themselves unable to obtain coverage. The effect of cyberattacks on health care inequalities is a relatively unexplored area; however, the high number of harms affecting historically marginalized groups (eg, women, patients with mental health conditions, and those with chronic conditions) suggests that greater research is needed in this area. For all patient groups, the loss of follow-up systems puts “hard-to-reach groups” at higher risk of being “lost to services,” and in the pediatric setting, we heard of specific challenges in newborn care as staff lost the ability to register new births, raising safeguarding concerns [13]. Further research that examines the public health impact of cyberattacks through the lens of health equity would be valuable, taking into account the differential impact of issues such as the loss of group-specific reference ranges for laboratory tests, higher rates of medical error, and widespread access issues for community services.

### Measuring Cyberattack Harms and the Value of Social Media Data

Social media shed light on gaps in the academic literature on cyberattack impacts, raising the question of how we are detecting and measuring cyberattack-induced patient harms. Academic authors have typically quantified cyberattack impacts using coarse institutional metrics, patient volume, injury severity scores, length of stay, and mortality, which may fail to capture downstream harms, community-level effects, and the subjective experience of care disruption [53,77,99]. Zhao et al [77] took the approach of comparing yearly scores in patient volume, injury severity score, length of stay, and mortality, finding no difference between the years with and without a ransomware attack, a null result from one of the more methodologically rigorous studies in our appraisal (Table 1), which raises the possibility that the metrics favored by higher-quality quantitative designs may be poorly suited to detecting the harms documented in this review. These measures may fail to account for downstream effects (eg, reduced prognosis due to canceled surgeries) and do not cover the complaints we hear from patients on social media regarding cyberattack effects on outpatient services, distressful ward-based experiences, symptom deterioration due to reduced medication access and cyberattack anxieties, and disease progression due to missed screenings (Multimedia Appendix 4).

This discrepancy may be partly explained by the underreporting bias inherent to cyberattack events, as Haase et al [83] noted that adverse events during an attack are typically reported through the same system that the attack has disabled. Choudhuri et al [66] highlighted this further in their review of cyberattack downtime affecting ICUs in India, where they note that systematic data on near misses and patient-level outcomes during the downtime period were unavailable, creating a significant surveillance gap. Social media data therefore serve not merely as a supplementary data source but as a mechanism for circumventing the structural barriers to harm reporting that cyberattacks themselves create [23-26,83]. Staff appeared more forthcoming on social media, reporting adverse prescribing events (*r/nursing*: *“*already heard of two very serious related med errors*”*) and severe patient harms and deaths (*“*Saw a dude die and couldn’t get ahold of doctors*”*). These findings demonstrate the value of complementing traditional systematic academic reviews with social media narratives in order to gain deeper insights into the landscape of harms emerging during these incidents.

### Strengths and Limitations

This systematic review has several strengths. To our knowledge, it is the first systematic, clinically focused synthesis of patient-level harms during health care cyberattacks, drawing on 59 empirical case studies spanning 13 countries, diverse health care systems, attack types, and clinical settings. The dual-source methodology that combines peer-reviewed academic literature with social media data from 3408 health care subreddits is a novel contribution that captures patient and staff experiences that are systematically underrepresented in formal reporting channels. The resulting open-source CIPHER dataset published alongside the manuscript provides a reusable, categorized resource for future research and preparedness planning [31].

Several limitations must also be acknowledged. Although methodological quality was generally high according to MMAT appraisal, the evidence base remains heterogeneous, comprising predominantly qualitative case studies, descriptive reports, observational analyses, and service evaluations. Consequently, the recurring harms identified in this review should be interpreted as consistent patterns across diverse health care settings, rather than estimates of their frequency or magnitude. Nevertheless, the convergence of similar harms across multiple nations and study designs strengthens confidence that these vulnerabilities are widespread rather than isolated events.

Furthermore, this synthesis is limited to English-language peer-reviewed publications, risking geographic underrepresentation from non-Anglophone health care systems. Gray literature databases were not systematically searched, no citation searching or author contacts were undertaken to identify additional studies, and the search was not formally peer-reviewed by an information specialist, although it was developed in full accordance with Cochrane guidance and Methodological Expectations of Cochrane Intervention Reviews (MECIR) standards. The narrative synthesis was based on descriptive frequency counts of reported harms, rather than quantitative effect estimates, and specialty groupings were determined retrospectively following data extraction rather than being pre-specified. As a result, the synthesis cannot estimate the magnitude, probability, or clinical significance of harm within a given specialty.

Finally, while rich in patient voices, Reddit data have previously been identified to be biased toward male experience and particular age groups [37]. This research finds that patients affected by ransomware are often those who are older, with complex chronic conditions, and who endurewho suffer from the loss of the EHR. Yet, older adult patients, and particularly those who are not digital natives, may be less vocal on the platforms we have analyzed. Finally, underreporting bias is an inherent risk in cyberattack research, as adverse events are typically documented through the same systems disabled by the attack, meaning that the true scale of patient harm is likely greater than that which the published evidence reflects.

### Conclusion

Health care cyberattacks are no longer a theoretical risk to patient safety; instead, they are a recurring, measurable cause of patient harm across diverse clinical settings, specialties, and countries. This review provides the first systematic, clinically focused synthesis of those harms, uniquely integrating published case studies with patient and staff narratives to move beyond the organizational and technical framing that has dominated health care cybersecurity research. The resulting CIPHER dataset, published openly alongside this manuscript, links technical failure domains to downstream clinical risks across care settings, providing a reusable resource for future research, policy development, and institutional preparedness planning [31]. Our framework enables health systems to identify high-risk services, prioritize downstream planning, and embed patient safety metrics within cyber-resilience strategies. We encourage researchers to access this dataset and investigate subcategories of harms that were out of the scope of this paper, such as harms more likely to affect specific patient groups (eg, the older adult vs pediatric populations), the impact on health equity (risk to specific demographic groups), and specialty-specific impacts (eg, psychiatry and obstetrics).

This research demonstrates that cyberattack preparedness must be understood as a patient safety imperative, rather than an IT governance issue. The evidence presented here illustrates that the patients most harmed are not always those most visible in traditional safety metrics. Patients requiring time-critical care are at acute risk, but those whose harm accumulates through the intersection of digital dependence and clinical vulnerability represent an underrecognized and underprotected group that response frameworks must explicitly address.

Future research should build on this foundation by examining subcategories of harm across specific demographic groups, investigating population health and health equity implications of cyberattack-induced care disruption, and developing validated metrics for detecting cyberattack-induced patient harm in real time. Integrating social media data into cybersecurity monitoring frameworks represents a promising approach for capturing harms that formal reporting systems miss. As cyberattacks on health care organizations continue to rise in frequency and severity, the boundary between cybersecurity and patient safety has ceased to exist, demanding unified frameworks that treat digital resilience and clinical preparedness as two expressions of the same obligation.

## Supplementary material

10.2196/88257 Multimedia Appendix 1 Search strategies for systematic review across 5 databases (January 1, 2004-July 1, 2026): search queries, vocabulary type (free text and MeSH/Emtree terms), database access links, and records retrieved.

10.2196/88257 Multimedia Appendix 2 Summary of the included studies (n=59). Included studies are summarized by country, study type, cyberattack details, and the principal reported patient impacts. The complete study-level data extraction table, including all extracted variables, is provided in Multimedia Appendix 3.

10.2196/88257 Multimedia Appendix 3 Full data extraction table.

10.2196/88257 Multimedia Appendix 4 Extracts from the full Cyberattack Impacts, Patient Harms, and Emergency Response (CIPHER) database with examples of identified patient-level harms from the academic literature (column 1) and the social media data (column 2), categorized by clinical domains (numbered as Sections for reference throughout the text). Quotes are provided from the authors of the academic paper alongside the respective reference, or from the social media post alongside the respective subreddit. Social media data posts are deidentified (references to usernames are removed) and any personal details or reference to specific providers/vendors are replaced with asterisks; quotes are numbered (eg. Q1.1) for reference throughout the text.

10.2196/88257 Multimedia Appendix 5 Insights from social media posts regarding cyberattack impacts across wider health care infrastructure, outside the hospital, and in the community.

10.2196/88257 Checklist 1 PRISMA checklist.

10.2196/88257 Checklist 2 PRISMA-S checklist.

10.2196/88257 Checklist 3 SWiM Reporting Checklist: Synthesis Without Meta-analysis (SWiM) items: SWiM is intended to complement and be used as an extension to PRISMA, taken from the original SWiM methods article.

## Abbreviations

CCTV: closed-circuit television
CDC: Centers for Disease Control and Prevention
CIPHER: Cyberattack Impacts, Patient Harms, & Emergency Response
CT: computed tomography
EBRT: external beam radiotherapy
ED: emergency department
EMR: electronic medical record
FDA: Food and Drug Administration
HDO: health care delivery organization
ICU: intensive care unit
IoMT: internet of medical things
MECIR: Methodological Expectations of Cochrane Intervention Reviews
MMAT: Mixed Methods Appraisal Tool
MQSA: Mammography Quality and Standards Act
MRI: magnetic resonance imaging
OHCA: out-of-hospital cardiac arrest
PICO: Population, Intervention, Comparator, and Outcome
PRISMA: Preferred Reporting Items for Systematic Reviews and Meta-Analyses
PRISMA-S: Preferred Reporting Items for Systematic Reviews and Meta-Analyses–Search
QA: quality assurance
SWiM: Synthesis Without Meta-analysis
TCP: tumor control probability
