# Correction: Unveiling Patient-Level Harms and At-Risk Clinical Groups During Hospital Cyberattacks: Systematic Review of Global Case Studies and Social Media Data

**DOI:** 10.2196/114623

**Published:** 2026-11-04

**Authors:** Isabel Straw, Deepak Kumar, Seoyoung Kweon, Jordan Selzer, Christian Dameff, Jeffrey Tully

**Affiliations:** 1 Department of Computer Science and Engineering, University of California San Diego, 9500 Gilman Dr, San Diego, CA, 92093, United States, 1 858-534-2230; 2 Department of Emergency Medicine, George Washington University, Washington, DC, United States; 3 Department of Emergency Medicine, University of California San Diego, San Diego, CA, United States; 4 Department of Anesthesia, University of California San Diego, San Diego, CA, United States

 In “Unveiling Patient-Level Harms and At-Risk Clinical Groups During Hospital Cyberattacks: Systematic Review of Global Case Studies and Social Media Data” [1], the authors noted one error.

The file previously attached as Multimedia Appendix 4 was a duplicate of Multimedia Appendix 5. This has been replaced with the proper file (included here as Multimedia Appendix 1).

The correction will appear in the online version of the paper on the JMIR Publications website together with the publication of this correction notice. Because this was made after submission to PubMed, PubMed Central, and other full-text repositories, the corrected article has also been resubmitted to those repositories.

## Supplementary material

10.2196/114623 Multimedia Appendix 1 Corrected file for Multimedia Appendix 4: Extracts from the full Cyberattack Impacts, Patient Harms, and Emergency Response (CIPHER) database with examples of identified patient-level harms from the academic literature (column 1) and the social media data (column 2), categorized by clinical domains (numbered as Sections for reference throughout the text). Quotes are provided from the authors of the academic paper alongside the respective reference, or from the social media post alongside the respective subreddit. Social media data posts are deidentified (references to usernames are removed) and any personal details or reference to specific providers/vendors are replaced with asterisks; quotes are numbered (eg. Q1.1) for reference throughout the text.
