# Supplementary material for: Unveiling Patient-Level Harms and At-Risk Clinical Groups During Hospital Cyberattacks: Systematic Review of Global Case Studies and Social Media Data
Source: J Med Internet Res. 2026 Sep 28;28:e88257. doi: 10.2196/88257 (PMC13622069; doi:10.2196/88257)
Supplement: Supplementary file 2 [file jmir-v28-e88257-s001.docx]

**Appendix 1-** **Search strategies for systematic review across five databases (1 January 2004 – 1 July 2026): search queries, vocabulary type (free text and MeSH/Emtree terms), database access links, and records retrieved.**

Searches were executed on 1 July 2026 across all five databases. PubMed supports precise date-level filtering and was limited accordingly (1 January 2004 – 1 July 2026). Scopus, Web of Science, MEDLINE, and Embase support only year-level date filtering; the calendar-year upper bound (2026) was applied at the search-string level, with the search execution date serving as the practical cutoff. Following retrieval, results were filtered to peer-reviewed journal articles using each platform's native document-type limiter (excluding conference abstracts, retracted articles, books, and notes), applied uniformly across all five databases prior to deduplication. Emtree mapping for 'computer security' confirmed via Ovid Map Term function, as equivalent to the MEDLINE MeSH heading of the same name.

| **Database & Access URL** | **Vocabulary** | **Full Search Strategy with MESH Terms and Key Terms** | **Records Retrieved** |
| --- | --- | --- | --- |
| **Scopus**  [scopus.com> Advanced Search](https://www.scopus.com/search/form.uri#advanced) | *Title/Abstract free-text only (as there are no subject headings on this platform)* | TITLE-ABS ( "cyberattack*"  OR "cyber attack*"  OR "cyber incident*"  OR "cyber threat*"  OR "cybersecurity incident*"  OR "security breach*"  OR "cyber breach*"  OR "cybersecurity breach*"  OR "data breach*"  OR "ransomware"  OR "malware"  OR "denial of service"  OR "ddos"  OR "ddos attack"  OR "network attack*" )  AND  TITLE-ABS ( "hospital*"  OR "healthcare"  OR "health care"  OR "health service*"  OR "healthcare service*"  OR "patient care"  OR "clinical care"  OR "medical center*"  OR "medical centre*"  OR "health system*"  OR "healthcare organisation"  OR "medical facility*" )  AND PUBYEAR > 2003 AND PUBYEAR < 2027  AND LANGUAGE(english) | 4585 |
| **Web of Science**  [webofscience.com> Advanced Search](https://www.webofscience.com/wos/woscc/advanced-search) | *Title/Abstract free-text only (as no subject headings on this platform)* | (  TI=(  "cyberattack*"  OR "cyber attack*"  OR "cyber incident*"  OR "cyber threat*"  OR "cybersecurity incident*"  OR "security breach*"  OR "cyber breach*"  OR "cybersecurity breach*"  OR "data breach*"  OR "ransomware"  OR "malware"  OR "denial of service"  OR "ddos"  OR "ddos attack"  OR "network attack*"  )  OR  AB=(  "cyberattack*"  OR "cyber attack*"  OR "cyber incident*"  OR "cyber threat*"  OR "cybersecurity incident*"  OR "security breach*"  OR "cyber breach*"  OR "cybersecurity breach*"  OR "data breach*"  OR "ransomware"  OR "malware"  OR "denial of service"  OR "ddos"  OR "ddos attack"  OR "network attack*"  )  )  AND  (  TI=(  "hospital*"  OR "healthcare"  OR "health care"  OR "health service*"  OR "healthcare service*"  OR "patient care"  OR "clinical care"  OR "medical center*"  OR "medical centre*"  OR "health system*"  OR "healthcare organi?ation*"  OR "medical facility*"  )  OR  AB=(  "hospital*"  OR "healthcare"  OR "health care"  OR "health service*"  OR "healthcare service*"  OR "patient care"  OR "clinical care"  OR "medical center*"  OR "medical centre*"  OR "health system*"  OR "healthcare organi?ation*"  OR "medical facility*"  )  )  AND PY=(2004-2026) | 2571 |
| **PubMed**  <https://pubmed.ncbi.nlm.nih.gov/advanced/> | *Free-text(Title/Abstract) and MeSH Terms("Computer Security”)* | (  (  cyberattack*[Title/Abstract]  OR "cyber attack*"[Title/Abstract]  OR "cyber incident*"[Title/Abstract]  OR "cyber threat*"[Title/Abstract]  OR "cybersecurity incident*"[Title/Abstract]  OR "security breach*"[Title/Abstract]  OR "cyber breach*"[Title/Abstract]  OR "cybersecurity breach*"[Title/Abstract]  OR "data breach*"[Title/Abstract]  OR ransomware[Title/Abstract]  OR malware[Title/Abstract]  OR "denial of service"[Title/Abstract]  OR DDoS[Title/Abstract]  OR "DDoS attack"[Title/Abstract]  OR "network attack*"[Title/Abstract]  )  OR  "Computer Security"[Mesh]  )  AND  (  (  hospital*[Title/Abstract]  OR healthcare[Title/Abstract]  OR "health care"[Title/Abstract]  OR "health service*"[Title/Abstract]  OR "healthcare service*"[Title/Abstract]  OR "patient care"[Title/Abstract]  OR "clinical care"[Title/Abstract]  OR "medical center*"[Title/Abstract]  OR "medical centre*"[Title/Abstract]  OR "health system*"[Title/Abstract]  OR "healthcare organization*"[Title/Abstract]  OR "healthcare organisation*"[Title/Abstract]  OR "medical facility*"[Title/Abstract]  )  OR  "Hospitals"[Mesh]  OR "Health Facilities"[Mesh]  OR "Delivery of Health Care"[Mesh]  )  AND  ("2004/01/01"[Date - Publication] : "2026/07/01"[Date - Publication])  AND English[Language]  AND Humans[Mesh] | 3118 |
| **MEDLINE (via Ovid)**  <https://ovidsp.ovid.com/> | *Free-text (ti,ab)+ MeSH (e.g. Computer Security, Hospitals/)* | 1. (  cyberattack*  OR "cyber attack*"  OR "cyber incident*"  OR "cyber threat*"  OR "cybersecurity incident*"  OR "security breach*"  OR "cyber breach*"  OR "cybersecurity breach*"  OR "data breach*"  OR ransomware  OR malware  OR "denial of service"  OR DDoS  OR "DDoS attack"  OR "network attack*"  ).ti,ab.  2. Computer Security/  3. 1 OR 2  4. (  hospital*  OR healthcare  OR "health care"  OR "health service*"  OR "healthcare service*"  OR "patient care"  OR "clinical care"  OR "medical center*"  OR "medical centre*"  OR "health system*"  OR "healthcare organization*"  OR "healthcare organisation*"  OR "medical facility*"  ).ti,ab.  5. Hospitals/  OR Health Facilities/  OR Delivery of Health Care/  6. 4 OR 5  7. 3 AND 6  8. Limit 7 to (English language and Humans and yr="2004 -2026") | 2928 |
| **EMBASE (via Ovid)**  <https://ovidsp.ovid.com/> | *Free-text (ti,ab)+ Emtree (Computer Security, Hospitals/)* | 1. (  cyberattack*  OR "cyber attack*"  OR "cyber incident*"  OR "cyber threat*"  OR "cybersecurity incident*"  OR "security breach*"  OR "cyber breach*"  OR "cybersecurity breach*"  OR "data breach*"  OR ransomware  OR malware  OR "denial of service"  OR DDoS  OR "DDoS attack"  OR "network attack*"  ).ti,ab.  2. Computer Security/  3. 1 OR 2  4. (  hospital*  OR healthcare  OR "health care"  OR "health service*"  OR "healthcare service*"  OR "patient care"  OR "clinical care"  OR "medical center*"  OR "medical centre*"  OR "health system*"  OR "healthcare organization*"  OR "healthcare organisation*"  OR "medical facility*"  ).ti,ab.  5. Hospital/  OR Health Care Facility/  OR Health Care Delivery/  6. 4 OR 5  7. 3 AND 6  8. Limit 7 to (English language and Humans and yr="2004-2026") | 3627 |
| **Total records retrieved** | | | 16,829 |
| **Number of Duplicates Deleted** | | | 5359 |
| **Total Abstracts Screened** | | | 11,470 |
| **Total Studies Included** | | | 59 |
