# Supplementary material for: Unveiling Patient-Level Harms and At-Risk Clinical Groups During Hospital Cyberattacks: Systematic Review of Global Case Studies and Social Media Data
Source: J Med Internet Res. 2026 Sep 28;28:e88257. doi: 10.2196/88257 (PMC13622069; doi:10.2196/88257)
Supplement: Supplementary file 3 [file jmir-v28-e88257-s002.docx]

**Table S1: A Summary of the included studies (n = 59). Included studies are summarised by country, study type, cyberattack details, and the principal reported patient impacts. The complete study-level data extraction table, including all extracted variables, is provided in Multimedia Appendix 3.**

| **Reference** | **Study type** | **Cyberattack details & location of affected organisation** | **Principal patient impacts**  **(further detailed in Appendix 3).** |
| --- | --- | --- | --- |
| Moore et al [68] | Multisite qualitative focus-group study, followed by thematic analysis | Location: Ireland Details: May 2021 Conti ransomware attack that disrupted patient-information, clinical care and laboratory systems across Ireland’s Health Service Executive (HSE). | Radiotherapy was interrupted or delayed, diagnostic and laboratory processes slowed, and care was cancelled or deferred. Acute and maternity patients were particularly affected because these services relied heavily on electronic systems. |
| Ghafur et al [53] | Retrospective quantitative observational study | Location: UK Details: The May 2017 WannaCry ransomware attack disrupted electronic records, prescribing, imaging and other digital systems across the UK NHS; more than 600 organisations were affected, including 34 infected hospital trusts. | Outpatient appointments were cancelled, admissions and service activity fell, and emergency departments diverted patients to neighbouring hospitals. Infected trusts recorded approximately 50% more outpatient cancellations and reductions in elective, emergency and total admissions. |
| Stowman et al [73] | Single-centre anatomic pathology case study. | Location: USA  Details: In 2020 a malicious email injected a piece of malware into a device that - once reconnected to the organisation network - facilitated the infected device to launch a variety of attacks on the network. The ransomware took out the primary networking and computing infrastructure of the hospital system. | Clinical outcomes were not directly quantified, instead the paper reported service disruptions that had downstream effects on patient care. Radiology was unable to provide normal imaging support, laboratory results were delayed, oncology patients had to be referred elsewhere and surgical activity required strict triage. |
| Stowman et al [81] | Single-centre anatomic pathology downtime case study. | Location: USA Details: A 2020 System-wide ransomware that resulted in an immediate shutdown of all hospital IT, including the electronic health record, radiology, pharmacy, scheduling, billing, email and networked equipment. There was loss of digital tracking, reporting, access to prior results and clinical information. | The researchers did not measure clinical outcome metrics, but reported diverse care-delivery impacts with patient safety implications: increased turnaround times, reduced in-house processing capacity, inability to access previous pathology or clinical data and high-risk manual identification. |
| Goodwin et al [80] | Single-centre clinical pathology operations case study. | Location: USA Details: A 2020 ransomware-related shutdown of hospital systems, that resulted in loss of the electronic health record, middleware, electronic ordering, pneumatic tube transport, courier badge access, digital phones/faxes, paging, shared drives and instrument connectivity. | No direct clinical outcome measures, but major and safety impacts with patient-care implications. This included substantial turnaround-time prolongation, reduced access to results for care teams, missed or unlocatable results and loss of cumulative data and past health record information. |
| Perry et al [72] | Single-centre breast-imaging service recovery case study. | Location: USA  Details: Following a phishing attack perpetrated via email, a Trojan malware infected a UVMMC employee laptop while it was off the network. When this laptop was reconnected to the network, the malware, and subsequently ransomware, was able to infiltrate the virtual computer server environment, encrypting all virtual hard disks. | Significant impact on patient access to services, with most screening and routine diagnostics being deferred, many patients being turned away from appointments, and patients being advised to seek imaging at other institutions. Screening mammograms could not be reported within the mandated 30-day time frame, requiring regulatory notification and diagnostic interpretation was constrained by lack of prior imaging and clinical data. |
| Klatt et al [82] | Longitudinal organisational risk-management case analysis. | Location: USA  Details: Multiple cyberattack incidents involving malware and ransomware-related data breaches, with both internal and external threat vectors. The research maps key vulnerability domains, highlighting how compromise of the network enables enterprise-wide system control and attack susceptibility. | No direct clinical outcomes measures, instead patient impact is conceptualised as loss of confidentiality of health information, increased long-term exposure to fraud and systemic service risk due to organisational disruption which may impede patient care. |
| Haase et al [83] | Single site, practice-based descriptive case study. | Location: USA Details: Suspected ransomware in 2020 leading to a shutdown of all clinical computer systems, resulting in complete loss of the electronic health record, computerised order systems, medication administration record systems and clinical decision support systems. | Authors highlighted the increased risk of medication administration errors due to loss of the barcode medication system, overrides were required creating risks of wrong drug, wrong doses and adverse events. Staff highlighted particular risks with loss of the heparin protocol, where reliance on newer automated systems may lead to risks in anticoagulation management. |
| Fink and Gresko [84] | Descriptive report outlining the operational and clinical consequences of the Change Healthcare Ransomware attack | Location: USA Details: In 2024 BlackCat/ALPHV allegedly used compromised credentials to gain access to CHC’s system, which lacked multifactor authentication, and deployed the ransomware while stealing data. This led to the immediate disconnection of systems to contain the spread, preventing hospitals and pharmacies from processing claims. | There was immediate loss of medication access pathways, patients were unable to obtain medicines unless paying out-of-pocket, and there was long-term potential harm through confidentiality breach and fraud exposure. |
| Powell et al [85] | Single-centre infection-control continuity service report. | Location: Ireland  Details: The 2021 national ransomware attack encrypted files and disabled user access, leading to a complete shutdown of the HSE IT Infrastructure. There was a loss of telecommunications (including voice over internet protocol), access to data bases and access to clinical and support services. | Delayed notification of notifiable diseases increased risks relating to outbreaks, point-prevalence screening also had to cease reducing the hospitals ability to detect transmission. The loss of electronic alerts meant isolation and cohorting was curtailed, and the lack of COVID-19 results meant a higher risk of inappropriate patient placement and exposure. |
| S Devi [75] | Narrative analysis of healthcare cyberattacks and patient harm. | Location: USA Details: Predominantly ransomware attacks using malware to disrupt, damage or deny access to computer systems, before demanding payments from healthcare organisations. | References the case of a German patient who died from an aortic aneurysm when her treatment was delayed due to a cyberattack that disrupted her care. Looks specifically at disruption to cancer care operations, reflecting on instances of patient documents and images being leaked into the dark web. |
| Pontier et al [47] | Single-centre case report. | Location: France  Details: In 2021 a ransomware attack known as 'cryptolocker' occurred at 2am, where the timing was thought to be a tactical choice, given that less IT staff were available at night. Entry point was unclear and not thought to be via email, but instead via a vulnerability in the hospital firewall (Fortinet). | Clinical impacts on cancer patients were most apparent, with radiotherapy having to stop immediately, preventing treatment for an unclear duration. Patients were referred to nearby centres, with potential impacts of treatment delay and cancer progression. |
| Cassim et al [50] | National laboratory network experiential impact report. | Location: South Africa  Details: A 2024 ransomware attack named 'Blacksuit' that encrypted servers and applications, causing a complete shutdown of IT operations, including internet, intranet, the 'TrakCare' laboratory information system and the central data warehouse containing decades of historical results. | Authors detailed how clinician's ability to appropriately diagnose, manage and monitor their patients was directly affected by the immediate doubling of turnaround times for blood tests. Testing capacity was reduced to urgent investigations only and previous laboratory history was unavailable, creating long-term implications for continuity of care and disease surveillance. |
| Keogh et al [48] | Mixed-methods cancer-centre impact evaluation. | Location: Ireland  Details: The 2021 Conti ransomware led to all hospital IT systems being shut down. Affected systems included email and patient administration systems, core cancer service platforms and diagnostics, radiology services (RIS/PACS), radiotherapy oncology information systems, lab middleware/LIMS connectivity and clinical management systems. | The cessation of cancer services had immediate impacts on patients, with the cancellation of radiotherapy treatments and tumour surgeries. Diagnostics were severely constrained (~90% drop in haematology and radiology capacity, ~75% drop for biochemistry), immediately impacting patient diagnosis and monitoring. Outpatient activity also fell by 50%, reducing care of patients in the community. |
| Santalo et al [86] | Single-centre pharmacy downtime operations report. | Location: USA  Details: The 2021 cyberattack caused the hospital to simultaneously lose access to the electronic health record, admitting and registration systems, pharmacy information systems, barcode medication administration, automated dispensing cabinet servers, inventory management and compliance software. | The researchers describe how the disruptions to the pharmacy workflow and loss of automation can be manually intensive and unsafe for patients. Loss of barcode administration and clinical decision support can remove automated allergy checks, interaction screening and electronic verification. The increased turnaround times for processing can also lead to delays in treatment. |
| Van boven et al [11] | Multihospital qualitative interview study of ransomware response. | Location: USA Details: The ransomware incidents involved a loss of access to digital radiology and laboratory systems and a loss of EHR access. Further systems impacted including: Computers, internal internet access, internal telephone/email and digital medical systems. | Staff described conditions that could potentiate patient harms via delays, missing clinical histories and follow-up gaps. Delays were described as massive and clinicians raised concerns about delays to patient care and lost follow up. |
| Harrison et al [71] | Multisite radiation-oncology technical incident report. | Location: USA  Details: 2021 ransomware attack on the major RVS vendor, which forced an immediate shutdown of the system and removed the department's ability to verify dose delivery and treat patients. The RVS was unavailable for 2.5 weeks, with full functionality not returning until after 4.5 weeks. | All treatments were initially stopped, with no patients being treated in the first 24 hours. Given that these cancer therapies are time-critical treatments, the associated treatment gaps for patients could affect oncological complications, outcomes and progression. |
| Nelson et al [57] | Health-network radiation-oncology response report. | Location: USA Details: The 2020 cyberattack forced a shutdown of the hospital network. The UVMHN experienced multiple clinical system outages as a result of the cyberattack. | All treatments were cancelled on day 1 and clinicians had to triage patients, due to the risk of extended treatment breaks affecting oncological outcomes. High priority patients were transferred to affiliate centres or treated using simplified techniques, while others experienced planned delays. |
| Zhao et al [77] | Mixed-methods trauma-registry, survey and interview study. | Location: USA Details: The 2017 SamSam ransomware forced the immediate loss of the electronic medical record, imaging system (PACS), e-prescribing, scheduling systems, communication tools and internet access. The transition occurred without the structured safety process that historically supported paper systems. | Measured trauma outcomes (volume, severity, mortality) were not statistically different from the comparator year. However, qualitative and workflow data demonstrated multiple mechanisms of patient safety risk: the loss of EMR checks and balances, inability to review imaging or laboratory results, disorganised paper charge and reduced pre-operative case preparation created key points of vulnerability where mistakes may occur.. |
| Gourd [87] | Expert analysis of cyberattacks affecting oncology services. | Location: USA  Details: Ransomware attack on a 3rd party cloud platform hosting radiotherapy linear-accelerator software and associated oncology data. Disruption extended beyond data breach to loss of functionality of treatment-delivery systems, causing cancellation or delay of radiotherapy sessions across 42 sites. | The cyberattack directly interrupted radiotherapy delivery, preventing treatment of patients. Furthermore, loss of access to referrals, imaging, and diagnostic workflows were linked to slower cancer screening and staging. |
| Zhao et al [88] | Single-centre grounded-theory interview study of surgical care. | Location: USA Details: The cyberattack resulted in the complete loss of the EHR, requiring reversion to paper charts. Diverse results and order sets were impacted including laboratory and imaging results, medication lists, consultation requests, and prescribing workflows were no longer accessible. | The loss of the EHR disrupted routine care and introduced new risks to patients, especially where regular processes took longer to complete and paper prescribing led to delayed medication access as community pharmacies refused paper scripts. Clinical information was harder to access and less reliable because staff had to rely on paper flows, staff reported that results were often misplaced or put in the wrong chart, raising the risk of medical error. |
| Leonard et al [89] | Single-centre paediatric emergency-department analysis. | Location: Ireland Details: The 2021 ransomware attack resulted in the shutdown of core digital infrastructure, resulting in the loss of patient administration systems, emergency department information system (EDIS), laboratory and radiology access and all intranet/internet connectivity. | Median length of stay fell and critically ill patients moved more quickly from registration to triage, and from clinician review to disposition, largely because documentation requirements were reduced. However, this occurred alongside a rise in data imperfections, missing discharge destinations and inaccurate timestamp documentation. |
| Strong et al [90] | Reflective practice report from radiology department. | Location: Canada Details: The 2021 cyberattack caused the hospital IT systems to become inaccessible, removing normal digital ordering, reporting and integrated workflow support. Departments reverted to fully paper-based imaging requests and manual work lists. | The loss of automated worklists and electronic identifiers created a high risk of misidentification. For patients, imaging remained available but at a reduced and prioritised capacity, meaning that non-urgent examinations were deferred, slowing diagnostic and treatment pathways across the system. |
| Hoffman et al [49] | Regional orthopaedic ethics and service-delivery analysis. | Location: New Zealand Details: In 2021 a major ransomware attack hit the Waikato District Health Board in New Zealand, disabling all computer systems, the internet, printers and hospital phones for close to 4 weeks. The patient management and radiology systems used by the hospital were completely out of operation for 24 days, while the hospital intranet, key for internal communications and dissemination of essential staff information, was offline for 2 months. | Patients experienced diagnostic and treatment delays due to reduced capacity for laboratory and imaging tests - CT Capacity fell to two scans per hour. Elective surgery was widely deferred, creating risks of lasting morbidity through disease progression and prolonged pain and disability. |
| Lacobucci [64] | Real-time health-service disruption report. | Location: UK Details: The 2024 Synnovis ransomware attack disabled the pathology IT service 'Syynovis', which formed a vital part of NHS infrastructure, and was usually used to process laboratory samples and match patient blood types. The hospitals declared critical incidents and emergency work was prioritised, with transition to manual processes. | Patient harms were driven by the loss of timely pathology results and transfusion resources. Hospitals were unable to match patient blood type at the same frequency as normal, leading to postponed operations, diversion of patients and a national appeal for group O blood so that emergency care could continue. |
| Aljaidi et al [91] | Quantitative descriptive ‘before-during-after’ analysis of hospital activity. | Location: UK Details: In 2017 a self-propagating ransomware worm, exploiting the SMBv1 vulnerability (MS17-010) via EternalBlue, spread across national health networks. The encryption of endpoints and risk-mitigation shutdowns meant that clinical IT, email, scheduling systems, radiology and some network-connected medical devices became unavailable, forcing a shift to paper-based workflows and service disruption. | At affected trusts there were marked reductions in delivered care, with 6% fewer admissions, 4% fewer ED attendances and 9% fewer elective admissions, alongside widespread cancellation of outpatient appointments and ambulance diversions. |
| Dameff et al [59] | Regional emergency-care registry study with pre/during/post comparison. | Location: USA Details: A 2021 ransomware attack caused a month-long loss of core digital infrastructure at the target hospital, including loss of the electronic health record and imaging platforms, forcing a shift to paper-based and manual processes. | At neighbouring hospitals, patients experienced measurable access and timeliness harms, with ED attendances rising by 15%, ambulance arrivals rising by 35% and waiting room time increasing by 48%. Median length of stay for admitted patients increased by ~34%, while the proportion that left without being seen more than doubled and departures against medical advice increased, indicating a loss of definitive assessment and treatment. |
| Abouk et al [60] | Difference-in-differences analysis of targeted and neighbouring hospitals. | Location: USA Details: The study analysed ransomware incidents that disrupted care delivery (as identified in previous cybersecurity datasets), where targeted hospitals experienced operational impairment that affected clinical workflows. Technical failures were not described in detail, but attacks were characterised by loss of digital functionality and service capacity. | Victim hospitals experienced short-term reductions in care delivery, with ED visits falling by 8.1% in the first week and 16.2% in the second week after the attack. In contrast, nearby hospitals saw a compensatory increase in ED visits (peaking at 7% by week 3), without a corresponding rise in admissions, demonstrating redistribution of acute demand across the local health system. |
| Pham et al [55] | Regional cardiac-arrest registry and interrupted time-series study. | Location: USA Details: In 2021, a month-long ransomware attack caused complete loss of the EHR and core digital systems at a four-hospital organisation, triggering regional ambulance diversion and sustained increases in ED and inpatient census at adjacent hospitals. | At the neighbouring hospitals the cyberattack was associated with a marked increase in cardiac arrest caseload and substantially worse patient outcomes. Survival with favourable neurological outcomes dropped from 38.1% to 5.3% (p<0.001), with the most pronounced deterioration in out-of-hospital cardiac arrest (40.0% to 4.5%, p=0.02). |
| Chen et al [92] | Single-centre radiology recovery-planning case study. | Location: USA Details: A 2020 ransomware attack rendered hospital digital infrastructure unusable, including EMR, PACS, RIS, diagnostic workstations, scheduling systems, paging directories, and hospital intranet. Some modalities could still acquire images but without network transmission, forcing at-modality interpretation on non-diagnostic monitors, local CD/DVD image transfer, paper ordering, manual patient identifiers and accession conventions, and telephone/fax result communication. | Patient access to imaging was immediately reduced because elective studies were postponed, some sites stopped scanning, and urgent examinations were consolidated to locations with on-site radiologists. Image interpretation at the scanner using non-diagnostic displays and loss of teleradiology slowed diagnosis and increased the risk of communication failure for critical findings. |
| Zhao et al [101] | Single-centre grounded-theory study of surgical communication. | Location: USA Details: The cyberattack resulted in a complete shutdown of the electronic health record, eliminating electronic documentation, order entry, and asynchronous messaging. | Patient care became more bedside-centred during the attack, with clinicians relying on repeated face-to-face discussions to clarify plans, confirm orders, and hand over information. This increased explicit, deliberate communication and improved shared understanding of care plans. |
| Duffy et al [93] | National cross-sectional survey of hospital laboratories. | Location: Ireland Details: The 2021 ransomware attack caused shutdown of the national healthcare network, with loss of laboratory systems, digital requesting and reporting, email, and in some sites telecommunications. Over half of laboratories had no functioning LIS for prolonged periods, requiring full manual workflows for specimen reception, tracking, analysis, and reporting, with loss of automated reference ranges, alert flags, historical results, and electronic transmission of results. | Diagnostic activity decreased markedly, with most laboratories reporting substantial reductions in test requests, particularly from primary care, suggesting deferred or missed investigations. Manual processing and transcription increased workload, turnaround times, and risk of identification and reporting errors, while lack of previous results and decision-support functions reduced the clinical reliability of testing for patient management. |
| Stowman and Kalof [94] | Single-centre qualitative anatomic pathology recovery case study. | Location: USA Details: The 2020 cyberattack resulted in complete loss of the EHR, laboratory information system, internet and intranet, email, dictation software, printers, PACS, scheduling systems, billing services, and networked telephony. Specimen accessioning, tracking, slide printing, reporting, and access to prior patient results were all disabled, forcing a fully manual workflow and necessitating diversion of large specimen volumes to external laboratories. | Inability to accession and track specimens created a growing backlog and risk to specimen integrity and result accuracy, and absence of prior records limited diagnostic context. Urgent results had to be communicated by telephone, delaying definitive reporting and treatment planning. |
| Tarka et al [95] | Mixed-methods orthopaedic cohort and workflow evaluation. | Location: USA Details: The 2020 ransomware attack destroyed the core network, disabling over 5000 computers and causing complete loss of the EMR, imaging access, electronic scheduling, order entry, intranet/internet, phones, paging, and centralised patient-tracking systems. | The departmental operative capacity fell by ~53%, restricting activity largely to urgent cases and delaying elective surgery. Core care processes became manual and fragmented: patient location tracking, medication reconciliation, laboratory testing, and order execution were slow and inconsistent, and intraoperative decisions were made without real-time imaging. |
| Filipec and Plasil [46] | Socio-technical organisational case study with incident reconstruction. | Location: Czechia  Details: In 2019 a multi stage ransomware intrusion was delivered via a phishing campaign, with Emotet enabling lateral movement, TrickBot harvesting credentials and escalating privileges, and Ryuk performing network-wide encryption. Core clinical and administrative systems became inoperable, including the hospital information system, domain services, email, laboratory and prescribing platforms, and most networked workstations. | Authors report that the hospital was effectively non-functional for approximately one week, with emergency services diverted and routine care cancelled or postponed. Diagnostic capacity, laboratory processing, electronic prescribing, and blood bank functionality were temporarily unavailable, requiring external procurement of blood products and reliance on manual communication of urgent results, with significant implications for patient management. |
| Slattery et al [95] | Single-centre comparative study of outpatient non-attendance. | Location: Ireland  Site: The 2021 cyberattack disabled core health IT infrastructure, including patient administration and automated SMS appointment reminder systems. Although the study focuses on one department, the disruption was system-wide and also affected laboratory, radiology, and scheduling platforms, increasing administrative burden and removing digital communication tools used to coordinate outpatient care. | Loss of the reminder system was associated with a statistically significant rise in non-attendance from 13.0% pre-attack to 16.1% post-attack (p = 0.002), equating to 32 additional missed appointments in two weeks that required rebooking into already over-subscribed clinics. These missed visits risk delayed diagnosis and treatment in a specialty where timely in-person assessment is essential and waiting lists are prolonged. |
| Oliver et al [45] | Single-centre quantitative radiotherapy operations case study. | Location: Canada  Details: A 2019 zero day malware infection forced shutdown of hospital networks and encrypted multiple clinical systems, including the oncology information system (OIS) and hospital IT system. Network isolation also severed connectivity to the satellite centre. | The radiotherapy delivery was at risk of clinically unsafe delays; treatment gaps beyond accepted thresholds can reduce cure rates in several cancers. Although no fractionation errors occurred, the event exposed major safety vulnerabilities: uncertainty about patient treatment status, dependence on a single digital record-and-verify system, and lack of predefined downtime procedures. |
| Harvey et al [74] | Multicentre cancer-trials survey with longitudinal KPI comparison. | Location: Ireland Details: The 2021 Conti ransomware attack led to shutdown of all connected systems, with loss of electronic health records, radiology and laboratory information systems, imaging distribution, email, scheduling platforms, telemedicine, and inter-site communication. Community diagnostic pathways and primary care messaging were also disabled. | Inability to access historical imaging and laboratory results prevented assessment of treatment response and eligibility, delaying or excluding patients from trials and risking deviation from time-sensitive treatment schedules. Radiotherapy trials were particularly affected because interruption of fractionated treatment risks loss of tumour control, while surgical and diagnostic delays compounded existing waiting times. |
| Russell et al [97] | National mixed-methods orthopaedic-registry impact study. | Location: Ireland Details: The 2021 Conti ransomware attack that caused disconnection of approximately 70,000 devices and shutdown of national health IT infrastructure hosting the central registry database. Hospitals lost access to the online registry for a median of 134 days, with only intermittent download capability after approximately eight weeks. | Patients experienced indirect harm through cancelled or postponed operations and disrupted clinic flow, while operating room efficiency fell because perioperative documentation reverted to paper. The prolonged downtime generated 2850 additional staff hours for manual data reconciliation at an estimated cost of USD 181,000 - 216,000, diverting clinical personnel from routine care and extending recovery of normal services despite preservation of registry data integrity. |
| Kelly et al [54] | Single-centre retrospective trauma-outcomes study with expert commentary. | Location: USA  Details: The 2017 SamSam ransomware infiltrated the hospital network through brute-force access to a web server, encrypted clinical data, and rendered core systems inoperable for approximately six to eight weeks. Unlike other attacks such as WannaCry or with phishing, SamSam generated complex password combinations to access the server and infected other computers until the program saturated the network. | Operative volume decreased and both ICU and total hospital length of stay were slightly longer during the downtime. Care delivery became entirely paper-based, requiring substantial workflow adaptation and increasing operational burden, and the prolonged loss of digital systems created latent safety risks and inefficiencies that were not fully captured by the available outcome metrics. |
| Feeley et al [61] | Multicentre qualitative orthopaedic service evaluation. | Location: Ireland  Details: The 2021 cyberattack resulted in a national shutdown of electronic health records, the national imaging platform, laboratory systems, email, online referral pathways, and dictation services. To prevent viral spread across interconnected regional servers, shared systems were taken offline, eliminating remote access to imaging and laboratory data. | Loss of the EHR, national imaging and laboratory systems limited access to prior imaging, implant details, operative history, and baseline bloods, constraining safe clinical decision-making. For trauma patients, inability to view external radiology eliminated virtual triage, requiring in-person reassessment and repeat imaging, which delayed definitive management. |
| Neprash et al [56] | National event-study using Medicare claims and hospital data. | Location: USA Details: Ransomware attacks caused sharp but temporary activity reductions in the first week: inpatient admissions fell ~15–17%, outpatient visits ~22–35%, and ED visits ~10–19%, with parallel Medicare revenue losses (~21–28%), recovering within 2–3 weeks. | The ransomware attacks produced reductions in inpatient, outpatient, and emergency activity, indicating cancelled or deferred care that could delay diagnosis and treatment, allowing progression of acute and chronic conditions. Emergency department disruption required patients to travel substantially farther to the nearest functioning hospital in rural areas, potentially creating time-critical delays for acute conditions. |
| Abbou et al [13] | Single-centre controlled time-series analysis of hospital activity. | Location: Israel  Details: The 2021 DeepBlueMagic ransomware attack encrypted the hospital’s network and forced immediate disconnection from the internet, producing total loss of access to clinical and administrative systems. Staff could not view ward patient lists, prior medical histories, scheduled appointments, laboratory results, imaging, or internal email and intranet communication. | Clinical activity fell immediately, with sharp reductions in emergency attendances, hospital admissions, bed occupancy, surgical procedures, and ambulatory imaging, while elective operations and non-urgent admissions were cancelled and some patients were diverted or referred elsewhere. Disruptions created risks of delayed diagnosis and treatment, particularly for patients whose care depended on imaging, laboratory monitoring, multidisciplinary coordination, or blood products, as transfusions were minimised because the loss of historical blood-group data increased misidentification risk. |
| Mashinchi et al [98] | Descriptive case study using post-incident evidence. | Location: Ireland  Details: The 2021 Conti ransomware obtained initial access through a phishing email months before the event, followed by covert lateral movement using privileged accounts across a flat, poorly segmented national network. This resulted in loss of national and local EMRs, diagnostic and administrative applications, email and digital communication, and many clinical support systems. | Immediate loss of digital infrastructure caused widespread cancellation and postponement of outpatient activity, diagnostics, and elective care, diversion and rescheduling of services, and major constraints on clinical coordination across hospital and community settings. At system level, the scale and duration of application outages meant that access to care was reduced for large patient populations. |
| Gabbay‐Benziv et al [76] | Single-centre obstetric before-and-after activity study. | Location: Israel  Details: A 2021 ransomware attack disabled all computerized hospital functions, including the obstetric EHR, centralised fetal heart rate monitoring and managerial dashboard, laboratory and blood-bank systems, imaging access, appointment lists, telehealth for high risk pregnancies. | Departmental activity fell by ~5–10%, with a prolonged reduction in admissions and occupancy and a ~30% short-term drop in high-risk ambulatory visits, indicating disrupted surveillance for complex pregnancies. Loss of longitudinal records and prenatal data meant decisions relied on patient recall, increasing risk of delayed diagnosis, suboptimal risk stratification, and laboratory/transfusion error, which in turn drove conservative management and inter-hospital transfers. |
| Looi et al [52] | Documentary case analysis of a psychotherapy data breach. | Location: Finland Details: The hacker targeted the electronic health record database of the Vastaamo private psychotherapy provider, exploiting weak cybersecurity governance and security controls (lack of encryption, poor password practices, no multifactor authentication). The incident involved staged leaks of psychotherapy notes, followed by full database release and direct patient blackmail. | The primary patient harms were psychological and social rather than service-delivery disruption. The breach undermined trust in mental health services, threatened care engagement and disclosure in therapy, and created long-term confidentiality risks because leaked data are permanent and individually identifiable. |
| Chen et al [99] | Propensity-matched difference-in-differences study of hospital breaches. | Location: USA Details: Large protected-health-information data breaches that triggered federal investigation, multi-year regulatory monitoring, staff training, policy revision, and substantial health information-technology upgrades. | There was no reported significant short-term deterioration in care quality was detected around the breach event, but over time patients experienced faster care processes, with ED-to-admission time reduced by ~0.56 hours (~10%) and time to principal procedure reduced by ~0.18 days (~8%), alongside a small reduction in length of stay. AMI patients had a clinically meaningful 1-percentage-point absolute reduction in in-hospital mortality, while mortality for other groups was unchanged. |
| Ades et al [51] | Mixed-methods oncology service case study. | Location: USA  Details: The 2020 Ransomware attack caused total loss of all intranet servers, email communications and clinical systems (EMR, pharmacy information systems, laboratory, pathology and radiology). Restoration of EMR took 25 days, secure email took 28 days and radiology viewing systems took 40 days. | Outpatient oncology volume fell by 41% overall, with infusion centre visits dropping by 63% in the first week before partial recovery. Loss of EMR-embedded chemotherapy plan templates and electronic safety checks (allergy verification, dose calculation) removed automated safeguards from systemic therapy delivery, raising the risk of wrong-drug, wrong-dose and missed dose events. |
| Lippi et al [58] | Laboratory operational case report and editorial commentary. | Location: Italy  Details: A 2023 ransomware attack encrypted files across hospital servers. The hospital information system (HIS), laboratory information system (LIS), order entry, result reporting, intranet and all laboratory instrument connectivity were immediately lost. | The loss of electronic ordering and result display in wards removed automated reference ranges, alerts and clinical decision support safeguards - raising risk of errors. Manual transcription of results introduced the risk of identification and reporting errors. |
| Choudhuri et al [66] | Viewpoint article informed by published evidence. | Location: India Details: The 2022 ransomware attack on the All India Institute of Medical Sciences (AIIMS) Delhi compromised approximately 50 servers, rendered the hospital information system inaccessible for over 2 weeks, and forced reversion to paper-based records across inpatient, outpatient. | The paper synthesises evidence on how cyberattacks increase medication errors, causes delays in laboratory results, impairs communications, disrupts critical care workflows and clinical decision support - all raising the risk of patient safety incidents and adverse events. |
| Neprash et al [62] | National quasi-experimental study using Medicare claims. | Location: USA  Details: Cyberattacks (predominantly ransomware) disrupted hospital electronic health records, imaging services, medication dispensing systems and other digital infrastructure. | Hospital activity fell significantly during the first week of a cyberattack, with emergency, inpatient and outpatient volumes decreasing by 17-24%. Patients already hospitalized when attacks began, experienced a 34-38% increase in in-hospital mortality. |
| Davids et al [79] | Single-centre cross-sectional clinician survey. | Location: South Africa Details: In 2024 the NHLS suffered a ransomware attack, which resulted in the inability to utilise IT systems, including the laboratory information system (LIS) (InterSystems TrakCare® Lab Enterprise, Cambridge, Massachusetts, United States). Consequently, many automated processes reverted to manual operations, while new and historic patient laboratory results were inaccessible. | Clinicians reported disruptions to their daily workload and patient care, with 84% experiencing increased stress, 78% describing delayed diagnoses, 73% reporting delays in treatment decisions, 65% describing delayed patient referrals and 62% identifying emergency surgery delays. |
| McCarthy et al [78] | National multicentre hip-fracture registry study. | Location: Ireland Details: In 2021 the HSE of Ireland was subject to a ransomware attack resulting in a national disruption to the IT infrastructure utilised by the health service. | The cyberattack significantly reduced compliance with several Irish Hip Fracture Standards. Surgery within 48 hours decreased from 75.3% to 69.5%, timely orthopaedic ward admission declined (27.7% to 24.4%) and hospital acquired pressure ulcers increased (2.1% to 3.3%). |
| Faul et al [67] | Single-centre radiotherapy network response case study. | Location: Ireland Details: In 2021, the Health Service Executive (HSE) of Ireland experienced a major ransomware cyberattack. The HSE initially took down all of its IT systems to protect its core systems. | Radiotherapy services were temporarily suspended, resulting in treatment interruptions and delays while services were progressively restored over a two-week period. Patients receiving urgent, paediatric and palliative radiotherapy were prioritised, treatment schedules were modified, certain patients were transferred to private centres and staff work extended hours. |
| Frisch et al [65] | Single-centre pathology quality-assurance and recovery case study. | Location: USA Details: A systemwide cyberattack in 2020 affecting the University of Vermont Medical Center’s (UVMMC’s) electronic health record (EHR) system, Epic (Epic Systems), causing it to be completely locked throughout the downtime. | Intraoperative pathology assessments fell due to reduced surgical volume. Extensive staff time was needed to retrospectively reconcile lab records and billing. Manual reporting increased pathology report amendment rates from 1.6% to 4.2% during the downtime period. |
| Al Haddad et al [63] | Single-centre clinical-laboratory resilience case study. | Location: Lebanon  Details: In 2023, a ransomware attack affected the clinical laboratory at NDS-US, a university hospital in Lebanon, shutting down all IT systems. The laboratory experienced an immediate halt to electronic orders and reporting. | Laboratory testing capacity fell to 40-50% of normal activity during the initial downtime, with elective testing reduced by around 80% while emergency and ICU investigations were prioritised. Manual workflows increased workload and turnaround times. |
| O'Shea et al [69] | Technical development and evaluation study of a radiotherapy support tool during a cyberattack | Location: Ireland  Details: The May 2021 ransomware attack on the Irish Health Service Executive resulted in loss of electronic patient data and record-and-verify systems, preventing delivery of radiotherapy for up to 12 days. | Unscheduled interruptions to radiotherapy treatments lead to decreased tumour control, such that rapid cell repopulation in the tumour may occur due to the absent radiation dose. For oncology patients, this can lead to the recurrence and deterioration of their disease. |
| Rajput et al [100] | Primary-care policy commentary based on published and administrative evidence. | Location: UK  Details: In 2024, Synnovis, a partnership between SYNLAB and King’s College Hospital was hit by a ransomware cyberattack demanding US$50 million. The disruption also propagated to the 194 general practice surgeries in southeast London using the Synnovis platform. | The cyberattack limited access to pathology services and severely disrupted patient diagnostics, blood transfusion processing, and result reporting. Final data from NHS England revealed that the disruption led to the cancellation of 10 152 outpatient appointments and 1710 elective procedures being delayed in secondary care. |
| Fabrice et al [70] | Single-centre qualitative case study using interviews and internal records. | Location: France  Details: In 2021 Dax General Hospital was the target of a major cyberattack that led to the complete shutdown of its Information Technology systems. The Ryuk ransomware compromised approximately 85% of the hospital's 150 servers and rendered all backups inaccessible. | Medication databases, supplier records, ward stock lists, prescriptions, treatment protocols and historical data had to be reconstructed manually. Most chemotherapy sessions were initially postponed, cancelled or transferred, with selected treatments maintained through manual double checks. In the hospital sterilisation system, the loss of electronic traceability prevented staff from linking instrument sets to validated cycles; sterilisation activity was suspended and surgical procedures were cancelled. |

Reference list:

11. van Boven LS, Kusters RWJ, Tin D, et al. Hacking acute care: a qualitative study on the health care impacts of ransomware attacks against hospitals. Ann Emerg Med. Jan 2024;83(1):46-56. [doi: ] [Medline: 37318433]

13. Abbou B, Kessel B, Ben Natan M, et al. When all computers shut down: the clinical impact of a major cyber-attack on a general hospital. Front Digit Health. 2024;6:1321485. [doi: ] [Medline: 38433989]

45. Oliver M, Pearce A, Stillwaugh L, Leszczynski K. The impact of a cyberattack at a radiation oncology department: immediate response and future preparedness. Adv Radiat Oncol. 2022;7(5):100896. [doi: ] [Medline: 36148381]

46. Filipec O, Plasil D. The case of the benešov hospital hit by ryuk ransomware, and lessons learned. Obrana a strategie, University of Defence; 2021. [doi: ]

47. Pontier N, Orre M, Martin M. Cyberattack at Dax hospital: presentation of the facts, consequences and feedback. Cancer Radiother. Oct 2022;26(6-7):938-940. [doi: ] [Medline: 36028420]

48. Keogh RJ, Harvey H, Brady C, et al. Dealing with digital paralysis: surviving a cyberattack in a National Cancer center. J Cancer Policy. Mar 2024;39:100466. [doi: ] [Medline: 38176467]

49. Hoffman TW, Baker JF. Navigating our way through a hospital ransomware attack: ethical considerations in delivering acute orthopaedic care. J Med Ethics. Feb 2023;49(2):121-124. [doi: ] [Medline: 35197299]

50. Cassim S, Chapanduka ZC. Cyberattack on the National Health Laboratory Service of South Africa - implications, response and recommendations. S Afr Med J. Nov 29, 2024;114(12):e2549. [doi: ] [Medline: 41378543]

51. Ades S, Herrera DA, Lahey T, et al. Cancer care in the wake of a cyberattack: how to prepare and what to expect. JCO Oncol Pract. Jan 2022;18(1):23-34. [doi: ] [Medline: 34339260]

52. Looi JC, Allison S, Bastiampillai T, et al. Cybersecurity lessons from the Vastaamo psychotherapy data breach for psychiatrists and other mental healthcare providers. Australas Psychiatry. Feb 2025;33(1):106-110. [doi: ]

53. Ghafur S, Kristensen S, Honeyford K, Martin G, Darzi A, Aylin P. A retrospective impact analysis of the WannaCry cyberattack on the NHS. NPJ Digit Med. 2019;2(1):98. [doi: ] [Medline: 31602404]

54. Kelly WH, Narvaez JRF, Hu J, et al. Triumph over adversity: unlocking optimal trauma outcomes during healthcare ransomware attacks. Injury. Dec 2023;54(12):111046. [doi: ] [Medline: 37826882]

55. Pham TT, Loo TM, Malhotra A, et al. Ransomware cyberattack associated with cardiac arrest incidence and outcomes at Untargeted, Adjacent Hospitals. Crit Care Explor. Apr 2024;6(4):e1079. [doi: ] [Medline: 38605720]

56. Neprash HT, McGlave CC, Rydberg K, Henning-Smith C. What happens to rural hospitals during a ransomware attack? Evidence from Medicare data. J Rural Health. Sep 2024;40(4):728-737. [doi: ] [Medline: 38494590]

57. Nelson CJ, Soisson ET, Li PC, et al. Impact of and response to cyberattacks in radiation oncology. Adv Radiat Oncol. 2022;7(5):100897. [doi: ] [Medline: 36148379]

58. Lippi G, Ferrari A. Lessons learnt in medical laboratories during a disruptive cyber-attack. J Lab Precis Med. 2024;9:18-18. [doi: ]

59. Dameff C, Tully J, Chan TC, et al. Ransomware attack associated with disruptions at adjacent emergency departments in the US. JAMA Netw Open. May 1, 2023;6(5):e2312270. [doi: ] [Medline: 37155166]

60. Abouk R, Powell D. Ransomware attacks, ED visits and inpatient admissions in targeted and nearby hospitals. JAMA. Jun 25, 2024;331(24):2129-2131. [doi: ] [Medline: 38809568]

61. Feeley A, Lee M, Crowley M, et al. Under viral attack: an orthopaedic response to challenges faced by regional referral centres during a national cyber-attack. Surgeon. Oct 2022;20(5):334-338. [doi: ] [Medline: 34782238]

62. Neprash H, McGlave C, Nikpay S. Hacked to pieces? The effects of ransomware attacks on hospitals and patients. Am Econ J Econ Policy. Feb 1, 2026;18(1):256-281. [doi: ]

63. Al Haddad C, Boutros T, Finianos P, Germanos M. When code crashes the lab: operational resilience in clinical laboratories amid a cyberattack - a case study from a university hospital. Ann Biol Clin (Paris). Aug 26, 2025;83(4):414-424. [doi: ] [Medline: 40770901]

64. Iacobucci G. NHS makes urgent appeal for blood donations after cyberattack on London hospitals. BMJ. 2024:q1277. [doi: ]

65. Frisch NK, Gibson PC, Stowman AM, et al. Anatomy of a cyberattack: part 4: quality assurance and error reduction, billing and compliance, transition to uptime. Am J Clin Pathol. Jul 1, 2022;158(1):18-26. [doi: ] [Medline: 35188946]

66. Choudhuri B, Prakash J, Pal B, Veenith T. Cyber-resilient intensive care unit: ransomware is a patient-safety crisis-a resource-stratified approach for India. Indian J Crit Care Med. 2026;30(5):363-367. [doi: ] [Medline: 42415897]

67. Faul C, Robinson J, Carey J, et al. Effect of the cyberattack targeting the Irish health system in May 2021 on radiation treatment at St. Luke’s Radiation Oncology Network. Adv Radiat Oncol. 2022;7(5):100993. [doi: ] [Medline: 36148375]

68. Moore G, Khurshid Z, McDonnell T, Rogers L, Healy O. A resilient workforce: patient safety and the workforce response to a cyber-attack on the ICT systems of the national health service in Ireland. BMC Health Serv Res. Oct 17, 2023;23(1):1112. [doi: ] [Medline: 37848947]

69. O’Shea K, Coleman L, Fahy L, Kleefeld C, Foley MJ, Moore M. Compensation for radiotherapy treatment interruptions due to a cyberattack: an isoeffective DVH‐based dose compensation decision tool. J Applied Clin Med Phys. Sep 2022;23(9):e13716. URL: <https://aapm.onlinelibrary.wiley.com/toc/15269914/23/9> [Accessed 2026-08-24] [doi: ]

70. VidalF, Merlet M, Rucheton A, et al. Retour d’expérience sur une cyberattaque au sein d’un hôpital général: impacts et résilience d’une Pharmacie à Usage Intérieur PUI et de son unité de stérilisation. Le Pharmacien Clin. 2026;61(3):258-274. [doi: ]

71. Harrison AS, Sullivan P, Kubli A, et al. How to respond to a ransomware attack? One radiation oncology department’s response to a cyber-attack on their record and verify system. Pract Radiat Oncol. 2022;12(2):170-174. [doi: ] [Medline: 34644601]

72. Perry H, Tsai EM, Perusse K, Herschorn SD, Watson EJ. Breast imaging during a cyberattack and global pandemic: what we did to pick up the pieces. Semin Ultrasound CT MR. Feb 2023;44(1):18-22. [doi: ] [Medline: 36792269]

73. Stowman AM, Cacciatore LS, Cortright V, et al. Anatomy of a cyberattack: part 3: coordination in crisis, development of an incident command team, and resident education during downtime. Am J Clin Pathol. Jun 7, 2022;157(6):814-822. [doi: ] [Medline: 35188562]

74. Harvey H, Carroll H, Murphy V, et al. The impact of a national cyberattack affecting clinical trials: the cancer trials Ireland experience. JCO Clin Cancer Inform. Apr 2023;7:e2200149. [doi: ] [Medline: 37053539]

75. Devi S. Cyber-attacks on health-care systems. Lancet Oncol. Apr 2023;24(4):e148. [doi: ] [Medline: 36934730]

76. Gabbay-Benziv R, Ben-Natan M, Roguin A, et al. When the lights go down in the delivery room: lessons from a ransomware attack. Int J Gynaecol Obstet. Aug 2023;162(2):562-568. [doi: ] [Medline: 36740900]

77. Zhao JY, Kessler EG, Yu J, et al. Impact of trauma hospital ransomware attack on surgical residency training. J Surg Res. Dec 2018;232:389-397. [doi: ] [Medline: 30463746]

78. McCarthy CJ, Hickey P, O’Daly BJ, McGoldrick NP, Quinlan JF. The effect of a national healthcare cyberattack on the Irish hip fracture standards. Surgeon. Aug 2026;24(4):241-245. [doi: ] [Medline: 42091386]

79. Davids A, Fataar A, Zama A, et al. [Clinicians’ perspectives on the impact of a ransomware attack on a chemical pathology laboratory at a tertiary hospital in South Africa. EJIFCC. Mar 2026;37(2):268-280. [Medline: 42006506]](Clinicians%E2%80%99%20perspectives%20on%20the%20impact%20of%20a%20ransomware%20attack%20on%20a%20chemical%20pathology%20laboratory%20at%20a%20tertiary%20hospital%20in%20South%20Africa.%20EJIFCC.%20Mar%202026;37(2):268-280.%20%5BMedline:%2042006506%5D)

80. Goodwin A, Wilburn C, Wojewoda C, et al. Anatomy of a cyberattack: part 2: managing a clinical pathology laboratory during 25 days of downtime. Am J Clin Pathol. May 4, 2022;157(5):653-663. [doi: ] [Medline: 35188951]

81. Stowman AM, Frisch N, Gibson PC, et al. Anatomy of a cyberattack: part 1: managing an anatomic pathology laboratory during 25 days of downtime. Am J Clin Pathol. Apr 1, 2022;157(4):510-517. [doi: ] [Medline: 35188549]

82. Klatt MK. Case study analysis: cybersecurity breach at metropolitan health systems. In: In Multisector Insights in Healthcare, Social Sciences, Society, and Technology. IGI Global Scientific Publishing; 2024:115-135. [doi: ]

83. Haase KK, Whitworth MM, Yalamanchili K. Clinicians’ experiences and reflections from a health system cyberattack. J Am Coll Clin Pharm. Jun 2021;4(6):738-742. URL: <https://accpjournals.onlinelibrary.wiley.com/toc/25749870/4/6> [doi: ]

84. Fink J, Gresko J. Consequences of the change healthcare cyberattack continue. Pharm Times. 2024. URL: <https://www.pharmacytimes.com/view/consequences-of-the-change-healthcare-cyberattack-continue> [Accessed 2026-08-25]

85. Powell J, Monahan R, Slevin B, O’Hara M, O’Connell NH, Dunne CP. Coping with contagions: maintaining infection prevention and control in an Irish tertiary hospital during COVID and a debilitating nationwide ransomware virus in May 2021. J Hosp Infect. Aug 2021;114:180-181. [doi: ] [Medline: 34052282]

86. Santalo O, Perez G, Lorich C, et al. Defining key pharmacist and technician roles in response to a hospital downtime or cyberattack. J Am Pharm Assoc (2003). 2022;62(5):1518-1523. [doi: ] [Medline: 35466072]

87. Gourd E. Increase in health-care cyberattacks affecting patients with cancer. Lancet Oncol. Sep 2021;22(9):1215. [doi: ] [Medline: 34332657]

88. Zhao JY, Kessler EG, Guo WA. Interprofessional communication goes up when the electronic health record goes down. J Surg Educ. 2019;76(2):512-518. [doi: ] [Medline: 30253982]

89. Leonard F, O’Reilly H, Blackburn C, et al. Learnings from a national cyberattack digital disaster during the SARS-CoV-2 pandemic in a pediatric emergency medicine department. Disaster Med Public Health Prep. Jun 26, 2023;17:e419. [doi: ] [Medline: 37357951]

90. Strong H, Pickles A, Hartery A. Lessons learned from a cyberattack on the healthcare system of newfoundland and labrador: a radiology perspective. Can Assoc Radiol J. Aug 2022;73(3):601-602. [doi: ] [Medline: 35199607]

91. Aljaidi M, Alsarhan A, Samara G, et al. NHS wannacry ransomware attack: technical explanation of the vulnerability, exploitation, and countermeasures. Presented at: 2022 International Engineering Conference on Electrical, Energy, and Artificial Intelligence (EICEEAI); Nov 29 to Dec 1, 2022:1-6; Zarqa, Jordan. [doi: ]

92. Chen PH, Bodak R, Gandhi NS. Ransomware recovery and imaging operations: lessons learned and planning considerations. J Digit Imaging. Jun 2021;34(3):731-740. [doi: ] [Medline: 34159418]

93. Duffy C, Murray C, Boran G, Srinivasan R, Kane A, Leonard A. Survey of laboratory medicine’s national response to the HSE cyberattack in the Republic of Ireland. Ir J Med Sci. Apr 2024;193(2):889-896. [doi: ] [Medline: 37737914]

94. Stowman AM, Kalof AN. Surviving a cyberattack in anatomic pathology: disaster response and creation of an incident command system. AJSP Rev Rep. 2022;27(4):171-176. [doi: ]

95. Tarka M, Blankstein M, Schottel P. The crippling effects of a cyberattack at an academic level 1 trauma center: an orthopedic perspective. Injury. Apr 2023;54(4):1095-1101. [doi: ] [Medline: 36801172]

96. Slattery FJ, Farrell E, Lacy PD. The effect of the 2021 Irish cyber-attack on otolaryngology outpatient non-attendance at a model 4 hospital in the post-COVID era. Cureus. Jul 2022;14(7):e26944. [doi: ] [Medline: 35989756]

97. Russell SP, Fahey E, Curtin M, Rowley S, Kenny P, Cashman J. The Irish national orthopaedic register under cyberattack: what happened, and what were the consequences? Clin Orthop Relat Res. Sep 1, 2023;481(9):1763-1768. [doi: ] [Medline: 37036406]

98. Mashinchi MI, Acton T, Datta PM. When healthcare becomes sick: recovering from ransomware. J Inf Technol Teach Cases. May 2026;16(1):92-101. [doi: ]

99. Chen D, Chou SY, Peng XD. Impacts of hospital data breach on healthcare quality. Health Serv Res. Jun 2025;60(3):e14439. [doi: ] [Medline: 39789912]

100. Rajput K, Darzi A, Ghafur S. Overlooked and under-reported: the impact of cyberattacks on primary care in the UK National Health Service. Lancet Digit Health. Jul 2025;7(7):100879. [doi: ] [Medline: 40414783]

101. Zhao JY, Kessler EG, Guo WA. Surgical interprofessional communication during a hospital ransomware attack. J Am Coll Surg. 2018;227(4):S165. [doi: 10.1016/j.jamcollsurg.2018.07.351]
