# Supplementary material for: Unveiling Patient-Level Harms and At-Risk Clinical Groups During Hospital Cyberattacks: Systematic Review of Global Case Studies and Social Media Data
Source: J Med Internet Res. 2026 Sep 28;28:e88257. doi: 10.2196/88257 (PMC13622069; doi:10.2196/88257)
Supplement: Supplementary file 4 [file jmir-v28-e88257-s003.docx]

| **Article Number** | **References** | **Publication Date** | **Cyber Attack Date** | **Country** | **Study Design and Setting** | **Organisation / Population Affected** | **Attack Characteristics and Systems Disrupted** | **Impact on patients and clinical operations** | **URL** |
| --- | --- | --- | --- | --- | --- | --- | --- | --- | --- |
| 1  2 | Moore et al [68]  Ghafur et al [53] | 2023  2019 | May 2021  May 2017 | Ireland  UK | Qualitative study using focus groups (n=36), followed by a thematic analysis, to evaluate staff experiences of the cyberattack across departments of radiology, pathology, radiotherapy, maternity, dentistry, elderly care and disability services.  Retrospective quantitative observational study using national administrative data to quantify service-level impact of the Wannacry attack. The analysis compared activity in affected vs unaffected NHS organisations in England across the attack period. | The Irish 'Health Service Executive' (HSE) - a national public health system. The HSE is responsible for the national public health service in Ireland, serving a population of 5.1 million people. The interviewed study participants were from a large university hospital, teaching hospital and community organisation, ensuring multidisciplinary involvement of frontline and managerial staff.  Secondary care NHS trusts in England (acute, community services and mental health), analysed at the organisational level. Selected outcomes included number of outpatient appointments cancelled, elective and emergency admissions to hospital, A&E attendances and deaths in A&E. | A criminal infiltration of the HSE’s IT systems using Conti ransomware. In response a Critical incident process was initiated which resulted in healthcare staff losing access to all HSE provided IT systems, including patient information, clinical care and laboratory systems.  The global ransomware attack known as 'WannaCry' affected multiple continents and organisations in May 2017. Although not the direct target, the UK NHS was one of the biggest casualties, with over 600 organisations affected, including 34 infected hospital trusts. Infected hospitals were blocked from their digital systems and medical devices, such as MRI scanners and healthcare records. | No direct measurement of clinical outcomes, however staff described major operational disruption and patient safety risk. Radiotherapy delays for patients due to cancellation of diagnostic and treatment procedures; Participants in acute and maternity services were most impacted due to complete reliance on electronic methods; where patient information systems were inaccessible clincial staff treated patients without knowing their full history, raising risk of medical errors.  Infected trusts had ~6% fewer total admissions, 4% fewer emergency admissions, 9% fewer elective admissions, 6% fewer ED attendances and ~50% more outpatient cancellations. Emergency care was redirected to neighbouring hospitals due to ED closures. | [https://pubmed.ncbi.](https://pubmed.ncbi.nlm.nih.gov/37848947/) [nlm.nih.gov/37848947/](https://pubmed.ncbi.nlm.nih.gov/37848947/)  [https://www.nature.](https://www.nature.com/articles/s41746-019-0161-6) [com/articles/s41746-019-0161-6](https://www.nature.com/articles/s41746-019-0161-6) |
| 3 | Stowman et al [73] | 2022 | October 2020 | USA | Descriptive single-centre case study of an academic anatomic pathology lab, within a multi-hospital health network during a prolonged cyberattack. The report provides a narrative operational analysis of lab management, communication and staff redeployment over the attack period. | University of Vermont (UVM) Health Network, with five affiliated facilities across two USA states. Primary focus on the pathology laboratory and the teams it interfaced with (surgical teams, oncology, radiology). | A malicious email that injected a piece of malware into a device that - once reconnected to the organisation network - facilitated the infected device to launch a variety of attacks on the network. The ransomware took out the primary networking and computing infrastructure of the hospital system. | Clinical outcomes were not directly quantified, instead the paper reported service disruptions that had downstream effects on patient care. Radiology was unable to provide normal imaging support, oncology patients had to be referred elsewhere and surgical activity required strict triage. Due to manual reporting there was delayed result transmission and created risks of delayed diagnosis and treatment. | [https://pubmed.ncbi.](https://pubmed.ncbi.nlm.nih.gov/35188562/) [nlm.nih.gov/35188562/](https://pubmed.ncbi.nlm.nih.gov/35188562/) |
| 4  5  6 | Stowman et al [81]  Goodwin et al [80]  Perry et al [72] | 2022  2022  2023 | October 2020  October 2020  Oct 2020 | USA  USA  USA | Descriptive single-centre case study of the anatomic pathology service at University of Vermont Medical Center, within a multi-hospital regional network.  Single-centre, detailed operational case study of the clinical pathology service at an academic level-1 trauma centre and regional laboratory (University of Vermont Medical Centre), during a cyberattack and 25-day network shutdown.  Single-centre, descriptive operational case study of a breast imaging division at the University of Vermont Medical Center (UVMMC) - a 620 bed academic, tertiary care institution and level 1 trauma center located in northwestern Vermont which serves as a referral center for much of Vermont and northern New York. | The University of Vermont Medical Center (UVMMC) - a regional tertiary referral pathology service providing histopathology and cytology for patients. The affected clinical population included surgical, oncological and general medicine patients, whose diagnosis and treatment depended on laboratory turnaround times.  The University of Vermont Medical Centre averages 1.2 million annual patient care encounters and is a referral center for more than 1 million people in the region. The CP laboratory reports approximately 3.2 million tests annually for hospital inpatients, emergency patients, outpatients, and external referral clients.  Specialist breast imaging service delivering screening, diagnostic mammography, ultrasound, MRI, image-guided biopsy, and wire localisation for a regional referral population. Affected groups included symptommatic patients on urgent cancer pathways, patients awaiting image-guided procedures, screening populations and those requiring follow up. | System-wide ransomware that resulted in an immediate shutdown of all hospital IT, including the electronic health record, radiology, pharmarcy, scheduling, billing, email and networked equipment. There was loss of digital tracking, reporting, access to prior results and clinical information.  A ransomware-related shutdown of hospital systems, that resulted in loss of the electronic health record, middleware, electronic ordering, pneumatic tube transport, courier badge access, digital phones/faxes, paging, shared drives and instrument connectivity. There was an immediate shift to manual effort requiring increased staffing and safety checks. Pathology services were immediately reduced to urgent services and the team closed ~90% of outpatient phlebotomy sites to reduced the volume of collections.  Following a succesful phishing attack perpetrated via email, a Trojan malware infected a UVMMC employee laptop while it was off the network. When this laptop was reconnected to the network, the malware, and subsequently ransomware, was able to infiltrate the virtual computer server environment, encrypting all virtual hard disks. The picture archiving and communication system (PACS), radiology information system (RIS) and the dictation system froze, and shortly thereafter email, the hospital paging system, as well as the internet and intranet became inaccessible as well. The Electronic Health Record (EHR) was pro-actively brought off-line to protect sensitive patient data from the attack, rendering it impossible to access patient care information, including history, results, or even appointment schedules and patient contact information. | The researchers did not measure clinical outcome metrics, but reported diverse care-delivery impacts with patient safety implications: increased turnaround times, reduced in-house processing capacity, inability to access previous pathology or clinical data and high-risk manual identification.  No direct clinical outcome measures, but major and safety impacts with patient-care implications. This included substantial turnaround-time prolongation, reduced access to results for care teams, missed or unlocatable results and loss of cumulative data and past health record information.  Significant access on patient access to services, with most screening and routine diagnostics being deffered, many patients being turned away from appointments, and patients being advised to seek imaging at other institutions. Screening mammograms could not be reported within the mandated 30-day time frame, requiring regulatory notification and diagnostic interpretation was constrained by lack of prior imaging and clinical data. Large reporting backlogs delayed final results and billing for months, creating prolonged uncertainty for patients and potential delay in cancer detection. | [https://academic.oup.](https://academic.oup.com/ajcp/article/157/4/510/6533547) [com/ajcp/article/157/4/5](https://academic.oup.com/ajcp/article/157/4/510/6533547) [10/6533547](https://academic.oup.com/ajcp/article/157/4/510/6533547)  [https://academic.oup.](https://academic.oup.com/ajcp/article/157/5/653/6533594) [com/ajcp/article/157/5/6](https://academic.oup.com/ajcp/article/157/5/653/6533594) [53/6533594](https://academic.oup.com/ajcp/article/157/5/653/6533594)  [https://pubmed.ncbi.](https://pubmed.ncbi.nlm.nih.gov/36792269/) [nlm.nih.gov/36792269/](https://pubmed.ncbi.nlm.nih.gov/36792269/) |
| 7 | Klatt [82] | 2024 | 2024 | USA | Strategic organisational case study of a US hospital (Metropolitan systems, Ohio), using a risk-management framework. The authors analyse repeated breach events (2015-2023) to identify vulnerabilities, assess organisational risk and plan for future events. | Large multi-site hospital system with dependence on shared network infrastructure and third-party vendors for data storage and services. Recurrent breaches create organisation-wide operational risks and privacy risks for entire patient population. | Multiple cyberattack incidents involving malware and ransomware-related data breaches, with both internal and external threat vectors. The chapter maps key technical vulnerability domains, highlighting how compromise of the network enables enterprise-wide system control and attack susceptibility. | No direct clinical outcomes measures, instead patient impact is conceptualised as loss of confidentiality of health information, increased long-term exposure to fraud and systemic service risk due to organisational disruption which may impede patient care. | [https://www.igi-global.](https://www.igi-global.com/chapter/case-study-analysis/340570) [com/chapter/case-study-analysis/340570](https://www.igi-global.com/chapter/case-study-analysis/340570) |
| 8 | Haase et al [83] | 2021 | September 2020 | USA | Single-site, practice-based descriptive reflection from an interprofessional team, within a 495-bed tertiary hospital in the Unvieral Health Services Network after the September 2020 Ransomware attack.  Focused on changes to clinical workflow, medication processes, training gaps and team adaption during the hospital IT  downtime. | A 495-bed for-profit hospital in Texas, with three intensive care units (ICUs) acting as a level 3 trauma center and the major tertiary care center for a 26-country region in Texas and four neighbouring states. All ICU patients were affected due to impacts on prescribing, patient monitoring, documentation, laboratory/radiology access, medication administration and clincial communication. | Suspected ransomware leading to a proactive shutdown of all clinical computer systems, resulting in complete loss of the electronic health record, computerised order systems, medication administration record systems and clinical decision support systems. Care shifted to paper notes and handwritten orders, phone/fax transmission of medication orders, manual allergy and interaction checking, manual medication reconciliation, hand-written labels and retrospective reconciliation for billing. | Authors highlighted the increased risk of medication administration errors due to loss of the barcode medication system, overrides were required creating risks of wrong drug, wrong doses and adverse events. Support systems, such as electronic drug information sources, were also lost, raising the risk of unsafe prescribing. Staff highlighted particular risks with loss of the heparin protocol, where reliance on newer automated systems may lead to risks in anticoagulation management. Maintaining medication access records was challenging, raising the risk of omitted or duplicated doses (e. g. antibiotics). The researchers also detailed how they could not fully quantify harm and impacts on patient safety because such adverse events are typically documented through the same systems impacted by the event. | [https://accpjournals.](https://accpjournals.onlinelibrary.wiley.com/doi/full/10.1002/jac5.1423) [onlinelibrary.wiley.](https://accpjournals.onlinelibrary.wiley.com/doi/full/10.1002/jac5.1423) [com/doi/full/10.](https://accpjournals.onlinelibrary.wiley.com/doi/full/10.1002/jac5.1423)  [1002/jac5.1423](https://accpjournals.onlinelibrary.wiley.com/doi/full/10.1002/jac5.1423) |
| 9 | Fink and Gresko [84] | 2024 | February 2024 | USA | Descriptive report outlining the operational and clinical consequences of the Change Healthcare Ransomware attack across the USA healthcare system, focusing specifically on pharmacy services, medication access, reimbursement and claims processing. | Change Healthcare was the main organisation affected, which is a national medical billing and transaction clearing house. They process 15 billion medical claims annually (40% of all claims in the USA). Hospitals, outpatient providers and >90% of USA pharmacies were indirectly affected. | BlackCat/ALPHV, allegedly used compromised credentials to gain access to CHC’s system, that lacked multifactor authentication, and deploy the ransomware while stealing data. This lead to the immediate disconnection of systems to contain the spread, preventing hospitals and pharmacies from processing claims. | No formal clinical outcome metrics, but clear care-access and system-capacity effects. There was immediate loss of medication access pathways, patients were unable to obtain medicines unless paying out-of-pocket, and there was long-term potential harm through confidentiality breach and fraud exposure. | [https://www.](https://www.pharmacytimes.com/view/consequences-of-the-change-healthcare-cyberattack-continue) [pharmacytimes.](https://www.pharmacytimes.com/view/consequences-of-the-change-healthcare-cyberattack-continue) [com/view/consequence](https://www.pharmacytimes.com/view/consequences-of-the-change-healthcare-cyberattack-continue) [s-of-the-change-healthcare-cyberattack-continue](https://www.pharmacytimes.com/view/consequences-of-the-change-healthcare-cyberattack-continue) |
| 10 | Powell et al [85] | 2021 | May 2021 | Ireland | Single-centre descriptive service report detailing how infection prevention and control (IPC) and microbiology systems were maintained at University Hospital Limerick during the national HSE ransomware shutdown. The paper offers a real-time operational account of workflow change, surveillance loss and continuity measures. | University Hospital Limerick, a major tertiary hospital within the national HSE system. The attack affected all inpatients due to the loss of IPC surveillance, microbiology diagnostics, outbreak detection, bed allocation and patient-flow management systems. | National ransomware attack that encrypted files and disabled user access, leading to a complete shutdown of the HSE IT Infrastructure. There was a loss of telecommunications (including voice over internet protocol), access to data bases and access to clinical and support services. | Delayed notification of notifiable diseases increased risks relating to outbreaks, point-prevalence screening also had to cease reducing the hospitals ability to detect transmision. The loss of electronic alerts meant isolation and cohorting was curtailed, and the lack of COVID19 results meant a higher risk of inappropriate patient placement and exposure. | [https://www.](https://www.journalofhospitalinfection.com/article/S0195-6701(21)00212-7/fulltext) [journalofhospitalinfectio](https://www.journalofhospitalinfection.com/article/S0195-6701(21)00212-7/fulltext) [n.com/article/S0195-6701(21)00212-7/fulltext](https://www.journalofhospitalinfection.com/article/S0195-6701(21)00212-7/fulltext) |

| **Article Number** | **Reference** | **Publication Date** | **Cyber Attack Date** | **Country** | **Study Design and Setting** | **Organisation / Population Affected** | **Attack Characteristics and Systems Disrupted** | **Impact on patients and clinical operations** | **URL** |
| --- | --- | --- | --- | --- | --- | --- | --- | --- | --- |
| 11  12 | Devi [75]  Pontier et al [47] | 2023  2022 | February 2023  February 2021 | USA  France | Narrative analysis drawing on real-world cyberattacks, expert commentary and sector reports. Focuses on ransomware attacks affecting hospitals, national health services and health IT vendors, looking at the downstream effects on cancer care, emergency services and patient data security.  Single centre case report describing their experience over the cyberattack timeline, examining operational consequences and recovery actions. | Multiple healthcare delivery organisations and health iT suppliers (e.g. Lehigh Valley Health Network in the USA and the 'NHS 111' software provider Advanced). Affected populations studies included cancer patients, emergency patients requiring ambulance transfer, and hospital patients who dependended on compromised digital health records.  The french hospitals of Dax, Arles, Oloron-Sainte-Marie or Villefranche-sur-Saône, saw their computer system paralyzed following a computer attack. This report focused on the DAX hospital and its oncology-radiotherapy service that was significantly disrupted. Their service includes two accelerators, with which they treat about 80 patients per day (i.e. 900 per year) in external or stereotactic radiation therapy. | Predominantly ransomware attacks using malware to disrupt, damage or deny access to computer systems, before demanding payments from healthcare organisations.  A ransomware attack known as 'cryptolocker' that occured at 2am - the timing was thought to be tactical choice, given that less IT staff are available at night. Entry point was unclear and not thought to be via email, but instead via a vulnerability in the hospital firewall (Fortinet) | References the case of a German patient who died from an aortic aneurysm when her treatment was delayed due to a cyberattack that disrupted her care. Looks specifically at disruption to cancer care operations, reflecting on instances of patient documents and images being leaked into dark web.  Further, the author cites a report that revealed 23% of repsondents in healthcare IT described an increase in mortality following cyberattacks.  Clinical impacts on cancer patients were most apparent, with radiotherapy having to stop immediately, preventing treatment for an unclear duration. Patients were referred to nearby centres, with potential impacts of treatment delay and cancer progression. Once systems were restored, retrospective analysis identified deviations from treatment regimes including forgotten sessions for breast cancer patients. | [https://pubmed.ncbi.](https://pubmed.ncbi.nlm.nih.gov/36934730/) [nlm.nih.gov/36934730/](https://pubmed.ncbi.nlm.nih.gov/36934730/)  [https://pubmed.ncbi.](https://pubmed.ncbi.nlm.nih.gov/36028420/) [nlm.nih.gov/36028420/](https://pubmed.ncbi.nlm.nih.gov/36028420/) |
| 13  14 | Cassim and Chapanduka [50]  Keogh et al [48]. | 2024  2024 | June 2024  May 2021 | South Africa  Ireland | Single center experiential report from the haematopathology department at Tygerberg hospital, within a national laboratory network in South Africa. A narrative impact description with operational analysis and a proposed business continuity framework.  Mixed-methods single centre impact evaluation at Cork University Hospital (CUH) Cancer Centre, involving 120-day activity log review vs historical baselines, plus reflective essays and interviews wtih key stakeholders. | The National Health Laboratory Service (NHLS) is a nationwide public diagnostic laboratory system with over 8000 staff and is the sole provider of diagnostic pathology services to over 80% of the South African population.  CUH Cancer Centre within Ireland's public health service (HSE), covering a regional cancer population (~1.1 million) and multiple oncology services (surgery, radiotherapy, medical oncology/haematology, diagnostics, palliative care, clinical trials). | A ransomware attack named 'Blacksuit' that encrypted servers and applications, causing a complete shutdown of IT operations, including internet, intranet, the 'TrakCare' laboratroy information system and the central data warehouse containing decades of historical results. Core functions became inaccessible for over a month, including electronic test requesting, analyser result upload, the viewing and authorisation of results and access to prior results.  Conti ransomware that lead to all hospital IT systems being shutdown. Affected systems included email and patient administration systems, core cancer service platforms and diagnostics, radiology services (RIS/PACS), radiotherapy oncology information systems, lab middleware/LIMS connectivity and clinical management systems. | Authors detailed how clinician's ability to appropriately diagnose, manage and monitor their patients was directly affected by the immediate doubling of turnaround times for blood tests (e.g. Full blood count turnaround time increased from <2 hours to 4 hours during the attack). Testing capacity was reduced to urgent investigations only and previous laboratory history was unavailable, creating long-term implications for continuity of care and disease surveillance. The disruption also resulted in system-level effects that indirectly affected patient care, including increased staff workload and anxiety, suspension of teaching and research activities and erosion of public trust in the health system.  The cessation of cancer services had immediate impacts on patients, with the cancellation of radiotherapy treatments and tumour surgeries. Outpatient activity also fell by 50%, reducing care of patients in the community. Diagnostics were severely constrained (~90% drop in haematology and radiology capacity, ~75% drop for biochemistry), immediately impacting patient diagnosis and monitoring. The authors report median treatment gaps across different categories, with implications for patient disease progression and symptom worsening.  PET/CT and mammography stopped completely for 3 weeks,  leading to delays in diagnosis and screening. | [https://doi.org/10.](https://doi.org/10.7196/SAMJ.2024.v114i12.2549) [7196/SAMJ.2024.](https://doi.org/10.7196/SAMJ.2024.v114i12.2549) [v114i12.2549](https://doi.org/10.7196/SAMJ.2024.v114i12.2549)  [https://www.](https://www.sciencedirect.com/science/article/pii/S2213538323000838) [sciencedirect.](https://www.sciencedirect.com/science/article/pii/S2213538323000838) [com/science/article/pii/S](https://www.sciencedirect.com/science/article/pii/S2213538323000838) [2213538323000838](https://www.sciencedirect.com/science/article/pii/S2213538323000838) |
| 15 | Santalo et al [86] | 2022 | July 2021 | USA | Single site, practice-based operational report from a 350-bed acute care hospital in the USA. The authors describe how the pharmacy department responded to cyberattack-related downtime, reorganisation management workflows and identify operational issues. | All hospital patients requiring medicine were indirectly affected due to pharmacy services underpinning prescribing, verification, dispensing and administration across inpatient and outpatient care. | The cyberattack caused the hospital to simultaneously lose access to the electronic health record, admitting and registration systems, pharmacy information systems, barcode medication administration, automated dispensing cabinet servers, inventory management software, compliance software, diversion software, financial systems and clincial decision-support tools. | The researchers state that the disruptions to the pharmacy workflow and loss of automation can be manually intensive and unsafe for patients. Loss of barcode administration and clinical decision support can remove automated allergy checks, interaction screening and electronic verification. The increased turnaround times for processing can also lead to delays in treatment. The study shows that when medication systems fail, the probabilty of adverse drug events rises. | [https://pubmed.ncbi.](https://pubmed.ncbi.nlm.nih.gov/35466072/) [nlm.nih.gov/35466072/](https://pubmed.ncbi.nlm.nih.gov/35466072/) |
| 16  17  18  19 | Van boven et al [11]  Harrison et al [71]  Nelson et al [57]  Zhao et al [77] | 2024  2022  2022  2018 | 2022  April 2021  October 2020  April 2017 | USA  USA  USA  USA | A qualitative study using semistructured interviews with healthcare professionals and IT staff to explore their experiences and the challenges faced during and after a ransomware attack. Interviewees were both clinical staff and IT practitoners, experienced in hospital ransomware attacks in Europe and the USA (2017-2022), covering both acute phase and recovery.  Operational case report describing a ransomware incident affecting a radiation oncology department and six affiliated treatment departments. The paper examines emergency workflow, role allocation, communications during the event and phased recovery while the 'Record and Verify' (RVS) system was unavailable.  Single-network incident report detailing a cyberattack on the University of Vermont Health Network and the operational, technical and clinical response within the hospital radiation oncology service.  Mixed methods single centre study at an American College of Surgeons-verified level 1 trauma centre, following the SamSam ransomware attack that caused a 2 month shutdown of the health information system. Quantitative trauma-registry analysis compared admissions, injury severity, length of stay and mortality with the same period the previous year; a confidential likert scale survey of surgical residents (n=29) and semi-structured interviews with attending surgeons explored effects on clinical work, communication, documentation and training. | Four hospitals (secondary/tertiary, cover 200-400 beds) and their emergency care pathways. Bed counts ranged from 200 - 400 across the four sites examined, catchment overall was over >100,000 patients. All departments were affected in every incident.  A multi-site radiation oncology service treating patients on active fractionated radiotherapy, where accurate cumulative dose tracking is essential for safe delivery of treatment. The affected population therefore incldued patients who were mid-course and those due to start treatment, as the RVS was central to this process.  Patients recieving radiotherapy with curative or palliative intent, patients awaiting treatment initative and patients requiring imaging, chemotherapy or multidisciplinary oncological care across the hospital system. The most time-critical group were those on active fractionated curative courses were overall treatment time influences tumour control.  A level 1 academic trauma service in which all emergency surgical care continued using paper records and face-to-face communication. The affected clinical population comprised trauma patients requiring time-critical operative and peri-operative decision-making and the resident workforce on the frontline of patient care. | All ransomware incidents involved a loss of access to digital radiology and laboratory systems and a loss of EHR access. Further systems impacted including: Computers, internal internet access, internal telephone/email and digital medical systems.  Ransomware attack on the major RVS vendor, which forced an immediate shutdown of the system and removed the department's ability to verify dose delivery and treat patients. The RVS was unavailable for 2.5 weeks, with full functionaility not returning until after 4.5 weeks.  The cyberattack forced a precautionary shutdown of the hospital network. The University of Vermont Health Network (UVMHN) experienced multiple clinical system outages as a result of the cyberattack. Access to the internet, hospital servers, and remaining clinical systems was immediately halted by the information technology (IT) infrastructure team to minimize further propagation of malware.  The SamSam ransomware forced the immediate loss of the electronic medical record, imaging systme (PACS), e-prescribing, scheduling systems, communication tools and internet access. The transition occured without the structured safety process that historically supported paper systems. | Staff described conditions that could potentiate patient harms via delays, missing clinical histories and follow-up gaps.  Delays were described as massive and clinicians raised concerns about delays to patient care and lost follow up. On discussing patient safety, the researchers highlighted concerns around inaccessibility of the patient record and medication lists, lost diagnostic findings and the risks of overlooking patients with acute conditions in the waiting room because of crowding.  All treatments were initially stopped, with no patients being treated in the first 24 hours. Given that these are time-critical treatmetns, the associated treatment gaps for patient on therapy could affected cancer outcomes and progression.  Activity recovered in phases, meaning that delays and cancellations continued over the month of downtime.  All remaining treatments were cancelled ond ay 1 and clinicians had to triage patients, due to the risk of extended treatment breaks affecting oncological outcomes. The highest priority patients were transferred to affiliate centres or treated using simplified manual techniques, while others experienced planned delays.  Measured trauma outcomes (volume, severity, mortality) were not statistically different from the comparator year. However, qualitative and workflow data demonstrated multiple mechanisms of patient safey risk: the loss of EMR checks and balances, inability to review imaging or laboratory results, disorganised paper charge and reduced pre-operative case preperation created a lot of different places for mistakes to happen. One attending described a missed injury, in which absent electronic safety nets may have prevented detection. Clinical decision making became slower and dependant on verbal transmission of information, causing delays to care.  Remote supervision was no longer possible and residents  experienced significant stress with paper orders and medication prescribing. | [https://pubmed.ncbi.](https://pubmed.ncbi.nlm.nih.gov/37318433/) [nlm.nih.gov/37318433/](https://pubmed.ncbi.nlm.nih.gov/37318433/)  [https://pubmed.ncbi.](https://pubmed.ncbi.nlm.nih.gov/34644601/) [nlm.nih.gov/34644601/](https://pubmed.ncbi.nlm.nih.gov/34644601/)  [https://www.](https://www.sciencedirect.com/science/article/pii/S2452109422000045) [sciencedirect.](https://www.sciencedirect.com/science/article/pii/S2452109422000045) [com/science/article/pii/S](https://www.sciencedirect.com/science/article/pii/S2452109422000045) [2452109422000045](https://www.sciencedirect.com/science/article/pii/S2452109422000045)  [https://www.](https://www.journalofsurgicalresearch.com/article/S0022-4804(18)30477-3/fulltext) [journalofsurgicalresearc](https://www.journalofsurgicalresearch.com/article/S0022-4804(18)30477-3/fulltext) [h.com/article/S0022-4804(18)30477-3/fulltext](https://www.journalofsurgicalresearch.com/article/S0022-4804(18)30477-3/fulltext) |

| **Article Number** | **Reference** | **Publication Date** | **Cyber Attack Date** | **Country** | **Study Design and Setting** | **Organisation / Population Affected** | **Attack Characteristics and Systems Disrupted** | **Impact on patients and clinical operations** | **URL** |
| --- | --- | --- | --- | --- | --- | --- | --- | --- | --- |
| 20  21  22 | Gourd [87]  Zhao et al [88]  Leonard et al [89]. | 2021  2019  2023 | April 2021  Missing  May 2021 | USA  USA  Ireland | Analysis article drawing on institutional disclosures of cyberattack events, cybersecurity reports and expert commentary to describe ransomware incidents affecting oncology services and the growing vulnerability of radiotherapy infrastructure, particularly cloud-dependent systems.  Single centre qualitative study using semistructured interviews, analysed with a grounded theory approach. Conducted in a regional tertiary referral hospital and level 1 trauma centre that experienced a 2 month ransomware related EHR outage.  A single centre retrospective time series analysis conducted in a tertiary, university-affiliated paediatric emergency department in Ireland, with ~39,500 annual pre-pandemic attendances. The authors compared three equal 6-week periods - before the cyberattack, during the outage and after restoration, using operational data. | Multiple oncology providers using Elekta cloud-based radiotherapy software, including Northwestern memorial HealthCare, Cancer Centers of Southwest Oklhahoma, and Emory Saint Joseph's Hospital. Approximately 200,000 patients at one US system had sensitive data compromised. The affected clinical population comprised patients recieving time-critical cancer treatment.  Focused on surgical departments and surgical residents, with implications for hospitalised surgical and trauma patients whose care was delivered without access to the EHR, electronic prescribing or remotely viewable results.  The disruption affected a national public health system and, at the study site, all children attending a high volume tertiary paediatric emergency department. The department continued to function as a key access point for unscheduled acute care during the cyberattack and COVID19 pandemic. All clincal, adminstrative, and bed-management workflows in the ED were affected, due to patient registration, tracking, documentation, admissions, and discharge processes were dependant on national and hospital IT systems. | Ransomware attack on a 3rd party cloud platform hosting radiotherapy linear-accelerator software and associated oncology data. Disruption extended beyond data breach to loss of functionaility of treatment-delivery systems, causing cancellation or delay of radiotherapy sessions across 42 sites.  The cyberattack resulted in the complete loss of the EHR, requiring reversion to paper charts. Diverse results and order sets were impacted including laboratory and imaging results, medication lists, consultation requests, and prescribing workflows were no longer accessible.  The national ransomware attack resulted in the shutdown of core digital infrastructure, resulting in the loss of patient administration systems, emergency department information system (EDIS), laboratory and radiology access and all intranet/internet connectivity. | The cyberattack directly interrupted radiotherapy delivery, preventing treatment of patients. Furthermore, loss of access to referrals, imaging, and diagnostic workflows were linked to slower cancer screening and staging.  The loss of the EHR disrupted routine care and introduced new risks to patients, especially where regular processes took longer to complete and paper prescribing led to delayed medication access as community pharmacies refused paper scripts. Clinical information was harder to access and less reliable because staff had to rely on paper flows, staff reported that results were often misplaced or put in the wrong chart, raising the risk of medical error.  Median length of stay fell and critically ill patients moved more quickly from registration to triage, and form clinician review to disposition, largely because documentation requiremetns were reduced. However, this occured alongside a rise in data imperfections, missing dicharge destiations and inaccurate timestamp documentation. These failures meant that some encounters could not be reliably linked to the correct patient and others had incomplete outcomes, creating risks of misidentification, loss of follow up, impaired safeguarding and infection surveillance, and reduced reliability of diagnostic and admission decisions. | [https://pubmed.ncbi.](https://pubmed.ncbi.nlm.nih.gov/34332657/) [nlm.nih.gov/34332657/](https://pubmed.ncbi.nlm.nih.gov/34332657/)  [https://www.](https://www.sciencedirect.com/science/article/pii/S1931720418304586?casa_token=pONCY2GeKlkAAAAA%3A5slEgxK5KU14_KX6ZHgnDH0d2ogF5UW2O7LLnQnG-4tjPT1j8Mys95rjsmxnGW1wJ07mlYqDSK0q) [sciencedirect.](https://www.sciencedirect.com/science/article/pii/S1931720418304586?casa_token=pONCY2GeKlkAAAAA%3A5slEgxK5KU14_KX6ZHgnDH0d2ogF5UW2O7LLnQnG-4tjPT1j8Mys95rjsmxnGW1wJ07mlYqDSK0q) [com/science/article/pii/S](https://www.sciencedirect.com/science/article/pii/S1931720418304586?casa_token=pONCY2GeKlkAAAAA%3A5slEgxK5KU14_KX6ZHgnDH0d2ogF5UW2O7LLnQnG-4tjPT1j8Mys95rjsmxnGW1wJ07mlYqDSK0q) [1931720418304586?](https://www.sciencedirect.com/science/article/pii/S1931720418304586?casa_token=pONCY2GeKlkAAAAA%3A5slEgxK5KU14_KX6ZHgnDH0d2ogF5UW2O7LLnQnG-4tjPT1j8Mys95rjsmxnGW1wJ07mlYqDSK0q)  [casa_token=pONCY2G](https://www.sciencedirect.com/science/article/pii/S1931720418304586?casa_token=pONCY2GeKlkAAAAA%3A5slEgxK5KU14_KX6ZHgnDH0d2ogF5UW2O7LLnQnG-4tjPT1j8Mys95rjsmxnGW1wJ07mlYqDSK0q) [eKlkAAAAA:](https://www.sciencedirect.com/science/article/pii/S1931720418304586?casa_token=pONCY2GeKlkAAAAA%3A5slEgxK5KU14_KX6ZHgnDH0d2ogF5UW2O7LLnQnG-4tjPT1j8Mys95rjsmxnGW1wJ07mlYqDSK0q) [5slEgxK5KU14_KX6ZH](https://www.sciencedirect.com/science/article/pii/S1931720418304586?casa_token=pONCY2GeKlkAAAAA%3A5slEgxK5KU14_KX6ZHgnDH0d2ogF5UW2O7LLnQnG-4tjPT1j8Mys95rjsmxnGW1wJ07mlYqDSK0q) [gnDH0d2ogF5UW2O7L](https://www.sciencedirect.com/science/article/pii/S1931720418304586?casa_token=pONCY2GeKlkAAAAA%3A5slEgxK5KU14_KX6ZHgnDH0d2ogF5UW2O7LLnQnG-4tjPT1j8Mys95rjsmxnGW1wJ07mlYqDSK0q)  [LnQnG-4tjPT1j8Mys95rjsmxnG](https://www.sciencedirect.com/science/article/pii/S1931720418304586?casa_token=pONCY2GeKlkAAAAA%3A5slEgxK5KU14_KX6ZHgnDH0d2ogF5UW2O7LLnQnG-4tjPT1j8Mys95rjsmxnGW1wJ07mlYqDSK0q) [W1wJ07mlYqDSK0q](https://www.sciencedirect.com/science/article/pii/S1931720418304586?casa_token=pONCY2GeKlkAAAAA%3A5slEgxK5KU14_KX6ZHgnDH0d2ogF5UW2O7LLnQnG-4tjPT1j8Mys95rjsmxnGW1wJ07mlYqDSK0q)  [https://www.cambridge.](https://www.cambridge.org/core/journals/disaster-medicine-and-public-health-preparedness/article/learnings-from-a-national-cyberattack-digital-disaster-during-the-sarscov2-pandemic-in-a-pediatric-emergency-medicine-department/1B36FA7C5C1A8C924F7C88E63FCD8467) [org/core/journals/disast](https://www.cambridge.org/core/journals/disaster-medicine-and-public-health-preparedness/article/learnings-from-a-national-cyberattack-digital-disaster-during-the-sarscov2-pandemic-in-a-pediatric-emergency-medicine-department/1B36FA7C5C1A8C924F7C88E63FCD8467) [er-medicine-and-public-health-preparedness/article/lea](https://www.cambridge.org/core/journals/disaster-medicine-and-public-health-preparedness/article/learnings-from-a-national-cyberattack-digital-disaster-during-the-sarscov2-pandemic-in-a-pediatric-emergency-medicine-department/1B36FA7C5C1A8C924F7C88E63FCD8467) [rnings-from-a-national-cyberattack-digital-disaster-during-the-sarscov2-pandemic-in-a-pediatric-emergency-medicine-department/1B36FA7C5](https://www.cambridge.org/core/journals/disaster-medicine-and-public-health-preparedness/article/learnings-from-a-national-cyberattack-digital-disaster-during-the-sarscov2-pandemic-in-a-pediatric-emergency-medicine-department/1B36FA7C5C1A8C924F7C88E63FCD8467) [C1A8C924F7C88E63F](https://www.cambridge.org/core/journals/disaster-medicine-and-public-health-preparedness/article/learnings-from-a-national-cyberattack-digital-disaster-during-the-sarscov2-pandemic-in-a-pediatric-emergency-medicine-department/1B36FA7C5C1A8C924F7C88E63FCD8467) [CD8467](https://www.cambridge.org/core/journals/disaster-medicine-and-public-health-preparedness/article/learnings-from-a-national-cyberattack-digital-disaster-during-the-sarscov2-pandemic-in-a-pediatric-emergency-medicine-department/1B36FA7C5C1A8C924F7C88E63FCD8467) |
| 23 | Strong et al [90]. | 2022 | October 2021 | Canada | A reflective report from a provincial radiology department describing operational responses and safety adaptations during the 2021 ransomware attack that disable core IT systems in Newfoundland and Labrador. | Cyberattack disruption affected the province-wide healthcare system, serving a geographically dispersed population that relies on radiology for emergency diagnosis, inpatient management, surgical decision making and cancer pathways. | The cyberattack caused the medical IT systems to become inaccessible, removing normal digital ordering, reporting and integrated workflow support. Departments reverted to fully paper-based imaging requests and manual work lists. Preliminary reports were first entered into temporary PACS note fields and later into pop-up alerts so that referrers could still acess actionable findings, while urgent results were communicated verbally. | The loss of automated worklists and electronic identifiers created a high risk of misidentification. For patients, imaging remained availble but at a reduced and prioritised capacity, meaning that non-urgent examinations were deferred, slowing diagnostic and treatment pathways across the system. Manual requesitioning, reporting and tracking lengthened turnaround times, so clinical teams waited longer for imaging to guide decisions. | [https://journals.](https://journals.sagepub.com/doi/full/10.1177/08465371221081549) [sagepub.com/doi/full/10.](https://journals.sagepub.com/doi/full/10.1177/08465371221081549) [1177/084653712210815](https://journals.sagepub.com/doi/full/10.1177/08465371221081549)  [49](https://journals.sagepub.com/doi/full/10.1177/08465371221081549) |
| 24  25 | Hoffman and Baker [49]  Lacobucci [64] | 2023  2024 | May 2021  June 2024 | New Zealand  UK | Reflective analysis of acute orthopaedic service delivery during the 2021 cyberattack on the Waikato District Health Board, a five hospital system that includes New Zealand's only level-1 trauma centre.  A real-time report in the BMJ describing the immediate clinical and operational consequences of the 2024 Synnovis cyberattack on the NHS. The report captures system-wide service disruption and emergency mitigation measures across major London NHS trusts, looking at both acute hospitals and primary care. | The cyberattack affected the entire regional public health netowrk, with the orthopaedic services being responsible for trauma, urgent surgery and elective operations. Patients requiring time-sensitive imaging, operative fixation, spinal surgery, or peri-operative medical optimisation were most at risk, while elective patients experienced significant delays.  The incident affected Guy’s and St Thomas’ (including Evelina London Children’s Hospital and Royal Brompton), King’s College Hospital, and GP services across multiple south-east London boroughs. These were large tertiary centres, hence affecting patients across surgical, transplant, transfusion specialities, outpatients and community diagnostic pathways. | A major ransomware attack on the Waikato District Health Board in New Zealand in 2021, which disabled all computer systems, the internet, printers and hospital phones for close to 4 weeks. The patient management and radiology systems used by the hospital were completely out of operation for 24 days, while the hospital intranet, key for internal communications and dissemination of essential staff information, was offline for 2 months.  The Ransomware attack disabled the pathology IT service 'Syynovis', which formed a vital part of NHS infrastructure, and was usually used to process laboratory samples and match patient blood types. The hospitals declared critical incidents and emergency work was prioritised, with transition to manual processes. | Patients experienced diagnostic and treatment delays due to reduced capacity for laboratory and imaging tests - CT Capacity fell to two scans per hour. Clinicians often lacked prior records or accurate medical histories, creating risks of peri-operative harm (e.g. missing a history of anticoagulation in an elderly patient with a hip fracture). Elective surgery was widely deferred, creating risks of lasting morbidity through disease progression and prolonged pain and disability. Limited intraoperative imaging and delayed access to post-operative investigations increased the risk of wrong-level surgery, missed complications and slow recognition of deterioration.  Patient harms were driven by the loss of timely pathology results and transfusion resources. Hospitals were unable to match patient blood type at the same frequency as normal, leading to postponed operations, diversion of patients and a national appeal for group O blood so that emergency care could continue. | [https://pubmed.ncbi.](https://pubmed.ncbi.nlm.nih.gov/35197299/) [nlm.nih.gov/35197299/](https://pubmed.ncbi.nlm.nih.gov/35197299/)  [https://www.bmj.](https://www.bmj.com/content/385/bmj.q1277.full) [com/content/385/bmj.](https://www.bmj.com/content/385/bmj.q1277.full) [q1277.full](https://www.bmj.com/content/385/bmj.q1277.full) |
| 26  27  28 | Aljaidi et al [91]  Dameff et al [59]  Abouk and Powell [60] | 2022  2023  2024 | May 2017  May 2021  2020 | UK  USA  USA | Quantitative descriptive retrospective observational study using NHS Hospital Episodes Statistics to compare healthcare activity in the weeks befure, during and after the 2017 Wannacry attack in the UK. Supplemented by a technical explaination of the malware and vulnerability.  Retrospective quantitative study using routinely collected operational and clinical registry data to compare ED activity and stroke care performance, across 3 time periods (4 weeks pre-attack, 4 weeks during ransomware attack and 4 weeks post-recovery). Two urban academic EDs within the same regional emergency care network were studied.  Retrospective, quantitative observational study uding a difference-in-differences approach to analyse hospital activity assocaited with ransomware attacks in California between 2014 and 2020. | Acute NHS trusts and GP practicers in England, with greatest operational impact in organisations running unpatched legacy Windows IT Systems or sharing networks with infected sites.  Two large academic emergency departments (combined mean annual census of more than 70,000 care encounters and 11% of San Diego County's total acute inpatient discharges), that were not directly attacked but were geographically adjacent to the target health system. The target health system lost acces to electronic health records, imaging and telemedicine, requiring diversion of ambulances and regional redistribution of emergency patients.  The study examined acute care hospitals in California, distinguishing between those directly experiencing ransomware-related hospital disruption and neighbouring hospitals with the same healthcare markets. The population consisted of patients presenting for emergency and inpatient care at the facilities, allowing an assessment of system-level redistribution of demand rather than outcomes within a single institution. | A self-propagating ransomware worm exploiting the SMBv1 vulnerability (MS17-010) via EternalBlue and installing a DoublePulsar backdoor to enable rapid lateral spread across national health networks. The encryption of endpoints and risk-mitigation shutdowns meant that clinical IT, email, scheduling systems, radiology and some network-connected medical devices became unavailable, forcing a shift to paper-based workflows and service disruption.  A prolonged ransomware attack caused a month-long loss of core digital infrastructure at the target hospital, including loss of the electronic health record and imaging platforms, forcing a shift to paper-based and manual processes.  The study analysed ransomware incidents that disrupted care delivery (as identified in previous cybersecurity datasets), where targetted hospitals experienced operational impairtment that affected clinical workflows. Technical failures were not described in detail, but attacks were characterised by loss of normal digital functionality and service capacity. | At attected trusts there were marked reducations in delivered care, with 6% fewer admissions, 4% fewer ED attendences and 9% fewer elective admissions, alongside widespread cancellation of outpatient appointments and ambulance diversions.  The authors focused on patient impacts at the hospitals neighbouring the hospital which was the victim of the attack, to examine regional effects. At neighbouring hospitals, patients experienced measurable access and timeliness harms, with ED attendances rising by 15%, ambulance arrivals rising by 35% and waiting room time increasing by 48%. Median length of stay for admitted patients increased by ~34%, while the proportion that left without being seen more than doubled and depatures against medical advice increased, indicating a loss of definitive assessment and treatment.  Time-sensitive care was affected, with stroke code activiations rising, reflecting a concentration of high acuity patients at fewer functioning centres.  Victim hospitals experienced short-term reductions in care delivery, with ED visits falling by 8.1% in the first week and 16.2% in the second week after the attack. In contrast, nearby hospitals saw a compensatory increased in ED visits (peaking at 7% by week 3), without a corresponding rise in admissions, demonstrating redistribution of acute demand across the local health system. | [https://ieeexplore.ieee.](https://ieeexplore.ieee.org/document/10050485) [org/document/10050485](https://ieeexplore.ieee.org/document/10050485)  [https://pubmed.ncbi.](https://pubmed.ncbi.nlm.nih.gov/37155166/) [nlm.nih.gov/37155166/](https://pubmed.ncbi.nlm.nih.gov/37155166/)  [https://jamanetwork.](https://jamanetwork.com/journals/jama/fullarticle/2819300) [com/journals/jama/fullart](https://jamanetwork.com/journals/jama/fullarticle/2819300) [icle/2819300](https://jamanetwork.com/journals/jama/fullarticle/2819300) |

| **Article Number** | **Reference** | **Publication Date** | **Cyber Attack Date** | **Country** | **Study Design and Setting** | **Organisation / Population Affected** | **Attack Characteristics and Systems Disrupted** | **Impact on patients and clinical operations** | **URL** |
| --- | --- | --- | --- | --- | --- | --- | --- | --- | --- |
| 29  30 | Pham et al [55]  Chen et al [92] | 2024  2021 | April 2021  2020 | USA  USA | Retrospective quantitative study with a before-during-after comparison, using resuscitation registry and hospital operational data from two academic hospitals located within 1 mile of a four-hospital health system that was disabled by a month long ransomware attack. Cardiac arrest incidence, hospital and ED census, and patient outcomes were analysed across the three phases, using time-series modelling with four years of pre-attack data to determine whether observed case numbers during the attack exceeded the expected seasonal variation.  Single centre qualitative operational case study and recovery-planning analysis based on a ransomware attack affecting a hospital in a large US Tertiary radiology network. | The analysis included all patients with out-of-hospital and in-hospital cardiac arrest, treated at adjacent, non-infected hospital systems, which recieved diverted emergency patients from the compromised network. The attacked system normally accounted for ~25% of regional inpatient discharges, while the study hospitals accounted for ~11%, meaning the latter absorbed a substantial redistribution of time-critical emergency care.  The cyberattack directly affected one hospital in a multi-site referral network, but the operational lessons apply across a horizontally integrated radiology service supporting emergency, inpatient, outpatient, peri-operative, and interventional care. Patients requiring urgent, intra-operative, interventional, and elective imaging were affected through service diversion, cancellation of non-urgent activity, consolidation of imaging to staffed sites, and reliance on analogue workflows. | A month long ransomware attack that caused complete loss of the EHR and core digital systems at a four-hospital organisation, triggering regional ambulance diversion and sustained increases in ED and inpatient census at adjacent hospitals.  Ransomware and defensive network isolation rendered hospital digital infrastructure unusable, including EMR, PACS, RIS, diagnostic workstations, scheduling systems, paging directories, and hospital intranet. Some modalities could still acquire images but without network transmission, forcing at-modality interpretation on non-diagnostic monitors, local CD/DVD image transfer, paper ordering, manual patient identifiers and accession conventions, and telephone/fax result communication. Teleradiology and hub-and-spoke reporting models ceased, smaller sites without on-site radiologists suspended imaging, and the emergency department was initially placed on diversion. | At the neighbouring hospitals the cyberattack was associated with a marked increase in cardiac arrest caseload and substantially worse patient outcomes. Total cardiac arrests rose and exceed time-series forecasts, driven largely by excess out of hospital cases. Survival with favourable neurological outcomes dropped from 38.1% to 5.3% (p<0.001), with the most pronounced deterioration in out-of-hospital cardiac arrest (40.0% to 4.5%, p=0.02).  Patient access to imaging was immediately reduced because elective studies were postponed, some sites stopped scanning, and urgent examinations were consolidated to locations with on-site radiologists, while emergency and interventional cases had to proceed without normal digital safety systems and with limited access to prior imaging or clinical data. Image interpretation at the scanner using non-diagnostic displays and loss of teleradiology slowed diagnosis and increased the risk of communication failure for critical findings. Intra-operative and image-guided procedures were particularly vulnerable because medication dispensing systems, scheduling, and image distribution were simultaneously unavailable. | [https://pubmed.ncbi.](https://pubmed.ncbi.nlm.nih.gov/38605720/) [nlm.nih.gov/38605720/](https://pubmed.ncbi.nlm.nih.gov/38605720/)  [https://pubmed.ncbi.](https://pubmed.ncbi.nlm.nih.gov/34159418/) [nlm.nih.gov/34159418/](https://pubmed.ncbi.nlm.nih.gov/34159418/) |
| 31  32  33  34  35 | Zhao et al [88]  Duffy et al [93]  Stowman and Kalof [94]  Tarka et al [95]  Filipec and Plasil [46] | 2018  2024  2022  2023  2021 | Not Reported  May 2021  October 2020  October 2020  December 2019 | USA  Ireland  USA  USA  Czechia | Qualitative descriptive study using a grounded theory approach to explore impact of the EHR shutdown that occured with a ransomware attack affecting a surgical department. Semi-structured interviews were conducted with general surgery residents who had rotated through the USA hospital during the downtime period.  Quantitative descriptive national cross-sectional survey of laboratories in Ireland examining the impact of the May 2021 HSE cyberattack on laboratory medicine services. A structured electronic questionnaire was distributed to 43 hospital laboratories, achieving a 95% response rate, and reporting numerical measures of service disruption across the pre-analytical, analytical, and post-analytical phases.  Qualitative descriptive single centre case study of a ransomware attack causing a 25-day shutdown of electronic systems in a 620-bed tertiary care hospital in the USA, focusing on the anatomic pathology response and recovery.  A mixed-methods evaluation combining a quantitative retrospective cohort study and a qualitative workflow analysis at a US academic level 1 trauma centre during a 25-day ransomware-related total EMR downtime. The quantitative analysis compared weekday orthopaedic total in-room operative time with week-matched periods in the preceding and following years to quantify loss of surgical capacity, while prospective multidisciplinary staff interviews examined process failures and adaptations in care pathways.  Qualitative organisational case study of a ransomware attack (Emotet - TrickBot - Ryuk) on a regional hospital in the Czechia in December 2019, informed by primary insider experience from the incident response and analysed using a socio-technical cybersecurity framework (Swiss-cheese model). The study provides a chronological reconstruction of the first five days, the recovery phase, and the subsequent system redesign, with emphasis on crisis IT management and governance rather than clinical outcomes. | Hospitalised surgical patients whose care depended on multidisciplinary input, with residents, nurses, and allied professionals adapting their communication practices to maintain continuity and safety in the absence of the EHR.  Hospital inpatients, outpatients, and primary care patients whose clinical management depended on access to laboratory testing and timely communication of results within the national health system.  Patients requiring pathological (histopathological and cytological) diagnosis, including cancer resections, intraoperative consultations, and other time-critical tissue diagnoses that guide treatment decisions across the regional referral network.  Patients requiring urgent and elective orthopaedic surgery, especially those dependent on coordinated peri-operative processes such as imaging, laboratory testing, medication reconciliation, operating room scheduling, and postoperative bed management.  A 411 bed general hospital serving a regional population (up to ~400,000 seasonally). The affected population from a patient harm perspective were individuals requiring acute and routine hospital care during the period of severe service restriction, inclusing emergency presentations, inpatients, and patients needing diagnostics, laboratory services, imaging, prescribing, and transfusion support. | The cyberattack resulted in a complete shutdown of the electronic health record, eliminating electronic documentation, order entry, and asynchronous messaging. All clinical information management reverted to paper charts and direct verbal updates, removing usual digital communication pathways.  The ransomware attack caused shutdown of the national healthcare network, with loss of laboratory systems, digital requesting and reporting, email, and in some sites telecommunications. Over half of laboratories had no functioning LIS for prolonged periods, requiring full manual workflows for specimen reception, tracking, analysis, and reporting, with loss of automated reference ranges, alert flags, historical results, and electronic transmission of results.  The cyberattack resulted in complete loss of the EHR, laboratory information system, internet and intranet, email, dictation software, printers, PACS, scheduling systems, billing services, and networked telephony. Specimen accessioning, tracking, slide printing, reporting, and access to prior patient results were all disabled, forcing a fully manual workflow and necessitating diversion of large specimen volumes to external laboratories.  The ransomware attack destroyed the core network infrastructure, disabling over 5000 computers and causing complete loss of the EMR, imaging access, electronic scheduling, laboratory order entry, intranet/internet, phones, paging, and centralised patient-tracking systems. Restoration required rebuilding the software environment before recovery from backups, leading to prolonged total downtime.  A multi stage ransomware intrusion delivered via a phishing campaign, with Emotet enabling lateral movement, TrickBot harvesting credentials and escalating privileges, and Ryuk performing network-wide encryption. Core clinical and administrative systems became inoperable, including the hospital information system, domain services, email, laboratory and prescribing platforms, and most networked workstations. The hospital was forced into full paper-based operation, with physical network disconnection, complete rebuild of the domain in a clean environment, mass reinstallation of end-user devices, and staged restoration from verified backups. | Patient care became more bedside-centred during the attack, with clinicians relying on repeated face-to-face discussions to clarify plans, confirm orders, and hand over information. This increased explicit, deliberate communication and improved shared understanding of care plans, which residents perceived as supporting more patient-centred decision making and reducing ambiguity. However, the loss of real-time electronic records required manual tracking of clinical information and created potential risks for delays or information gaps if in-person communication did not occur, making continuity of care more labour-intensive and dependent on team availability.  Diagnostic activity decreased markedly, with most laboratories reporting substantial reductions in test requests, particularly from primary care, suggesting deferred or missed investigations. Manual processing and transcription increased workload, turnaround times, and risk of identification and reporting errors, while lack of previous results and decision-support functions reduced the clinical reliability of testing for patient management. Communication failures prevented many laboratories from maintaining critical-value notification pathways, delaying clinical decision-making and treatment.  During the cyberattack the diagnostic throughput fell to a fraction of normal capacity while staffing requirements increased because all specimen processing, documentation, and reporting became manual. Inability to accession and track specimens created a growing backlog and risk to specimen integrity and result accuracy, and absence of prior records limited diagnostic context. Urgent results had to be communicated by telephone, delaying definitive reporting and treatment planning. Prolonged downtime required major workforce redeployment and introduced sustained delays in diagnosis, retrospective data entry, and billing reconciliation, with potential consequences for timely clinical decision-making and continuity of care.  The departmental operative capacity fell by ~53%, restricting activity largely to urgent cases and delaying elective surgery. Core care processes became manual and fragmented: patient location tracking, medication reconciliation, laboratory testing, and order execution were slow and inconsistent, and intraoperative decisions were made without real-time imaging. Early patient diversion to neighbouring hospitals redistributed demand across the region. For time-critical trauma patients, these disruptions meant delayed optimisation and surgery, with care remaining labour-intensive and less efficient despite the gradual development of paper-based workarounds.  Authors report that the hospital was effectively non-functional for approximately one week, with emergency services diverted and routine care cancelled or postponed. Diagnostic capacity, laboratory processing, electronic prescribing, and blood bank functionality were temporarily unavailable, requiring external procurement of blood products and reliance on manual communication of urgent results, with significant implications for patient management. Care delivery became significantly slower and more labour-intensive, limiting throughput and creating regional redistribution of patients. | [https://www.](https://www.sciencedirect.com/science/article/pii/S1072751518308019) [sciencedirect.](https://www.sciencedirect.com/science/article/pii/S1072751518308019) [com/science/article/pii/S](https://www.sciencedirect.com/science/article/pii/S1072751518308019) [1072751518308019](https://www.sciencedirect.com/science/article/pii/S1072751518308019)  [https://pubmed.ncbi.](https://pubmed.ncbi.nlm.nih.gov/37737914/) [nlm.nih.gov/37737914/](https://pubmed.ncbi.nlm.nih.gov/37737914/)  [https://journals.lww.](https://journals.lww.com/pathologycasereviews/pages/articleviewer.aspx?year=2022&issue=07000&article=00009&type=Fulltext) [com/pathologycaserevie](https://journals.lww.com/pathologycasereviews/pages/articleviewer.aspx?year=2022&issue=07000&article=00009&type=Fulltext) [ws/pages/articleviewer.](https://journals.lww.com/pathologycasereviews/pages/articleviewer.aspx?year=2022&issue=07000&article=00009&type=Fulltext) [aspx?](https://journals.lww.com/pathologycasereviews/pages/articleviewer.aspx?year=2022&issue=07000&article=00009&type=Fulltext) [year=2022&issue=0700](https://journals.lww.com/pathologycasereviews/pages/articleviewer.aspx?year=2022&issue=07000&article=00009&type=Fulltext) [0&article=00009&type=](https://journals.lww.com/pathologycasereviews/pages/articleviewer.aspx?year=2022&issue=07000&article=00009&type=Fulltext) [Fulltext](https://journals.lww.com/pathologycasereviews/pages/articleviewer.aspx?year=2022&issue=07000&article=00009&type=Fulltext)  [https://pubmed.ncbi.](https://pubmed.ncbi.nlm.nih.gov/36801172/) [nlm.nih.gov/36801172/](https://pubmed.ncbi.nlm.nih.gov/36801172/)  [https://doi.org/10.](https://doi.org/10.3849/1802-7199.21.2021.01.027-052) [3849/1802-](https://doi.org/10.3849/1802-7199.21.2021.01.027-052)  [7199.21.2021.01.027-](https://doi.org/10.3849/1802-7199.21.2021.01.027-052)  [052](https://doi.org/10.3849/1802-7199.21.2021.01.027-052) |

| **Article Number** | **Reference** | **Publication Date** | **Cyber Attack Date** | **Country** | **Study Design and Setting** | **Organisation / Population Affected** | **Attack Characteristics and Systems Disrupted** | **Impact on patients and clinical operations** | **URL** |
| --- | --- | --- | --- | --- | --- | --- | --- | --- | --- |
| 36  37  38  39 | Slattery et al [96]  Oliver et al [45]  Harvey et al [74]  Russell et al [97]. | 2022  2022  2023  2023 | May 2021  Jan 2019  May 2021  May 2021 | Ireland  Canada  Ireland  Ireland | Quantitative observational study conducted in a hospital in Ireland examining outpatient clinic attendance across three defined time periods: pre–text reminder system, pre-cyber-attack with reminders active, and post-cyber-attack when the reminder system was disabled. Statistical comparison assessed the effect of the national HSE ransomware attack on non-attendance rates in ENT outpatient services.  Quantitative descriptive operational case study (single-centre incident report) from an academic radiation oncology service in Canada, detailing a ransomware network outage, the emergency clinical workaround used to continue treatment without the oncology information system (OIS), and the development of a structured contingency preparedness framework.  A quantitative descriptive multicentre observational study based on a structured questionnaire distributed to cancer clinical trials units within a national trials network, with comparative analysis of key performance indicators across three defined time periods (four weeks before, during, and after the May 2021 ransomware attack on the Irish public health system).  The survey data were supplemented by contemporaneous organisational records from weekly national coordination teleconferences to contextualise operational impact and recovery.  A mixed-methods descriptive study examining the impact of the 2021 national ransomware attack on the Irish National Orthopaedic Register across all nine participating hospitals. Quantitative data from local institutional records and the central registry were used to measure downtime, case capture, adverse events, and the staff time and costs required for data reconciliation. In addition, structured interviews with specialists provided qualitative data on workflow disruption, resource burden, patient flow, and service effects. Descriptive statistical analysis was undertaken. | The study focused on a tertiary referral ENT outpatient department with multiple subspecialty clinics. The affected patient population were individuals scheduled for face-to-face otolaryngology outpatient assessment, many on long waiting lists and requiring in-person examination with specialist equipment for diagnosis of conditions such as head and neck cancer, otology, rhinology, and skull-base disease.  A radiation oncology department treating  >2000 patients annually across a main centre and affiliated unit. The affected patient population were individuals actively receiving radiotherapy, particularly those with time-critical malignancies (e.g., head and neck, cervix, lung), for whom treatment interruption risks tumour repopulation, loss of local control, and worse survival.  The affected organisations were cancer clinical trials units delivering investigational systemic therapy and radiotherapy studies to patients in acute hospitals. The population impacted were patients with cancer referred for trial screening, undergoing trial treatment, or requiring  protocol-mandated diagnostics, follow-up, and safety monitoring.  The affected system was a national arthroplasty registry capturing patients undergoing primary and revision hip and knee replacement surgery in public elective hospitals and one private institution. The population relevant to harm comprised surgical patients whose perioperative implant history, operative data, and follow-up outcomes are required for safe revision surgery, surveillance, and service planning. | The national cyberattack disabled core health IT infrastructure, including patient administration and automated SMS appointment reminder systems. Although the study focuses on one department, the disruption was system-wide and also affected laboratory, radiology, and scheduling platforms, increasing administrative burden and removing digital communication tools used to coordinate outpatient care.  A zero day malware infection forced shutdown of hospital networks and encrypted multiple clinical systems, including the oncology information system (OIS) and hospital IT system. Network isolation also severed connectivity to the satellite centre. Core radiotherapy workflows dependent on digital integration - treatment records, scheduling, verification, and communication - were immediately unavailable.  Conti ransomware attack on the national health service led to shutdown of all connected systems, with loss of electronic health records, radiology and laboratory information systems, imaging distribution, email, scheduling platforms, telemedicine, and secure inter-site communication. Community diagnostic pathways and primary care messaging were also disabled.  A Conti ransomware attack that caused disconnection of approximately 70,000 devices and shutdown of national health IT infrastructure hosting the central registry database. Hospitals lost access to the online registry for a median of 134 days, with only intermittent download capability after approximately eight weeks. | Loss of the reminder system was associated with a statistically significant rise in non-attendance from 13.0% pre-attack to 16.1% post-attack (p = 0.002), equating to 32 additional missed appointments in two weeks that required rebooking into already over-subscribed clinics. These missed visits risk delayed diagnosis and treatment in a specialty where timely in-person assessment is essential and waiting lists are prolonged.  The radiotherapy delivery was at risk of clinically unsafe delays; treatment gaps beyond accepted thresholds can reduce cure rates in several cancers. Workflows became labour-intensive, requiring manual charting, duplicated data verification, extended treatment times, staff redeployment, and multiple daily in-person command briefings. Although no fractionation errors occurred, the event exposed major safety vulnerabilities: uncertainty about patient treatment status, dependence on a single digital record-and-verify system, and lack of predefined downtime procedures. The incident produced substantial staff workload and stress and highlighted the potential for treatment delays, incorrect dosing, or missed therapy in the absence of rapid contingency adaptation.  Negative impacts on cancer patients were noted through multiple systems and services. There was an 85% reduction in trial referrals and a 55% reduction in recruitment during the four weeks following the attack, with prolonged disruption to protocol delivery and surveillance. Inability to access historical imaging and laboratory results prevented assessment of treatment response and eligibility, delaying or excluding patients from trials and risking deviation from time-sensitive treatment schedules. Radiotherapy trials were particularly affected because interruption of fractionated treatment risks loss of tumour control, while surgical and diagnostic delays compounded existing waiting times. Recovery of trial activity was heterogeneous and in some centres diagnostic reporting delays persisted for many months, demonstrating sustained patient risk linked to loss of digital infrastructure and lack of preparedness.  Patients experienced indirect harm through cancelled or postponed operations and disrupted clinic flow, while operating room efficiency fell because perioperative documentation reverted to paper. The prolonged downtime generated 2850 additional staff hours for manual data reconciliation at an estimated cost of USD 181,000 - 216,000, diverting clinical personnel from routine care and extending recovery of normal services despite preservation of registry data integrity. | [https://pmc.ncbi.nlm.nih.](https://pmc.ncbi.nlm.nih.gov/articles/PMC9380845/) [gov/articles/PMC93808](https://pmc.ncbi.nlm.nih.gov/articles/PMC9380845/) [45/](https://pmc.ncbi.nlm.nih.gov/articles/PMC9380845/)  [https://www.](https://www.sciencedirect.com/science/article/pii/S2452109422000033) [sciencedirect.](https://www.sciencedirect.com/science/article/pii/S2452109422000033) [com/science/article/pii/S](https://www.sciencedirect.com/science/article/pii/S2452109422000033) [2452109422000033](https://www.sciencedirect.com/science/article/pii/S2452109422000033)  [https://pmc.ncbi.nlm.nih.](https://pmc.ncbi.nlm.nih.gov/articles/PMC10281450/) [gov/articles/PMC10281](https://pmc.ncbi.nlm.nih.gov/articles/PMC10281450/) [450/](https://pmc.ncbi.nlm.nih.gov/articles/PMC10281450/)  [https://pmc.ncbi.nlm.nih.](https://pmc.ncbi.nlm.nih.gov/articles/PMC10427069/) [gov/articles/PMC10427](https://pmc.ncbi.nlm.nih.gov/articles/PMC10427069/) [069/](https://pmc.ncbi.nlm.nih.gov/articles/PMC10427069/) |
| 40  41  42 | Kelly et al [54]  Feeley et al [61]  Neprash et al [56] | 2023  2022  2024 | April 2017  May 2021  2016 | USA  Ireland  USA | Single centre retrospective descriptive study reporting trauma outcomes at a ransomware related EHR downtime at a Level 1 trauma centre. The article primarily provides expert commentary and preparedness recommendations, with the quantitative outcome comparison used illustratively rather than as a formally designed analytical study.  A qualitative descriptive service evaluation of orthopaedic trauma service delivery during the 2021 national ransomware attack on the Irish Health Service Executive.  Challenges and workflow adaptations were discussed at regional level and collated across multiple regional trauma units and specialist centres to identify effective practice changes.  Quantitative observational study using an event-study design with linked ransomware-attack data, American Hospital Association survey data, and Medicare fee-for-service claims (hospital-week level). | The ransomware attack affected a tertiary Level 1 trauma centre serving a large regional catchment area and reliant on integrated electronic systems for emergency, operative, and critical care coordination. The population of interest for outcome assessment was trauma patients admitted during the downtime period. The organisational disruption extended across the hospital network, with more than 6000 computers disabled and loss of the electronic medical record, PACS imaging access, and electronic interdepartmental communication.  A ransomware attack that affected the Irish public healthcare system and, specifically, regional orthopaedic trauma referral networks. The population impacted were trauma and elective orthopaedic patients whose assessment and treatment depend on timely access to imaging, laboratory results, referral information, and preoperative planning tools. Loss of national systems disrupted interhospital referrals, outpatient review, waiting-list management, and operative scheduling, with patients experiencing deferral of elective care and the need for additional face-to-face assessments to replace virtual pathways.  Medicare patients using rural and urban hospitals that were victim to ransomware (inpatient, outpatient, and emergency care), with additional system-level impacts on hospital finances and access to alternative hospitals. The study compared 43 rural and 117 urban hospitals that experienced ransomware attacks from 2016 through 2021. | A SamSam ransomware attack that infiltrated the hospital network through brute-force access to a web server, encrypted clinical data, and rendered core systems inoperable for approximately six to eight weeks. Unlike other attacks such as WannaCry or with phishing, SamSam generated complex password combinations to access the server and infected other computers until the program had saturated the network. The electronic health record, picture archiving and communication system, and digital communication platforms were unavailable, forcing a complete reversion to paper documentation and manual ordering processes.  The cyberattack resulted in a national shutdown of electronic health records, the national imaging platform, laboratory systems, email, online referral pathways, and dictation services. To prevent viral spread across interconnected regional servers, shared systems were taken offline, eliminating remote access to historical and external imaging and laboratory data. Digital templating software for arthroplasty and access to prior operative records were unavailable, and hospitals reverted to paper documentation, physical image transfer, portable imaging in outpatient settings, and secure messaging applications for inter-site communication.  Ransomware caused sharp but temporary activity reductions in the first week: inpatient admissions fell ~15–17%, outpatient visits ~22–35%, and ED visits ~10–19%, with parallel Medicare revenue losses (~21–28%), recovering within 2–3 weeks. For patients, disrupted services meant forgone or displaced care; rural patients faced substantially longer travel to the nearest functioning hospital (median 33 min/26.8 miles vs 9 min/3.5 miles urban), increasing time-to-treatment for emergencies and risk of delayed routine care. Financial shocks posed additional threats to rural hospital viability, with potential downstream loss of local access to care. | Operative volume decreased and both ICU and total hospital length of stay were slightly longer during the downtime. Care delivery became entirely paper-based, requiring substantial workflow adaptation and increasing operational burden, and the prolonged loss of digital systems created latent safety risks and inefficiencies that were not fully captured by the available outcome metrics. The report emphasises that maintenance of care quality depended on pre-existing downtime protocols, structured communication, and rapid access to alternative data sources.  Loss of the EHR, national imaging and laboratory systems limited access to prior imaging, implant details, operative history, and baseline bloods, constraining safe clinical decision-making. The unavailability of digital templating for arthroplasty prevented accurate preoperative planning, so elective joint replacements were reduced or stopped to avoid implant mismatch, intraoperative delays, and complications. For trauma patients, inability to view external radiology eliminated virtual triage, requiring in-person reassessment and repeat imaging, which delayed definitive management. Loss of historical notes and e-referrals led to deferral of elective clinics and waiting-list progression because of risks such as wrong-site surgery or incorrect prioritisation.  The ransomware attacks produced reductions in inpatient, outpatient, and emergency activity, indicating cancelled or deferred care that could delay diagnosis and treatment, allowing progression of acute and chronic conditions.  Emergency department disruption required patients to travel substantially farther to the nearest functioning hospital in rural areas, potentially creating time-critical delays for acute conditions. Declines in outpatient activity suggest missed surveillance, follow-up, and routine management, with potential  downstream deterioration and higher-acuity presentation. | [https://www.](https://www.injuryjournal.com/article/S0020-1383(23)00750-7/fulltext) [injuryjournal.](https://www.injuryjournal.com/article/S0020-1383(23)00750-7/fulltext) [com/article/S0020-1383](https://www.injuryjournal.com/article/S0020-1383(23)00750-7/fulltext) [(23)00750-7/fulltext](https://www.injuryjournal.com/article/S0020-1383(23)00750-7/fulltext)  [https://pubmed.ncbi.](https://pubmed.ncbi.nlm.nih.gov/34782238/) [nlm.nih.gov/34782238/](https://pubmed.ncbi.nlm.nih.gov/34782238/)  [https://pubmed.ncbi.](https://pubmed.ncbi.nlm.nih.gov/38494590/) [nlm.nih.gov/38494590/](https://pubmed.ncbi.nlm.nih.gov/38494590/) |

| **Article Number** | **Reference** | **Publication Date** | **Cyber Attack Date** | **Country** | **Study Design and Setting** | **Organisation / Population Affected** | **Attack Characteristics and Systems Disrupted** | **Impact on patients and clinical operations** | **URL** |
| --- | --- | --- | --- | --- | --- | --- | --- | --- | --- |
| 43  44  45 | Abbou et al [13]  Mashinchi et al [98]  Gabbay‐Benziv et al [76] | 2024  2024  2023 | October 2021  May 2021  October 2021 | Israel  Ireland  Israel | Quantitative retrospective observational case study of a ransomware attack, using routinely collected administrative time-series data to compare clinical activity during the 8 week postattack period with the same weeks in 2019 to control for seasonality and COVID19 effects. The study was conducted in a 546-bed public university teaching hospital that provides secondary and tertiary services to a regional population of approximately 500,000.  A descriptive case study of the Conti ransomware attack on Ireland’s national public health system, drawing on post-incident review material, national reports, and operational timelines to analyse preparedness, incident response, and recovery. The setting was the Health Service Executive (HSE), a highly distributed, centrally governed national health service comprising 54 acute hospitals, community and mental health services, primary care, and the national ambulance service.  A retrospective quantitative descriptive study of a ransomware attack’s impact on a single obstetrics and gynecology division in a mid-sized hospital. Activity during the 2 weeks before and 8 weeks after the attack (11 weeks total) was compared with the equivalent pre-pandemic period, alongside a narrative description of operational adaptations used to maintain maternity care and restore services. | The cyberattack affected all hospital services because the organisation was fully digital and paperless, with computerised physician order entry, EMR, laboratory, imaging (RIS/PACS), admission and transfer systems, operating theatre management, outpatient scheduling, internal communication, and links to national community records all rendered inaccessible. As a result, the population impacted comprised all patients who would normally receive emergency care, inpatient treatment, surgery, diagnostic testing, outpatient review, or obstetric services at the hospital.  The disruption affected patients across the full continuum of care, including acute hospital inpatients, emergency department attendees, or elective procedures, community and primary-care patients, and those dependent on national screening, mental health, and social-care services. The need to shut down the national network to contain the attack removed access to core clinical systems across multiple hospitals simultaneously, meaning that large numbers of patients experienced cancelled appointments, deferred investigations and treatment, and reliance on manual workflows across geographically dispersed services.  Study population included pregnant women and postpartum patients requiring emergency, elective, inpatient, and ambulatory obstetric care. This included women presenting in labour, those scheduled for cesarean delivery or elective procedures, high-risk pregnancies requiring surveillance, and patients attending obstetric emergency or outpatient clinics whose care pathways depend on timely monitoring, imaging, laboratory results, and coordinated multidisciplinary decision-making. | A DeepBlueMagic ransomware attack that encrypted the hospital’s network and forced immediate disconnection from the internet, producing total loss of access to clinical and administrative systems. Staff could not view ward patient lists, prior medical histories, scheduled appointments, laboratory results, imaging, or internal email and intranet communication. Core digital infrastructure had to be rebuilt on a new network with phased restoration, with laboratory functionality returning in week 1, radiology archiving in week 2, the admission and transfer system shortly after, the full EMR in week 4, and internal communications only after 8 weeks.  The ransomware obtained initial access through a phishing email months before the event, followed by covert lateral movement using privileged accounts across a flat, poorly segmented national network until the Conti ransomware was executed in May 2021, encrypting thousands of servers and endpoints and forcing the disconnection of all internal and external connectivity. This resulted in loss of national and local EMRs, diagnostic and administrative applications, email and digital communication, and many clinical support systems.  The ransomware attack disabled all computerized hospital functions, including the obstetric EHR, centralised fetal heart rate monitoring and managerial dashboard, laboratory and blood-bank systems, imaging access, appointment lists, telehealth for high risk pregnancies, neonatal - maternal record integration, and internal communication. | Clinical activity fell immediately, with sharp reductions in emergency attendances, hospital admissions, bed occupancy, surgical procedures, and ambulatory imaging, while elective operations and non-urgent admissions were cancelled and some patients were diverted or referred elsewhere. Disruptions created risks of delayed diagnosis and treatment, particularly for patients whose care depended on imaging, laboratory monitoring, multidisciplinary coordination, or blood products, as transfusions were minimised because the loss of historical blood-group data increased misidentification risk. The absence of prior records meant clinicians made decisions with incomplete information, increasing the potential for duplicated tests, medication errors, and conservative management or deferral of complex cases. Manual specimen labeling and phone reporting of results introduced additional opportunities for transcription and identification error, and the inability to access scheduled outpatient lists meant some patients were effectively lost to planned follow-up.  The ransomware attack caused immediate loss of digital infrastructure that caused widespread cancellation and postponement of outpatient activity, diagnostics, and elective care, diversion and rescheduling of services, and major constraints on clinical coordination across hospital and community settings. At system level, the scale and duration of application outages meant that access to care was reduced for large patient populations simultaneously, demonstrating how loss of shared national infrastructure translated into delayed care, reduced service capacity, and sustained operational backlogs even after technical decryption began.  Departmental activity fell by ~5–10%, with a prolonged reduction in admissions and occupancy (≈50% early after the attack) and a ~30% short-term drop in high-risk ambulatory visits, indicating disrupted surveillance for complex pregnancies. Loss of longitudinal records and prenatal data meant clinical decisions relied on patient recall, increasing risk of delayed diagnosis, suboptimal risk stratification, and laboratory or transfusion error, which in turn drove conservative management and inter-hospital transfers.  Absence of centralised fetal monitoring removed remote oversight of labour, requiring frequent bedside review and reducing monitoring capacity. Suspension of most elective activity and redirection of complex cases preserved intrapartum safety but reduced service accessibility, continuity of antenatal care, and local capacity for high-risk obstetric  management. | [https://pubmed.ncbi.](https://pubmed.ncbi.nlm.nih.gov/38433989/) [nlm.nih.gov/38433989/](https://pubmed.ncbi.nlm.nih.gov/38433989/)  [https://journals.](https://journals.sagepub.com/doi/full/10.1177/20438869241279443) [sagepub.com/doi/full/10.](https://journals.sagepub.com/doi/full/10.1177/20438869241279443) [1177/204388692412794](https://journals.sagepub.com/doi/full/10.1177/20438869241279443)  [43](https://journals.sagepub.com/doi/full/10.1177/20438869241279443)  [https://pubmed.ncbi.](https://pubmed.ncbi.nlm.nih.gov/36740900/) [nlm.nih.gov/36740900/](https://pubmed.ncbi.nlm.nih.gov/36740900/) |
| 46  47 | Looi et al [52]  Chen et al [99] | 2025  2025 | September 2020  2017 | Finland  USA | A qualitative descriptive case study of the Vastaamo psychotherapy electronic health-record data breach, based on detailed media and legal reporting. The paper analyses organisational, technical, and regulatory failures and derives practice lessons for mental health services.  Quantitative study using a propensity-score–matched difference-in-difference design with event-study and regression-discontinuity analyses. The study linked U.  S. Department of Health and Human Services hospital data-breach reports to 1,295,537 patient-level inpatient admissions from emergency departments in Florida (2013–2017) across 12 matched hospitals to estimate short- and long-term effects on  clinical operations and mortality. | Approximately 30,000 psychotherapy patients whose highly sensitive therapy records and personal identifiers were exfiltrated and publicly released, with subsequent direct extortion of individual patients. The affected population consisted largely of people with mental illness receiving psychological therapy, a group with heightened vulnerability to confidentiality breaches.  Six medicare certified acute hospitals that experienced large data breaches and their emergency admissions population, including high-risk subgroups such as acute myocardial infarction (AMI), heart failure, and stroke patients. | The hacker targetted the electronic health record database of the Vastaamo private psychotherapy provider, where weak cybersecurity governance and security controls (lack of encryption at rest and in transit, poor password practices, no multifactor authentication, firewall and remote-access failures) enabled large-scale data exfiltration. The incident involved staged leaks followed by full database release and direct patient blackmail.  Large protected-health-information data breaches that triggered federal investigation, multi-year regulatory monitoring, staff training, policy revision, and substantial health-information-technology upgrades, particularly expansion of health-information-exchange capability and electronic information flow between providers. | The primary patient harms were psychological and social rather than service-delivery disruption. Exposure of detailed psychotherapy narratives created severe distress, shame, fear of disclosure, and ongoing vulnerability, with reported suicides and extensive legal and financial consequences for patients and families. The breach undermined trust in mental health services, threatened care engagement and disclosure in therapy, and created long-term confidentiality risks because leaked data are permanent and individually identifiable. Direct patient extortion represents a distinct mechanism of harm compared with operational ransomware, producing sustained mental health deterioration independent of healthcare access.  There was no reported significant short-term deterioration in care quality was detected around the breach event, but over time patients experienced faster care processes, with ED-to-admission time reduced by ~0.56 hours (~10%) and time to principal procedure reduced by ~0.18 days (~8%), alongside a small reduction in length of stay. AMI patients had a clinically meaningful 1-percentage-point absolute reduction in in-hospital mortality, while mortality for other groups was unchanged.  These patient benefits are interpreted as downstream effects of post-breach remediation that improved information availability, inter-provider communication, and care  coordination rather than direct effects of the breach itself. | [https://pubmed.ncbi.](https://pubmed.ncbi.nlm.nih.gov/39400039/) [nlm.nih.gov/39400039/](https://pubmed.ncbi.nlm.nih.gov/39400039/)  [https://pubmed.ncbi.](https://pubmed.ncbi.nlm.nih.gov/39789912/) [nlm.nih.](https://pubmed.ncbi.nlm.nih.gov/39789912/) [gov/39789912/](https://pubmed.ncbi.nlm.nih.gov/39789912/) |
| 48 | Ades et al [51] | 2021 | 2020 | USA | A mixed-methods descriptive case study of a medical and haematological oncology service during a month-long ransomware related network outage. Quantitative activity data (weekly visits and infusion volumes) were reported alongside a narrative operational account and a formal ethical rationing framework. | The University of Vermont Health Network (UVMHN) medical oncology and haematology service, including outpatient infusion, inpatient oncology and patient referral pathways.  Affected populations included patients already recieving chemotherapy, patients with acute haematological conditions, stem-cell transplant recipients, neuro-oncology patients and patients with newly diagnosed cancer requiring  urgent staging and treatment. | Ransomware attack causing total loss of all network intranet servers, email communications and clinical systems (EMR, pharmacy information systems, laboratory, pathology and radiology). Restoration of EMR took 25 days, secure email took 28 days and radiology viewing systems took 40 days. More than 5000 network computers were infected and all servers required wiping and rebuilding, necessitating assistance from the Vermont National Guard. | Outpatient oncology volume fell by 41% overall, with infusion centre visits dropping by 63% in the first week before partial recovery. New patient visits fell 39%. Loss of EMR-embedded chemotherapy plan templates and electronic safety checks (allergy verification, dose calculation) removed automated safeguards from systemic therapy delivery, raising the risk of wrong-drug, wrong-dose and missed dose events. Laboratory delays created critical bottlenecks for chemotherapy-dependent monitoring (e.g high-dose methotrexate protocols). Stem-cell transplant initiation was delated. Clinical trial admissions were halted and protocol deviations occured dur to inability to access radiological imaging within required windows. | [https://pmc.ncbi.nlm.](https://pmc.ncbi.nlm.nih.gov/articles/PMC8758119/) [nih.](https://pmc.ncbi.nlm.nih.gov/articles/PMC8758119/) [gov/articles/PMC875](https://pmc.ncbi.nlm.nih.gov/articles/PMC8758119/) [8119/](https://pmc.ncbi.nlm.nih.gov/articles/PMC8758119/) |

| **Article Number** | **Reference** | **Publication Date** | **Cyber Attack Date** | **Country** | **Study Design and Setting** | **Organisation / Population Affected** | **Attack Characteristics and Systems Disrupted** | **Impact on patients and clinical operations** | **URL** |
| --- | --- | --- | --- | --- | --- | --- | --- | --- | --- |
| 49  50 | Lippi and Ferrari [58]  Choudhuri et al [66] | 2024  2026 | 2023  2022 | Italy  India | Editorial commentary and operational case report from the Laboratory Medical Service of an Italian University hospital following a ransomware attack taht caused completed IT system failure. The paper describes the activiation of a predefined emergency recovery plan and operational adaptations across two central laboratories serving  ~1200 hospital beds, two emergency rooms, intensive care units and a trauma centre.  A viewpoint article summarising published evidence and expert opinion regarding cyber-resilience in intensive care units (ICUs) in India, using the 2022 AIIMS Dehli ransomware attack as a clinical example and proposing a resource stratified preparedness framework. | University Hospital of Verona, Italy. Patients affected included all hospital inpatients, emergency department patients, and ICU patients across two hospital sites whose clinical management depended on lab testing during the ~72-hour period of system outage. The lab usually performs ~800 types of tests, during the attack this was restricted to a panel of ~20 urgent investigations.  ICUs within Indian hospitals, with a focus on critically ill patients and multidiscplinary ICU teams (physicians, nurses, pharmacists and allied healthcare professionials). The AIIMS Dehli hospital is presented as a principal example of a healthcare organisation affected by ransomware. | A ransomware attack launched at 2230 on 22nd October 2023, encrypting files across most hospital servers. The hospital information system (HIS), laboratory information system (LIS), order entry, result reporting, intranet and all laboratory instrument connectivity were immediately lost. Manusal programming was the only means for operating the lab analysers.  The November 2022 ransomware attack on the All India Institute of Medical Sciences (AIIMS) Delhi compromised approximately 50 servers, rendered the hospital information system inaccessible for over 2 weeks, and forced reversion to paper-based records across inpatient, outpatient, and laboratory services. | All routine labs were suspended for the full duration of the outage, with testing restricted to ~20 urgent investigations. Request reverted to handwritten emergency mnodules, samples were manually labelled, transported and tracked. Results were transmitted exclusively by fax, causing significant bottle necks nd result delays. The loss of electronic orgering and result display in wards removed automated reference ranges, alerts and clinical decision support safeguards - raising risk of errors. Manual transcription of results introduced the risk of identification and reporting errors. Non-urgent investigations were cancelled or postpones, with implications for monitoring chronic disease patients and planned diagnostic pathways.  The paper synthesises evidence on how cyberattacks increase medication errors, causes delays in laboratory results, impaiirs communicatoins, disrupts critical care workflows and clinical decision support. | [https://jlpm.](https://jlpm.amegroups.org/article/view/8586/html) [amegroups.](https://jlpm.amegroups.org/article/view/8586/html) [org/article/view/8586/](https://jlpm.amegroups.org/article/view/8586/html) [html](https://jlpm.amegroups.org/article/view/8586/html)  [https://www.ijccm.](https://www.ijccm.org/doi/IJCCM/pdf/10.5005/jp-journals-10071-25203) [org/doi/IJCCM/pdf/10.](https://www.ijccm.org/doi/IJCCM/pdf/10.5005/jp-journals-10071-25203) [5005/jp-journals-10071-25203](https://www.ijccm.org/doi/IJCCM/pdf/10.5005/jp-journals-10071-25203) |
| 51 | Neprash et al [62] | 2026 | 2016-2021 | USA | A retrospective quasi-experimental observational study using a database of hospital ransomware attacks linked to Medicare claims data. A stacked event-study and difference-in-differences design was used to estimate causal effects of ransomware attacks on hospital operations and patient outcomes. | 160 hospitals in the USA affected by 74 ransomware attacks between 2016 and 2021. The study looked at Medicare fee-for-service beneficiaries recieving emergency inpatient and outpatient are, with comparisons to matched non-attacked hospitals and hospitals attacked later in study period. | Cyberattacks (predominantly ransomware) disrupted hospital electronic health records, imaging services, medication dispensing systems and other digital infrastructure.  Operational consequencces included system downtime, cancalled surgeries and  appointments, ambulance diversions and reduce clinical capacity. | Hospital activity fell significantly during the first week of a cyberattack, with emergency, inpatient and outpatient volumes decreasing by 17-24%. Patients already hospitalised when attacks begain, experienced a 34-38% increase in in-hospital mortaility. Mortality effects were greater during severe attacks involving ambulance diversion or cancelled surgerios, among ICU patients and those with multiple chronic conditions. |  |
| 52 | Davids et al [79] | 2026 | 2024 | South Africa | A cross-sectional survery conducted between September to December 2024at Tygerberg Hospital, a 1384-bed tertiary academic hospital in Cape Town. An electronic questionnaire explored clinicians' experience of the June 2024 ransomware cyberattack, while laboratory test volumes before and after the incident were compared using routine lab data. | Tygerberg Hospital and the National Health Laboratory Service (NHLS) pathology laboratory, serving approximately 3.6 million people. Survey participants included 58 healthcare professionals (medical officers, registrars, consultants and fellows) involved in patient care during the ransomware attack. | On June 22, 2024, the NHLS suffered a ransomware attack, which resulted in the inability to utilise information technology (IT)-related systems, including the laboratory information system (LIS) (InterSystems TrakCare® Lab Enterprise, Cambridge, Massachusetts, United States). Consequently, many automated processes reverted to manual operations, while new and historic patient laboratory results were inaccessible. This change led to delays in the turnaround time for patient results and made it challenging for clinicians to access historic results and view new results timeously | Clincians reported disruptions to their daily workload and patient care, with 84% experiencing increased stress, 78% describing delayed diagnoses, 73% reporting delays in treatment decisions, 65% describing delayed patient referrals and 62% identifying emergency surgery delays. | [https://pmc.ncbi.nlm.](https://pmc.ncbi.nlm.nih.gov/articles/PMC13088463/) [nih.](https://pmc.ncbi.nlm.nih.gov/articles/PMC13088463/) [gov/articles/PMC130](https://pmc.ncbi.nlm.nih.gov/articles/PMC13088463/) [88463/](https://pmc.ncbi.nlm.nih.gov/articles/PMC13088463/) |
| 53  54  55 | McCarthy et al [78]  Faul et al [67]  Frisch et al [65] | 2026  2022  2022 | 2021  2021  2020 | Ireland  Ireland  USA | Retrospective multicentre observational study using propspectively collected data from the Irish Hip Fracture Database (IHFD) to compare national hip fracture care before and after the 2021 HSE cyberattack. Data from all participating hospitals was analysed across pre and post-cyberattack periods.  A descriptive single-centre case study describing the operational response of St Luke's Radiation Oncology Network after the May 2021 HSE ransomware attack. This paper provides a narrative account of the service disruption, recovery and contingency planning across the three-centre radiation oncology network.  A descriptive single centre case study describing quality assurance, error reduction, billing, compliance and recovery process within an academic pathology lab during prolonged cyberattack-related downtime. The report is part of a four part series documenting the operational response of the medical centre following the ransomware event. | 16 Irish hospitals participating in the Irish Hip Fracture Database. The study included 3784 patients admitted with hip fractures during 2021, representing 99% national case coverage.  St Luke's Radiation Oncology Network, comprising three radiation oncology centres in Dublin with 14 linear accelerators, treating over 5000 new patients annually. At the time of the attack, 304 patients were undergoing radiotherapy, 17 we due to commence treatment and 201 were in treatment planning.  University of Vermont Medical Center (UVMMC) pathology and clinical laboratories within the University of Vermont Health Network, serving five hospitals across Vermon and New york. The study focuses on lab staff, pathologies and the patients who requiired pathology and diagnostic services during the cyberattack. | On 14th May 2021 the HSE of Ireland was subject to a ransomware attack resulting in a national disruption to the IT infrastructure utilised by the health service. This resulted in a major impact on the delivery of patient care with loss of function of the software used for radiology, laboratory results, communications, electronic patient records and many other aspects of the health service. In response to the cyberattack, many services reverted back to paper based systems and radiology images had to be reviewed on monitors at the time of images being recorded due being unable to upload images to the PACS (Picture Archiving and Communication System).  On May 14, 2021, the Health Service Executive (HSE) of Ireland experienced a major ransomware cyberattack. The HSE initially took down all of its information technology systems to protect its core systems. All Internet connections within the HSE were unavailable from 7 am for approximetely three weeks which had a major effect on the radiation oncology service nationally within the public service. St. Luke's Radiation Oncology Network (SLRON) is a complex, 3-center radiation oncology service, and it is the largest in the country; with 14 linear accelerators, it is one of the largest radiation centers in Europe.  This large academic hospital was the victim of a multi-institution, systemwide cyberattack that has been reported as 1 of the 5 most significant cyberattacks in the US health care system in 2020. The University of Vermont Medical Center’s (UVMMC’s) electronic health record (EHR) system, Epic (Epic Systems), remained completely locked throughout the downtime. Third-party applications were also isolated from all network connections, including department-specific modules and systems required for the functioning of pharmacy, scheduling, radiology picture archival and communication system, billing and coding, and payroll. This restriction to network connectivity affected not only UVMMC but also the other nodes serving 5 health network hospitals throughout Vermont and New York. The shutdown ultimately lasted more than 25 days, with some subsystem outages lasting more than 40 days. | The cyberattack significantly reduced compliance with several Irish Hip Fracture Standards. Surgery within 48 hours decreased from 75.3% to 69.5%, timely orthopaedic ward admission declined (27.7% to 24.4%) and hospital acquired pressure ulcers increased (2.1% to 3.3%).  Radiotherapy services were temporarily suspended, resulting in treatment interruptions and delays while services were progressively restored over a two-week period. Patients receiving urgent, paediatric and palliative radiotherapy were prioritised, treatment schedules were modified, certain patients were transferred to private centres and staff work extended hours.  Interaoperative pathology assessments fell due to reduced surgical volume. Extensive staff time was neeed to retrospectively reconcile lab records and billing. Manual reporting increased pathology report amendement rates from 1.6% to 4.2% during the downtime period. | [https://www.](https://www.sciencedirect.com/science/article/pii/S1479666X26000375) [sciencedirect.](https://www.sciencedirect.com/science/article/pii/S1479666X26000375) [com/science/article/pi](https://www.sciencedirect.com/science/article/pii/S1479666X26000375) [i/S1479666X2600037](https://www.sciencedirect.com/science/article/pii/S1479666X26000375) [5](https://www.sciencedirect.com/science/article/pii/S1479666X26000375)  [https://doi.org/10.](https://doi.org/10.1016/j.adro.2022.100993) [1016/j.adro.](https://doi.org/10.1016/j.adro.2022.100993)  [2022.100993](https://doi.org/10.1016/j.adro.2022.100993)  [https://academic.oup.](https://academic.oup.com/ajcp/article/158/1/18/6533598) [com/ajcp/article/158/1](https://academic.oup.com/ajcp/article/158/1/18/6533598)  [/18/6533598](https://academic.oup.com/ajcp/article/158/1/18/6533598) |
| 56  57 | Al Haddad et al [63]  O’Shea et al [69] | 2025  2022 | 2023  2021 | Lebanon  Ireland | A single-center retrospective case study of a cyberattack affecting the clinical laboratory at NDS-US, a university hospital in Lebanon. This study is retrospective and descriptive in nature, focusing on the operational resilience and crisis management aspects of the event. Primary data sources included internal incident reports and logs, spanning the chronology of system outages and restorations, records of testst processed manually and communications detailing interim procedures. Minutes from emergency management meetings and lab staff debriefings were also reviewed.  Technical methods study evaluating an in-house Python-based radiotherapy decision-support tool, designed to compensate for treatment gaps that occured due to a cyberattack. The software was developed and evaluated using high-priority radiotherapy patients whose treatment was interrupted during the May 2021 Ireland Cyberattack. | NDS-US is a tertiary care teaching hospital with a fully computerized hospital information system, including an advanced LIS integrated with automated analyzers. The cyberattack affected all laboratory sections, including clinical chemistry, haematology, microbiology and the blood bank.  University Hospital Galway Department of Medical Physics and Clinical Engineering. 5 High Priority radiotherapy patients experiencing treatment interuptions of 12-13 days following the cyberattack, were included as clinical case studies to evaluate the software. | In 2023, the hospital was targeted by a ransomware cyberattack that abruptly shut down all IT systems. The laboratory, which had been operating as a paperless environment, experienced an immediate halt to electronic orders and reporting. All sections of the lab were affected, including chemistry, hematology, microbiology, and the blood bank. This case study examines the laboratory’s response and recovery from the moment of attack through the subsequent weeks of service restoration.  The May 2021 ransomware attack on the Irish Health Service Executive resulted in loss of electronic patient data and record-and-verify systems, preventing delivery of radiotherapy for up to 12 days. The prolonged interruption prompted development of a software tool to evaluate radiotherapy treatment compensation strategies accounting for tumour repopulation and organ-at-risk dose. | Laboratory testing capacity fell to 40-50% of normal activity during the initial downtime, with elective testing reduced by around 80% while emergency and ICU investigations were prioritised. Manual workflows increased workload and turnaround times. No patient deaths or serious adverse events were directly attributed to the cyberattack, but several near-miss events were identified and used to strengthen emergency preparedness and recovery protocols.  Unschedueled interruptions to radiotherapy treatments lead to decreased tumour control, such that rapid cell repopulation in the tumour may occur due to the absent radiation dose. For oncology patients, this can lead to the recurrence and deterioration of their disease. | [https://pubmed.ncbi.](https://pubmed.ncbi.nlm.nih.gov/40770901/) [nlm.nih.](https://pubmed.ncbi.nlm.nih.gov/40770901/) [gov/40770901/](https://pubmed.ncbi.nlm.nih.gov/40770901/)  [https://pubmed.ncbi.](https://pubmed.ncbi.nlm.nih.gov/35856482/) [nlm.nih.](https://pubmed.ncbi.nlm.nih.gov/35856482/) [gov/35856482/](https://pubmed.ncbi.nlm.nih.gov/35856482/) |

| **Article Number** | **Reference** | **Publication Date** | **Cyber Attack Date** | **Country** | **Study Design and Setting** | **Organisation / Population Affected** | **Attack Characteristics and Systems Disrupted** | **Impact on patients and clinical operations** | **URL** |
| --- | --- | --- | --- | --- | --- | --- | --- | --- | --- |
| 58 | Rajput et al [100] | 2025 | 2024 | UK | A commentary discussing the impact of cyberattacks on NHS Primary Care, predominantly drawing on published evidence, NHS England reportos and the 2024 Synnovis Ransomware attack to highlight gaps in reporting and the need for greater cyberresilience across primary care services. | Primary care services across south-east London, including approximately 194 general practices affected by the Synnovis ransomware attack through disruption of outsourced pathology services. The commentary also discusses wider NHS primary care services affected by the 2017 WannaCry attack. | On June 3, 2024, Synnovis, a partnership between SYNLAB (Europe’s largest provider of medical testing and diagnostics) employed by Guy’s and St Thomas’ NHS Foundation Trust and King’s College Hospital was hit by a ransomware cyberattack demanding US$50 million. The disruption also propagated to the 194 general practice surgeries in southeast London using the Synnovis platform. The attack caused a complete lockdown of Synnovis’s systems, limiting access to pathology services and severely disrupting patient diagnostics, blood transfusion processing, and result reporting. | The synnovis cyberattack caused a complete lockdown of Synnovis’s systems, limiting access to pathology services and severely disrupting patient diagnostics, blood transfusion processing, and result reporting. Final data from NHS England revealed that the disruption led to the cancellation of 10 152 outpatient appointments and 1710 elective procedures being delayed in secondary care at Guy’s and St Thomas’ NHS Foundation Trust and King’s College Hospital. Resolution to near-normal levels was achieved 16 weeks after the initial shutdown. | [https://pubmed.ncbi.](https://pubmed.ncbi.nlm.nih.gov/40414783/) [nlm.nih.](https://pubmed.ncbi.nlm.nih.gov/40414783/) [gov/40414783/](https://pubmed.ncbi.nlm.nih.gov/40414783/) |
| 59 | Fabrice et al [70] | 2025 | 2021 | France | A single-centre descriptive and qualitative case study from Dax General Hospital. The analysis was conducted based on internal data, professional interviews, and a detailed chronology of events from January to October 2021. The study focused on three areas: supply chain, pharmacotechnics, and sterilization. | Dax General Hospital comprising 839 inpatient beds and 149 places across six sites. Affected services included medication and medical device procurement and distribution, outpatient dispensing, chemotherapy preperation, sterilisation, surgery, radiotherapy, clinical services and adminstrative departments. | In February 2021, Dax General Hospital was the target of a major cyberattack that led to the complete shutdown of its Information Technology systems. The Ryuk ransomware compromised approximately 85% of the hospitals 150 servers and rendered all backups inaccessible. Internal and external telephony, email, fax, patient records, electronic prescribing, pharmacy stock and financial management systems, chemotherapy software, sterilisation supervision and administrative applications became unavailable. | The attack caused extensive data loss and prolonged organisational, logistical, financial and psychosocial disruption. Medication databases, supplier records, ward stock lists, prescriptions, treatment protocols and historical data had to be reconstructed manually. Most chemotherapy sessions were initially postponed, cancelled or transferred, with selected treatments maintained through manual double checks, and Excel-based tool and temporary outsourcing. In the hospital sterilisation system, the loss of electronic traceability prevented staff from linking instrument sets to valiated cycles; sterilisation activity was suspended and surgical procedures were cancelled. | [https://www.](https://www.sciencedirect.com/science/article/abs/pii/S2772953225006446) [sciencedirect.](https://www.sciencedirect.com/science/article/abs/pii/S2772953225006446) [com/science/article/a](https://www.sciencedirect.com/science/article/abs/pii/S2772953225006446) [bs/pii/S27729532250](https://www.sciencedirect.com/science/article/abs/pii/S2772953225006446) [06446](https://www.sciencedirect.com/science/article/abs/pii/S2772953225006446) |

Reference list:

11. van Boven LS, Kusters RWJ, Tin D, et al. Hacking acute care: a qualitative study on the health care impacts of ransomware attacks against hospitals. Ann Emerg Med. Jan 2024;83(1):46-56. [doi: ] [Medline: 37318433]

13. Abbou B, Kessel B, Ben Natan M, et al. When all computers shut down: the clinical impact of a major cyber-attack on a general hospital. Front Digit Health. 2024;6:1321485. [doi: ] [Medline: 38433989]

45. Oliver M, Pearce A, Stillwaugh L, Leszczynski K. The impact of a cyberattack at a radiation oncology department: immediate response and future preparedness. Adv Radiat Oncol. 2022;7(5):100896. [doi: ] [Medline: 36148381]

46. Filipec O, Plasil D. The case of the benešov hospital hit by ryuk ransomware, and lessons learned. Obrana a strategie, University of Defence; 2021. [doi: ]

47. Pontier N, Orre M, Martin M. Cyberattack at Dax hospital: presentation of the facts, consequences and feedback. Cancer Radiother. Oct 2022;26(6-7):938-940. [doi: ] [Medline: 36028420]

48. Keogh RJ, Harvey H, Brady C, et al. Dealing with digital paralysis: surviving a cyberattack in a National Cancer center. J Cancer Policy. Mar 2024;39:100466. [doi: ] [Medline: 38176467]

49. Hoffman TW, Baker JF. Navigating our way through a hospital ransomware attack: ethical considerations in delivering acute orthopaedic care. J Med Ethics. Feb 2023;49(2):121-124. [doi: ] [Medline: 35197299]

50. Cassim S, Chapanduka ZC. Cyberattack on the National Health Laboratory Service of South Africa - implications, response and recommendations. S Afr Med J. Nov 29, 2024;114(12):e2549. [doi: ] [Medline: 41378543]

51. Ades S, Herrera DA, Lahey T, et al. Cancer care in the wake of a cyberattack: how to prepare and what to expect. JCO Oncol Pract. Jan 2022;18(1):23-34. [doi: ] [Medline: 34339260]

52. Looi JC, Allison S, Bastiampillai T, et al. Cybersecurity lessons from the Vastaamo psychotherapy data breach for psychiatrists and other mental healthcare providers. Australas Psychiatry. Feb 2025;33(1):106-110. [doi: ]

53. Ghafur S, Kristensen S, Honeyford K, Martin G, Darzi A, Aylin P. A retrospective impact analysis of the WannaCry cyberattack on the NHS. NPJ Digit Med. 2019;2(1):98. [doi: ] [Medline: 31602404]

54. Kelly WH, Narvaez JRF, Hu J, et al. Triumph over adversity: unlocking optimal trauma outcomes during healthcare ransomware attacks. Injury. Dec 2023;54(12):111046. [doi: ] [Medline: 37826882]

55. Pham TT, Loo TM, Malhotra A, et al. Ransomware cyberattack associated with cardiac arrest incidence and outcomes at Untargeted, Adjacent Hospitals. Crit Care Explor. Apr 2024;6(4):e1079. [doi: ] [Medline: 38605720]

56. Neprash HT, McGlave CC, Rydberg K, Henning-Smith C. What happens to rural hospitals during a ransomware attack? Evidence from Medicare data. J Rural Health. Sep 2024;40(4):728-737. [doi: ] [Medline: 38494590]

57. Nelson CJ, Soisson ET, Li PC, et al. Impact of and response to cyberattacks in radiation oncology. Adv Radiat Oncol. 2022;7(5):100897. [doi: ] [Medline: 36148379]

58. Lippi G, Ferrari A. Lessons learnt in medical laboratories during a disruptive cyber-attack. J Lab Precis Med. 2024;9:18-18. [doi: ]

59. Dameff C, Tully J, Chan TC, et al. Ransomware attack associated with disruptions at adjacent emergency departments in the US. JAMA Netw Open. May 1, 2023;6(5):e2312270. [doi: ] [Medline: 37155166]

60. Abouk R, Powell D. Ransomware attacks, ED visits and inpatient admissions in targeted and nearby hospitals. JAMA. Jun 25, 2024;331(24):2129-2131. [doi: ] [Medline: 38809568]

61. Feeley A, Lee M, Crowley M, et al. Under viral attack: an orthopaedic response to challenges faced by regional referral centres during a national cyber-attack. Surgeon. Oct 2022;20(5):334-338. [doi: ] [Medline: 34782238]

62. Neprash H, McGlave C, Nikpay S. Hacked to pieces? The effects of ransomware attacks on hospitals and patients. Am Econ J Econ Policy. Feb 1, 2026;18(1):256-281. [doi: ]

63. Al Haddad C, Boutros T, Finianos P, Germanos M. When code crashes the lab: operational resilience in clinical laboratories amid a cyberattack - a case study from a university hospital. Ann Biol Clin (Paris). Aug 26, 2025;83(4):414-424. [doi: ] [Medline: 40770901]

64. Iacobucci G. NHS makes urgent appeal for blood donations after cyberattack on London hospitals. BMJ. 2024:q1277. [doi: ]

65. Frisch NK, Gibson PC, Stowman AM, et al. Anatomy of a cyberattack: part 4: quality assurance and error reduction, billing and compliance, transition to uptime. Am J Clin Pathol. Jul 1, 2022;158(1):18-26. [doi: ] [Medline: 35188946]

66. Choudhuri B, Prakash J, Pal B, Veenith T. Cyber-resilient intensive care unit: ransomware is a patient-safety crisis-a resource-stratified approach for India. Indian J Crit Care Med. 2026;30(5):363-367. [doi: ] [Medline: 42415897]

67. Faul C, Robinson J, Carey J, et al. Effect of the cyberattack targeting the Irish health system in May 2021 on radiation treatment at St. Luke’s Radiation Oncology Network. Adv Radiat Oncol. 2022;7(5):100993. [doi: ] [Medline: 36148375]

68. Moore G, Khurshid Z, McDonnell T, Rogers L, Healy O. A resilient workforce: patient safety and the workforce response to a cyber-attack on the ICT systems of the national health service in Ireland. BMC Health Serv Res. Oct 17, 2023;23(1):1112. [doi: ] [Medline: 37848947]

69. O’Shea K, Coleman L, Fahy L, Kleefeld C, Foley MJ, Moore M. Compensation for radiotherapy treatment interruptions due to a cyberattack: an isoeffective DVH‐based dose compensation decision tool. J Applied Clin Med Phys. Sep 2022;23(9):e13716. URL: <https://aapm.onlinelibrary.wiley.com/toc/15269914/23/9> [Accessed 2026-08-24] [doi: ]

70. VidalF, Merlet M, Rucheton A, et al. Retour d’expérience sur une cyberattaque au sein d’un hôpital général: impacts et résilience d’une Pharmacie à Usage Intérieur PUI et de son unité de stérilisation. Le Pharmacien Clin. 2026;61(3):258-274. [doi: ]

71. Harrison AS, Sullivan P, Kubli A, et al. How to respond to a ransomware attack? One radiation oncology department’s response to a cyber-attack on their record and verify system. Pract Radiat Oncol. 2022;12(2):170-174. [doi: ] [Medline: 34644601]

72. Perry H, Tsai EM, Perusse K, Herschorn SD, Watson EJ. Breast imaging during a cyberattack and global pandemic: what we did to pick up the pieces. Semin Ultrasound CT MR. Feb 2023;44(1):18-22. [doi: ] [Medline: 36792269]

73. Stowman AM, Cacciatore LS, Cortright V, et al. Anatomy of a cyberattack: part 3: coordination in crisis, development of an incident command team, and resident education during downtime. Am J Clin Pathol. Jun 7, 2022;157(6):814-822. [doi: ] [Medline: 35188562]

74. Harvey H, Carroll H, Murphy V, et al. The impact of a national cyberattack affecting clinical trials: the cancer trials Ireland experience. JCO Clin Cancer Inform. Apr 2023;7:e2200149. [doi: ] [Medline: 37053539]

75. Devi S. Cyber-attacks on health-care systems. Lancet Oncol. Apr 2023;24(4):e148. [doi: ] [Medline: 36934730]

76. Gabbay-Benziv R, Ben-Natan M, Roguin A, et al. When the lights go down in the delivery room: lessons from a ransomware attack. Int J Gynaecol Obstet. Aug 2023;162(2):562-568. [doi: ] [Medline: 36740900]

77. Zhao JY, Kessler EG, Yu J, et al. Impact of trauma hospital ransomware attack on surgical residency training. J Surg Res. Dec 2018;232:389-397. [doi: ] [Medline: 30463746]

78. McCarthy CJ, Hickey P, O’Daly BJ, McGoldrick NP, Quinlan JF. The effect of a national healthcare cyberattack on the Irish hip fracture standards. Surgeon. Aug 2026;24(4):241-245. [doi: ] [Medline: 42091386]

79. Davids A, Fataar A, Zama A, et al. [Clinicians’ perspectives on the impact of a ransomware attack on a chemical pathology laboratory at a tertiary hospital in South Africa. EJIFCC. Mar 2026;37(2):268-280. [Medline: 42006506]](Clinicians%E2%80%99%20perspectives%20on%20the%20impact%20of%20a%20ransomware%20attack%20on%20a%20chemical%20pathology%20laboratory%20at%20a%20tertiary%20hospital%20in%20South%20Africa.%20EJIFCC.%20Mar%202026;37(2):268-280.%20%5BMedline:%2042006506%5D)

80. Goodwin A, Wilburn C, Wojewoda C, et al. Anatomy of a cyberattack: part 2: managing a clinical pathology laboratory during 25 days of downtime. Am J Clin Pathol. May 4, 2022;157(5):653-663. [doi: ] [Medline: 35188951]

81. Stowman AM, Frisch N, Gibson PC, et al. Anatomy of a cyberattack: part 1: managing an anatomic pathology laboratory during 25 days of downtime. Am J Clin Pathol. Apr 1, 2022;157(4):510-517. [doi: ] [Medline: 35188549]

82. Klatt MK. Case study analysis: cybersecurity breach at metropolitan health systems. In: In Multisector Insights in Healthcare, Social Sciences, Society, and Technology. IGI Global Scientific Publishing; 2024:115-135. [doi: ]

83. Haase KK, Whitworth MM, Yalamanchili K. Clinicians’ experiences and reflections from a health system cyberattack. J Am Coll Clin Pharm. Jun 2021;4(6):738-742. URL: <https://accpjournals.onlinelibrary.wiley.com/toc/25749870/4/6> [doi: ]

84. Fink J, Gresko J. Consequences of the change healthcare cyberattack continue. Pharm Times. 2024. URL: <https://www.pharmacytimes.com/view/consequences-of-the-change-healthcare-cyberattack-continue> [Accessed 2026-08-25]

85. Powell J, Monahan R, Slevin B, O’Hara M, O’Connell NH, Dunne CP. Coping with contagions: maintaining infection prevention and control in an Irish tertiary hospital during COVID and a debilitating nationwide ransomware virus in May 2021. J Hosp Infect. Aug 2021;114:180-181. [doi: ] [Medline: 34052282]

86. Santalo O, Perez G, Lorich C, et al. Defining key pharmacist and technician roles in response to a hospital downtime or cyberattack. J Am Pharm Assoc (2003). 2022;62(5):1518-1523. [doi: ] [Medline: 35466072]

87. Gourd E. Increase in health-care cyberattacks affecting patients with cancer. Lancet Oncol. Sep 2021;22(9):1215. [doi: ] [Medline: 34332657]

88. Zhao JY, Kessler EG, Guo WA. Interprofessional communication goes up when the electronic health record goes down. J Surg Educ. 2019;76(2):512-518. [doi: ] [Medline: 30253982]

89. Leonard F, O’Reilly H, Blackburn C, et al. Learnings from a national cyberattack digital disaster during the SARS-CoV-2 pandemic in a pediatric emergency medicine department. Disaster Med Public Health Prep. Jun 26, 2023;17:e419. [doi: ] [Medline: 37357951]

90. Strong H, Pickles A, Hartery A. Lessons learned from a cyberattack on the healthcare system of newfoundland and labrador: a radiology perspective. Can Assoc Radiol J. Aug 2022;73(3):601-602. [doi: ] [Medline: 35199607]

91. Aljaidi M, Alsarhan A, Samara G, et al. NHS wannacry ransomware attack: technical explanation of the vulnerability, exploitation, and countermeasures. Presented at: 2022 International Engineering Conference on Electrical, Energy, and Artificial Intelligence (EICEEAI); Nov 29 to Dec 1, 2022:1-6; Zarqa, Jordan. [doi: ]

92. Chen PH, Bodak R, Gandhi NS. Ransomware recovery and imaging operations: lessons learned and planning considerations. J Digit Imaging. Jun 2021;34(3):731-740. [doi: ] [Medline: 34159418]

93. Duffy C, Murray C, Boran G, Srinivasan R, Kane A, Leonard A. Survey of laboratory medicine’s national response to the HSE cyberattack in the Republic of Ireland. Ir J Med Sci. Apr 2024;193(2):889-896. [doi: ] [Medline: 37737914]

94. Stowman AM, Kalof AN. Surviving a cyberattack in anatomic pathology: disaster response and creation of an incident command system. AJSP Rev Rep. 2022;27(4):171-176. [doi: ]

95. Tarka M, Blankstein M, Schottel P. The crippling effects of a cyberattack at an academic level 1 trauma center: an orthopedic perspective. Injury. Apr 2023;54(4):1095-1101. [doi: ] [Medline: 36801172]

96. Slattery FJ, Farrell E, Lacy PD. The effect of the 2021 Irish cyber-attack on otolaryngology outpatient non-attendance at a model 4 hospital in the post-COVID era. Cureus. Jul 2022;14(7):e26944. [doi: ] [Medline: 35989756]

97. Russell SP, Fahey E, Curtin M, Rowley S, Kenny P, Cashman J. The Irish national orthopaedic register under cyberattack: what happened, and what were the consequences? Clin Orthop Relat Res. Sep 1, 2023;481(9):1763-1768. [doi: ] [Medline: 37036406]

98. Mashinchi MI, Acton T, Datta PM. When healthcare becomes sick: recovering from ransomware. J Inf Technol Teach Cases. May 2026;16(1):92-101. [doi: ]

99. Chen D, Chou SY, Peng XD. Impacts of hospital data breach on healthcare quality. Health Serv Res. Jun 2025;60(3):e14439. [doi: ] [Medline: 39789912]

100. Rajput K, Darzi A, Ghafur S. Overlooked and under-reported: the impact of cyberattacks on primary care in the UK National Health Service. Lancet Digit Health. Jul 2025;7(7):100879. [doi: ] [Medline: 40414783]
