# Supplementary material for: Unveiling Patient-Level Harms and At-Risk Clinical Groups During Hospital Cyberattacks: Systematic Review of Global Case Studies and Social Media Data
Source: J Med Internet Res. 2026 Sep 28;28:e88257. doi: 10.2196/88257 (PMC13622069; doi:10.2196/88257)
Supplement: Supplementary file 5 [file jmir-v28-e88257-s004.docx]

## Appendix 6 - Insights from social media posts regarding cyberattack impacts across wider healthcare infrastructure, outside the hospital and in the community

| **Category of Cyberattack-induced issue** | **Identified Risks and Patient Harms from Social Media Analysis** |
| --- | --- |
| **Affected Healthcare Services Outside of the Hospital**  Patients and staff members reported cyberattack impacts outside of the hospital, spanning impact on “healthcare at home” networks, prehospital medicine and 911 services, community pharmacies, primary care facilities | **Impact on GP Services & Primary Care**  “Just went to the GP all systems down. Just the backlog of work this will create for the NHS means it won't be over tomorrow. Took *** 10 days to recover from there IT failure just last week they got back up. Ironically they were one of the first down again today”.  **Impact on Emergency Services and 911 Phone Lines**  “I work as a paramedic and all our systems are down including the 911 phone line. [Comment] Geez. Where is this, roughly? [Comment] Not OP, but the 911 system in *** *** (which is a single system for the whole state) was able to see people were calling but not answer. However they were able to call people back, so not a total disaster. They switched to a legacy back up and apparently that's working fine”.  **Impact on Healthcare At Home**  “In true NHS form, my local trust has been subject to a cyber attack and seem to be making no progress in fixing the issue. They use ***Net to provide sub-cut infliximab to me. I have ran out and due my next dose on Thursday. I am very concerned as I already had a feeling I was slowly failing the current meds - I suffer from fistulising crohns so the symptoms are sudden and severe when I'm not in remission. I've raised my concern with the IBD team and they're being really blasé about the whole situation. 'Don't worry, we'll just sort your prescription when the computer works again' level of blasé.”  **Impact on Outpatient Therapy Appointments**  “I just ran into this article about Change Healthcare being threatened again in another cyberattack. Has anyone seen this? [URL Removed] [Comment] Patient here-must be true bc I make my virtual therapist appointments through *** and they've been telling me their system is updating and will be back within 1/2 hour, for a week. What a mess.” |
| **Infrastructural Challenges**  Staff and patients reported issues relating to moving around the hospital, accessing wards and contacting clinicians in emergencies. Staff also lost essential documentation on patient locations (ward/bed number) and the “take list” itself which contained the details of the status and location of sick patients in the hospital. | **Concerns around failure of ward CCTV**  **“**Am a nurse in a *** pysch facility... I'm wondering if I'm safe at work... no conclusive word on whether or not the camera systems are operating or accessible. Risk management can't turn their computers on just like the rest of us. Never thought I'd be so stressed about the lack of eye in the sky action. Been very careful not to discuss the situation in front of patients. It's wracking my nerves for sure”.  **Issues with door access and moving around hospital**  **“**Yes, had it happen at my hospital. Some of the doors wouldn’t even open. Our main supply room was one we couldn’t get into. We couldn’t even print hard copies of the MARs at first. Phones down. It was 5 days and functionality came back in a trickle. What a nightmare. Hope you’re back up soon.”  **Issues with paging systems and communication networks**  “I got an email from my hospital. What happened to you guys? [Comment] Entire computer system down. Phones and pagers spotty too.”  **Problems with air conditioning and patient call alarms**  “Work at *** in the ***, here were the highlights: (1) staff called Pensacola to have the a/c temp lowered (because ofc a site 2 hours away should control our a/c), staff was told NO, because the request wasn’t emailed (obv we can’t email) (2) call lights are connected to the “system” so they weren’t working, so every pt got a little old timey front desk bell, hilarity ensued (3) docs losing their shit because they suddenly had to hand write SHIT TON of orders for things like transfer of CABG to the ICU, more hilarity ensues Thank god we had a cold pasta lunch for nurses week. [Comment] The bells are maybe better than whistles, which were presented as another option for a call light replacement.  **Loss of the patient “Take List”**  “*** Hospital is a mess. All our discharge summaries for the weekend have had to be re-written, blood tests are inaccessible (and not all being processed), and almost all our radiology is written off. Our medical take list and referral system are all gone, as well as the computerised list of sick patients needing review. Oh and switchboard and the bleeping system are also broken. Also, it turns out the 'C' in CT stands for computerised /s- so they're all pretty much rinsed”.  **Patient locations being wrongly documented and lost in hospital**  **“**So many critical values missed, many mis-entered values (thankfully nothing crazy/significant on this front from what I’ve seen verifying work or what I’ve heard from others), I heard from my friend that works pharmacy at my hospital that meds were wildly mismanaged, patients being lost/marked as being incorrect rooms and/or floors, and so many receiving problems we can’t fix to correctly bill these patients”. |
