# Supplementary material for: Unveiling Patient-Level Harms and At-Risk Clinical Groups During Hospital Cyberattacks: Systematic Review of Global Case Studies and Social Media Data
Source: J Med Internet Res. 2026 Sep 28;28:e88257. doi: 10.2196/88257 (PMC13622069; doi:10.2196/88257)
Supplement: Supplementary file 7 [file jmir-v28-e88257-s007.docx]

**Appendix 2 - PRISMA-S Checklist**

| **Section/topic** | **#** | **Checklist item** | **Location(s) Reported, with justification added for omitted items** |
| --- | --- | --- | --- |
| **INFORMATION SOURCES AND METHODS** | | | |
| **Database name** | 1 | Name each individual database searched, stating the platform for each. | Methods - Part 1: Systematic Review - Search Strategy and Information Sources |
| **Multi-database searching** | 2 | If databases were searched simultaneously on a single platform, state the name of the platform, listing all of the databases searched. | Methods - Part 1: Systematic Review - Search Strategy and Information Sources |
| **Study registries** | 3 | List any study registries searched. | N/A - this is justified in the methods section, explaining that study registries were not searched as cyberattack incident case studies are not registered prospectively as clinical trials. |
| **Online resources and browsing** | 4 | Describe any online or print source purposefully searched or browsed (e.g., tables of contents, print conference proceedings, web sites), and how this was done. | N/A - this is justified and discussed in the Limitations section, as grey literature was not pursued. |
| **Citation searching** | 5 | Indicate whether cited references or citing references were examined, and describe any methods used for locating cited/citing references (e.g., browsing reference lists, using a citation index, setting up email alerts for references citing included studies). | N/A - No forward or backward citation searching was performed. This is acknowledged as a limitation in the Discussion section. |
| **Contacts** | 6 | Indicate whether additional studies or data were sought by contacting authors, experts, manufacturers, or others. | N/A - No author or expert contacts were made to identify additional studies. Acknowledged as a limitation in the Discussion section. |
| **Other methods** | 7 | Describe any additional information sources or search methods used. | Methods Part 2 - Social media data mining of Reddit subreddits was conducted as a supplementary evidence stream to identify patient and clinician narratives of cyberattack-induced harms not captured in academic literature. Full methodology described in Methods, Part 2. |
| **SEARCH STRATEGIES** | | | |
| **Full search strategies** | 8 | Include the search strategies for each database and information source, copied and pasted exactly as run. | Methods Part 1 and Appendix 1 - Complete executable search strategies for all five databases are provided in Appendix 1 and described in the Methods. |
| **Limits and restrictions** | 9 | Specify that no limits were used, or describe any limits or restrictions applied to a search (e.g., date or time period, language, study design) and provide justification for their use. | Methods - Part 1: Systematic Review - Search Strategy and Information Sources |
| **Search filters** | 10 | Indicate whether published search filters were used (as originally designed or modified), and if so, cite the filter(s) used. | N/A - no published methodological search filters were applied. This review targets observational case studies and mixed-methods designs for which validated RCT or other study design filters would be inappropriate and would exclude the majority of eligible evidence, consistent with Cochrane guidance that filters should match the study designs sought |
| **Prior work** | 11 | Indicate when search strategies from other literature reviews were adapted or reused for a substantive part or all of the search, citing the previous review(s). | N/A - The search strategy was developed de novo for this review; no published search strategies from prior systematic reviews were adapted or reused. |
| **Updates** | 12 | Report the methods used to update the search(es) (e.g., rerunning searches, email alerts). | Methods - Part 1: Systematic Review - Search Strategy and Information Sources and Appendix 1. |
| **Dates of searches** | 13 | For each search strategy, provide the date when the last search occurred. | Methods - Part 1: Systematic Review - Search Strategy and Information Sources and Appendix 1. |
| **PEER REVIEW** | | | |
| **Peer review** | 14 | Describe any search peer review process. | N/A  - The search strategy was developed in full accordance with Cochrane guidance and MECIR standards. Absence of formal peer review acknowledged as a limitation in the Discussion, Limitations section. |
| **MANAGING RECORDS** | | | |
| **Total Records** | 15 | Document the total number of records identified from each database and other information sources. | Methods, Appendix 1 (Search strategies) and Manuscript PRISMA Flowchart |
| **Deduplication** | 16 | Describe the processes and any software used to deduplicate records from multiple database searches and other information sources. | Methods, Appendix 1 (Search strategies) and Manuscript PRISMA Flowchart |
|  |  |  |  |
| PRISMA-S: An Extension to the PRISMA Statement for Reporting Literature Searches in Systematic Reviews | | |  |
| Rethlefsen ML, Kirtley S, Waffenschmidt S, Ayala AP, Moher D, Page MJ, Koffel JB, PRISMA-S Group. | | |  |
| Last updated February 27, 2020. | |  |  |
