# Supplementary material for: Unveiling Patient-Level Harms and At-Risk Clinical Groups During Hospital Cyberattacks: Systematic Review of Global Case Studies and Social Media Data
Source: J Med Internet Res. 2026 Sep 28;28:e88257. doi: 10.2196/88257 (PMC13622069; doi:10.2196/88257)
Supplement: Supplementary file 8 [file jmir-v28-e88257-s008.docx]

**Appendix 4 - SWiM Reporting Checklist: Synthesis Without Meta-analysis (SWiM) items: SWiM is intended to complement and be used as an extension to PRISMA, taken from the original SWiM methods article by Campbell et al (2019) [1].**

| **SWiM reporting item** | **Item description** | **Page in manuscript where item is reported** |
| --- | --- | --- |
| **Methods** | | |
| 1 Grouping studies for synthesis | 1a) Provide a description of, and rationale for, the groups used in the synthesis (eg, groupings of populations, interventions, outcomes, study design) | Page 4 [Data Collection, Selection and Synthesis Process - During data extraction, each identified patient-level harm was allocated to the most relevant clinical specialty by clinical members of the research term, with harms affecting multiple specialities assigned accordingly. Grouping by clinical speciality was chosen as it directly reflects our objective of identifying vulnerable patient groups and is clinically actionable for preparedness planning]. |
|  | 1b) Detail and provide rationale for any changes made subsequent to the protocol in the groups used in the synthesis | Page 4 [Data Collection, Selection and Synthesis Process - groupings determined inductively from the evidence rather than pre-specified; harms affecting multiple specialties assigned to all relevant groups; acknowledged as a limitation]. |
| 2 Describe the standardised metric and transformation methods used | Describe the standardised metric for each outcome. Explain why the metric(s) was chosen and describe any methods used to transform the intervention effects, as reported in the study, to the standardised metric, citing any methodological guidance consulted | Page 5 [Data Collection, Selection and Synthesis Process - this review catalogues patient-level harms rather than estimating intervention effects; descriptive frequency counts per specialty and data source used; no mathematical transformation applied. The synthesis addresses whether evidence of harm exists within each clinical domain rather than its magnitude]. |
| 3 Describe the synthesis methods | Describe and justify the methods used to synthesise the effects for each outcome when it was not possible to undertake a meta-analysis of effect estimates | Page 5 [Data Collection, Selection and Synthesis Process - descriptive frequency counts of harms per clinical specialty and per data source (academic vs social media) used as synthesis method; justified on grounds that included studies report structurally incompatible outcome types precluding meta-analysis]. |
| 4 Criteria used to prioritise results for summary and synthesis | Where applicable, provide the criteria used, with supporting justification, to select the particular studies, or a particular study, for the main synthesis or to draw conclusions from the synthesis (eg, based on study design, risk of bias assessments, directness in relation to the review question) | Page 4 [Eligibility Criteria and Data Collection, and Selection and Synthesis Process - all 49 included studies contributed equally to the synthesis; no studies prioritised or excluded on grounds of study design or risk of bias]. |
| 5 Investigation of heterogeneity in reported effects | State the method(s) used to examine heterogeneity in reported effects when it was not possible to undertake a meta-analysis of effect estimates and its extensions to investigate heterogeneity | Page 5 [Data Collection, Selection and Synthesis Process - heterogeneity examined informally by comparing distribution of harms across academic and social media sources, and across countries and attack types]. |
| 6 Certainty of evidence | Describe the methods used to assess the certainty of the synthesis findings | Page 6 [Study Quality and Bias Assessment - formal GRADE assessment not performed given structural heterogeneity of outcomes and descriptive nature of synthesis; study-level quality assessed using MMAT and interpreted qualitatively when assessing strength of evidence per specialty group]. |
| 7 Data presentation methods | Describe the graphical and tabular methods used to present the effects (eg, tables, forest plots, harvest plots) | Page 10 [Data Collection, Selection and Synthesis Process & Appendix 4 and 6 - findings presented in Figure 1 and Appendix 6 ordered by clinical specialty; included studies presented in Appendix 4 ordered by country with key characteristics including study design, setting, attack type, and affected population]. |
|  | Specify key study characteristics (eg, study design, risk of bias) used to order the studies, in the text and any tables or graphs, clearly referencing the studies included | Page 10 [Data Collection, Selection and Synthesis Process & Appendix 4 and 6 - findings presented in Figure 1 and Appendix 6 ordered by clinical specialty; included studies presented in Appendix 2 ordered by country with key characteristics including study design, setting, attack type, and affected population]. |
| **Results** | | |
| 8 Reporting results | For each comparison and outcome, provide a description of the synthesised findings and the certainty of the findings. Describe the result in language that is consistent with the question the synthesis addresses, and indicate which studies contribute to the synthesis | Page 10 [Results, “Cyberattack sensitive conditions and At-Risk Patient Groups” - findings reported by clinical specialty with contributing studies cited throughout; academic and social media sources distinguished to allow assessment of each evidence stream's contribution; findings described in terms of presence and nature of harm per specialty rather than magnitude]. |
| **Discussion** | | |
| 9 Limitations of the synthesis | Report the limitations of the synthesis methods used and/or the groupings used in the synthesis and how these affect the conclusions that can be drawn in relation to the original review question | Page 21 [Discussion, Limitations - limitations addressed include post-hoc nature of specialty groupings; inability to estimate harm magnitude, probability, or clinical significance; absence of formal certainty assessment; social media data unverified and anonymous serving as supplementary rather than confirmatory evidence; underreporting bias during cyberattack events acknowledged]. |

PRISMA=Preferred Reporting Items for Systematic Reviews and Meta-Analyses

**References**

[1] Campbell, M., McKenzie, J. E., Sowden, A., Katikireddi, S. V., Brennan, S. E., Ellis, S., ... & Thomson, H. (2020). Synthesis without meta-analysis (SWiM) in systematic reviews: reporting guideline. bmj, 368.
