# Supplementary material for: Correction: Unveiling Patient-Level Harms and At-Risk Clinical Groups During Hospital Cyberattacks: Systematic Review of Global Case Studies and Social Media Data
Source: J Med Internet Res. 2026 Nov 4;28:e114623. doi: 10.2196/114623 (PMC13733283; doi:10.2196/114623)
Supplement: Supplementary file 1 [file jmir-v28-e114623-s001.docx]

## Appendix 4 - Extracts from the full CIPHER Database with examples of identified patient-level harms from the academic literature (column 1) and the social media data (column 2), categorised by clinical domains (numbered as Sections for reference throughout the text. Quotes are provided from the authors of the academic paper alongside the respective reference, or from the social media post alongside the respective subreddit. Social media data posts are deidentified (references to usernames are removed) and any personal details or reference to specific providers/vendors are replaced with asterix, quotes are numbered (e.g. Q1.1) for reference throughout the text.

| **Patient Groups and Clinical Conditions** | **Identified Risks - From Systematic Review** | **Identified Risks - From Social Media Analysis (Subreddit name with quote)** |
| --- | --- | --- |
| **Section 1: Acute Care and Patients with Critical Conditions** | **Loss of Intranet Protocols in Emergency Care:** Emergency care protocols were unavailable during the attacks and had to be retrieved from the internet or borrowed from other hospitals. Hospital staff often felt lost without the availability of computers and described it to be crippling [Van Boven et al, 2024].  **Critical lab tests:** 21% [of laboratories] had no access to a phone line within the laboratory, which impacted the communication of critical results to clinical staff, with 33% of respondents reporting difficulties in contacting requesting clinicians [Duffy et al, 2024].  **Impact on blood transfusions and acute care:** The NHS has made an urgent appeal for O blood type donors in response to the cyberattack that forced some of the largest hospitals in London to cancel operations and divert patients to other trusts [Lacobucci, 2024].  **Increase in Stroke cases at hospitals neighbouring the victim hospital:** We saw increases in stroke code alerts, stroke diagnoses, and acute treatments with tPA and endovascular treatments during the cyberattack and recovery.  Several of the hospitals targeted by the ransomware attack are stroke centers, necessitating the transport of these high-acuity patients to a reduced number of functioning stroke centers in the region [Dameff et al, 2023]. | **Q1.1 - r/hacking** - **Cath Labs:** “All of our cathlabs except for one are down and our sister hospital has no cathlabs. They are transferring patients to our facility. No anesthesia services. It took out the outpatient offices as well. I'm here trying to figure out how to find out what's going on with my patients. Haven't done a paper H&P in 10 years.”  **Q1.2 -r/nursing - Delays in Cardiac Enzymes:** “More people need to be talking about this honestly. I work at a level one trauma in the ER. It’s absolute madness. I can’t even describe the stuff that’s going on. All I know is people are going to suffer and die if we continue like this. We were on diversion for like 5 days but it’s all about money so we had to come off. I think today was possibly one of the worst shifts I had in my entire life. Couldn’t hold tears back and couldn’t even talk to the doctor asking me questions at the end of my shift or I was gonna start sobbing if I spoke out loud lol just nodding my head I was so stressed out and anxious. Had a trop come back 4 hours later at 1850 and it was 11. If that doesn’t just prove how dangerous this is then I don’t know anymore”  **Q1.3 - r/nursing -Issues with Imaging and Contrast Allergy:** “When x-ray doesn't get electronic orders or know who's ready for imaging, they may get hundreds of phone calls from all the nurses around the hospital. When CT doesn't have the patient chart in front of them, they can't double check for a contrast allergy. When lab has to print out all their results on paper and fax or tube them to the recipient, results can get lost and trends can go unnoticed.”  **Q1.4 - r/medicine - Delays for Brain Bleeds and Blood Clots:** “Let me tell you what I am a nurse in the emergency room at a XX XX hospital and it is a freaking disaster we are doing three times the work it's taking double the time not to mention critical patients that need to wait two to three times as long to get stat results for brain bleeds or blood clots. It is so unsafe the staff is just so busy and overworked and exhausted from patients being rude to them. So many documents are going to go missing, everything is so disorganized it's unbelievable the amount of records that will go missing during this time is unbelievable. I was looking to change positions anyway due to a denial of a raise and and now I'm going to work four times as hard give crappy patient care and receive the same...no thanks”  **Q1.5 r/hacking - Overflow of Patients:** “We’ve had overflow from the main building in our ER for weeks. Treating people in the lobby..”  **Q1.6 - r/medicine - Prescribing Issues:** “My hospital lost EMR access one night for like 3 hours due to a network outage and it was a disaster. Couldn’t pull meds. Couldn’t print lab labels so everything had to have backup slips and walked down instead of tubed down. Couldn’t see lab results or imaging. We were just shy of resorting to using leeches again”  **Q1.7 - r/hacking - EMS on Diversion:** “We are down in Florida. It’s a hot mess in the ER today. EMS diversion on cardiac patients because the cath lab is down. But of course all other EMS is accepted because of course we can’t lose any money over this although we are working with minimal staff and it’s clearly not safe for patients…”  **Q1.8 - r/nursing - Loss of Phone Contact: “**I had a similar thing happen at my old hospital. Saw a dude die and couldn’t get ahold of doctors because no one had the attendings fucking number. Not once mentioned in the news that we had been hacked; and there was zero support”  **Q 1.9 - r/nursing - Issues with access control systems and doors:** “Yes, had it happen at my hospital. Some of the doors wouldn’t even open. Our main supply room was one we couldn’t get into. We couldn’t even print hard copies of the MARs at first. Phones down. It was 5 days and functionality came back in a trickle. What a nightmare”.  **Q 1.10 - r/medicare - Risks for Asthma Patients:** “The recent Medicare ransomware attack has left me unable to get my disabled husband's asthma medications for his nebulizer. In 2021 my husband had cardiac arrest due to asthma attack and putting him in a coma, he is now living back home, but severely disabled,he depends on me for everything, has trach, feeding tube, diapers, bed bound etc.” |
| **Section 2: Blood Transfusion Services and Haematology Patients** | **Transfusions & Loss of Blood Type Data:** Abbou et al described the risks associated with loss of blood type information, and the potential mislabelling of patient blood type compromising patient safety [Abbhou et al, 2024].  **Decreased Blood Transfusions:** Multiple sites reported decreasing blood transfusions to a minimum, negatively impacting patients with hematological conditions and those with planned surgeries (e.g. Obstetric patients awaiting C-Sections) [Moore et al, 2023; Abbhou et al, 2024]. | **Q1.11 - r/blooddonors - Impact on Blood Transfusion Services:** “There was recently a big ransomware cyberattack on the NHS that knocked out a load of systems including Synnovis pathology labs. A lot of blood testing had to be halted while the systems were restored…I’ve been to blood donation sessions recently where they were literally phoning up non-type-O donors on the day to cancel them so they could fit more type-O donors in.”  **Q1.12 - r/blooddonors - Impact on Blood Donations:** “If you have a donation scheduled with the American Red Cross today, be aware that computer outages are preventing donations from being taken. This is not at every single location, but it does seem to be widespread.” |
| **Section 3: Impact on Cancer patients and Oncology services** | **Surgical Oncology & Biopsy Results:** Researchers reported failures in definitive surgeries, due to loss of biopsy results, precluding confirmation that cancer was fully removed: “*Preoperative planning and image guided localization procedures in breast imaging were stopped for three weeks and severely curtailed for over a month due to lack of access to diagnostic workup and diagnostic mammography workstations”* [Keogh et al, 2024].  **Laboratory issues for oncology patients:** Lack of access to laboratory results posed clear risks to the patients undergoing major oncological surgery including safe anesthesia. Access to biopsy results that diagnosed cancer (e.g., core biopsies and endoscopic biopsies) were initially unavailable precluding definitive surgery. For other cases, non-availability of diagnostic imaging prevented surgical planning [Ireland Conti Ransomware, Keogh et al, 2024]  **Interrupted treatments for oncology patients:** Cancellation of chemotherapy and radiotherapy treatments, due to loss of prescriptions and treatment protocols. “Most critically the patients who were  on treatment having radiotherapy there was absolutely no way of continuing their treatment” [Moore et al, 2023].  **Disruption to Linear Accelerators precluding radiation therapy:** St. Luke's Radiation Oncology Network (SLRON) is a complex, 3-center radiation oncology service in Dublin, Ireland. With 14 linear accelerators, it is the largest center in the country and one of the largest radiation centers in Europe… ARIA also serves as the record and verification system for the 10 Varian linear accelerators in the network…. The IT servers that host ARIA are all located in the center in St. James. All the servers were disconnected and, as a result, all the linear accelerators and planning terminals were shut down across SLRON. Wi-Fi, email access (internal or external), and web-based phone and video systems were all unavailable [Faul et al, 2022].  **Blood Transfusions:** Inability to provide blood products impact patients awaiting transfusions, especially those with hematological cancers. | **Q1.13 - r/MedicalPhysics - Impact on Radiation Therapy:** "Do you have any experience with cyber attacks affecting your TPS or R&V system? I think there have been several cases of hospitals that were not able to treat patients for many days because a cyberattack affected seriously the R&V system. I suppose in these situations it may be justified to do some simple treatments (emergencies or electrons) using the linac as a stand-alone machine, programming the fields manually and filling in a paper chart, but I was wondering how this could be done in the *** because they don't let you program fields manually unless you enter service mode (with full access to all the service parameters), or you can have the linac in clinical mode and send the fields from the icomCAT software instead of from ***, although creating the fields in these software is a little tricky and it is designed only for acceptance tests (you are warned that it is "not for clinical use"), so some doubts about liability come to my mind..."  **Q1.14 - r/coloncancer - Impact on Cancer Services:** “I am mad! My *** was to start his Oral Chemo & Radiation treatment today. It is on hold until further notice due to the Cyberattack on *** (his medical provider) computer system”  **Q1.15 - r/BrainCancer - Impact on Brain Tumour Resection:** “My *** care for grade 4 glioblastoma was delayed due to the ransomware. We had a craniotomy brain resection done at the end of March & he was doing great. Walking again within 4 days, driving again within 12. Then came the part where we scheduled treatment. We were told we would be contacted, & we weren't. We started calling them & didn't get an answer & nobody returned voicemails”  **Q1.16 - r/Migraine - Disruptions for Cancer Patients:** “My *** is a specialty pharmacist working with primarily cancer patients. This has been hell for them since Wednesday.”  **Q1.17 - r/lungcancer - Missed Cancer Recurrence:** “Our province experienced a cyber attack on hospitals which led to a delay in her follow ups and they missed her cancer returning by 3 months. It has then metastasized and she passed within 3 days of finding it.”  **Q1.18 - r/GriefSupport - Patient death after missed screening: “**My *** beat small cell lung cancer in 2022. Due to a cyberattack in hospitals in Ontario, she missed her most recent cancer screening by 3 months and her family doctor basically ignored her symptoms. She went to the hospital last Thursday for shortness of breath and they discovered the cancer had returned in both lungs, her pericardium of the heart and her liver. She was supposed to start treatment on Tuesday but died in palliative care on Sunday”. |
| **Section 4: Palliative Care & Clinical Trial Patients** | **Impact on the Dying:** The shutdown of IT systems led to “difficulties in accessing necessary information to help treat and care for symptomatic dying patients in our community at a critical time for them” [Keogh et al, 2024]  **Clinical Trials:** Harvey and colleagues described that the ransomware attack led to an 85% fall in referrals and 55% fall in recruitment for trials for clinical trials, significantly affecting patients' access to trial therapies [Harvey et al, 2023]. Harvey and colleagues dive into the specific impact on clinical trial patients, identifying the negative sequelae of (1) Gaps in treatment, (2) Patients unable to communicate with clinical trials teams leading to increased anxiety, (3) Patients were at increased risk of error due to staff fatigue, (4) Patients were more likely to get lost in the system, (4) Health records posted illegally online affecting confidentiality, (5) Limited access to diagnostics and (6) Protocol deviations compromising eligibility [Harvey et al, 2023]. | **Q1.19 r/GriefSupport:** “Due to a cyberattack in hospitals in ***, she missed her most recent cancer screening by 3 months and her family doctor basically ignored her symptoms. She went to the hospital last Thursday for shortness of breath and they discovered the cancer had returned in both lungs, her pericardium of the heart and her liver. She was supposed to start treatment on Tuesday but died in palliative care on Sunday.”  **Q1.20 r/nursing:** “Some docs were trying to give us verbal orders to put in ourselves like usual and we’re like bro do you see what’s happening right now. The straw broke the camel’s fucking back and we’re all suffering now. Patients are being diverted to other hospitals because we can’t operate like this (not to mention our hospital just had a basement flood this week). I’m scared for my patients and my license. It took me 6 hours to get my pt transitioned to comfort care and get morphine orders. I can’t follow up with docs now because communication is so clogged up”. |
| **Section 5: General Medicine and Chronic Conditions** | **Loss of treatment protocols for medical inpatients:** Hospitals are transitioning to digital advisory tools for determining patient treatments, such as the use of heparin advisory tools based on blood test results. As staff become dependent on these systems, this creates a vulnerability where loss of these systems may prevent treatment of related conditions - the authors give the example of heparin administration and vancomycin protocols [Haase et al, 2021].  **Hepatic and Renal Impairment:** For patients with renal and hepatic impairment, delays in laboratory processing posed challenges for drug and opioid prescribing [Keogh et al, 2024].  **Nephrology and General Surgery Department see heightened delays:** Delays in obtaining laboratory results were widely reported, affecting diagnostic timelines (78% agreed, n=45) and decisions regarding initiating or discontinuing patient treatment (73%, n=42). The nephrology department reported the highest percentage of delayed diagnoses (100% agreed), with general surgery and medical interns reporting similarly high levels (87.5% and 83.3% agreed, respectively). [Davids et al, 2026].  **Loss of specific reference ranges:**  (i) As well as all the appropriate patient-specific identifiers, an electronic patient report contains, among other things, age-appropriate reference ranges, units of measurement, alert flags and interpretative comments. Production of paper reports during this time was a manual process requiring a high level of scientific input to ensure the quality of results was maintained [Duffy et al].  (ii)While 52% (n=30) agreed that communication of critical results was timely, 48% (n=28) reported issues with missing results, and 39% (n=23) noted difficulties interpreting results due to the lack of reference intervals, automatic calculations, or interpretive comments [Davids et al, 2026]. | **Q1.21 - r/Asthma - Worsening of symptoms:** “I have been feeling awful like I have bronchitis and my chest feels like it’s bubbling and gurgling. I was really looking forward to getting started with Nucala after it finally got approved. But my hospital had a huge cyber/ransomware attack and they cannot open their computers. So who knows when I will be able to get back in. I’m so fucking depressed and feel so fucking miserable. Breathing burns and I just give up.”  **Q1.22 - r/rheumatoid - Lost prescription access:** “Yeah I had trouble with a refill script for Enbrel going through and I’m down to my last two shots.”  **Q1.23 - r/Ozempic - Lost prescription access:** “Anyone else having issues getting their ozempic with a third party coupon from Pharmacy?”.  **Q1.24 - r/CrohnsDisease - Lost prescription access:** “In true NHS form, my local trust has been subject to a cyber attack and seem to be making no progress in fixing the issue. They use Home*** *** to provide sub-cut infliximab to me. I have ran out and due my next dose on Thursday. I am very concerned as I already had a feeling I was slowly failing the current meds - I suffer from fistulising crohns so the symptoms are sudden and severe when I'm not in remission”  **Q1.25 - r/medicare - Issues obtaining Dexcom Sensors:** “I get a new batch of Dexcom sensors each month which are considered DME and covered by Medicare Part B. A couple of days back I submitted a request for my next batch and was told by my *** pharmacy that the Part B system for DME was hacked and is down.”  **Q1.26 - r/migraine - Escalated treatments required:** “I called today CVS and was told the same thing, I am 3 weeks overdue my dr had to give me a shot in my hip yesterday because I had a migraine so bad [Comment] I was just at CVS and they said some of the savings cards are starting to go through, but none for Emgality yet.”  **Q 1.27 - r/HealthInsurance - Hospitalisation due to worsened symptoms:** “I couldn’t get my Spiriva filled and was off it for 5 days due to the cyber attack and was hospitalized for 3 days for an asthma exacerbation and missed work the second week after the attack occurred. Our ability to receive eScripts those first two weeks was down so we had to do hard copies, verbals, or faxes (most providers went with faxes). “ |
| **Section 6: Surgical Patients & Orthopaedics** | **Missed Injuries:** Missed injuries (e.g. fractures) due to unavailable imaging, and inability to check reports [Zhoa et al].  **Cancelled Operating Room Services and Loss of Vital Software:**  (i) The closure of operating rooms was reported by numerous academic sources and Feeley et al reported barriers for orthopaedic patients when digital templating software was lost, preventing selection of implant size and position amongst orthopedic patients [Feeley et al, 2021; Zhao et al].  (ii) The PACS (imaging) and bed management system were rendered inaccessible during the cyberattack. The inability to efficiently identify and allocate available orthopaedic beds is likely to have contributed to delays in ward admission, as clinical teams reverted to manual processes, as well as the difficulty in identifying hip fractures due to the impacted radiological software [McCarthy et al, 2026].  **Increased rate in hospital-acquired pressure ulcers:** The rate of hospital-acquired pressure ulcers (IHFS 3) showed a numerical increase from 2.1% pre-attack to 3.1% post-attack in the matched analysis, though this did not reach statistical significance (p = 0.109). In the full-year analysis the worsening reached significance (2.1% to 3.3%, p = 0.024), suggesting the burden on pressure ulcer prevention may have accumulated beyond the immediate post-attack period [McCarthy et al, 2026]. | **1.28 -** r**/ChronicPain:** My *** had major surgery and was released from the hospital yesterday. The pharmacy was closed so the hospital gave her pain medicine right before she left. Her doctor said he sent in the scripts (2 for pain) last night. I watched him do it on the computer. 9:30 AM I call the pharmacy to see if the medicine is ready and the pharmacist tells me there’s no scripts there for MIL. According to the pharmacist the computer systems have been hacked (on Wednesday) and they haven’t been able to get many things that were sent in electronically.  **Q1.29 - Loss of Pyxis / Medication Delivery systems**: “This happened to my hospital last year, we were down like 2-3 days. I was working in the GI Lab at the time and had to cancel all elective procedures because it affected our Pyxis and anesthesia machines. The amount of people who prepped for a colonoscopy just for it to be canceled the day of their procedures” |
| **Section 7: Obstetric Patients & Women’s Health** | **Obstetric patients:** Participants in acute and maternity services were completely reliant on electronic methods and were therefore more severely impacted [Moore et al, 2023] | **Q 1.30 - r/birthcontrol - Impact on accessing birth control:** “I just don’t get it and I’m on day 2 with no birth control and I’m freaking out a lot because I have endometriosis and PCOS and I skip the placebo pills so I don’t have period.”  **Q 1.31 - r/pregnant - Impact on foetal monitoring scans:** “On top of my own crazy, I should be going to my 35 week appt with a growth scan on Monday (which would make me feel 1000x better) but it hasn’t been scheduled yet because the hospital where my doctor office is located (and where I’ll give birth!) has been dealing with a freaking ransomware attack since the beginning of the month”.  **Q 1.32: r/miscarriage - Impact on care for miscarriages:** “I went to the ER with intense sharp pains, heavy bleeding, light-headedness, dizziness, and heavy clotting during my 5 week pregnancy…. I was able to squeeze into a nearby OB for **[date]. The hospital I got ultrasound scans from is under a cyber attack so I can't even obtain my ultrasound images or medical records.”  **Q1.33 - r/babybumps - Impact on Labour Care:** “I’m not trying to alarm or scare you, but my hospital was under a ransomware attack during my labor. It ended up causing them to not give me my asthma medicine for 72 hours until I flipped shit on them. They also came in and manually took my blood pressure every 15 mins. It sucked. I wasn’t given much information about my recovery.”  **Q1.34: r/pregnant - Impact on pregnant mothers due to give birth:** “the title explains it all. my doctors office is the victim of a cyber attack and i am a very clueless 38w 5d along ftm. with the cybersecurity breach they cancelled my appointment last week, this week and wont be seeing me next week presumably. what the HELL do i do? my last appointment i was closed, 50% effaced and having contractions every 10 mins. as of yesterday i've lost my mucus plug and feel contractions a little closer together and some of them are painful. i cant reach my doctor, i don't want to keep showing up to l&d for no reason.”  **Q1.35 - r/babybumps - Impact on contracting obstetric team**: “I'm 22 weeks and at an *** for care as well. I cant login to my *** to message my doctor or see my appointment times which is inconvenient. I keep thinking what if something happens to me and baby and I need to go get checked out sometime soon?”  **Q1.36 - r/nursing - Infant Death and Foetal Monitoring:** “Happened to my hospital almost two years ago for I think almost two weeks. The L&D nurses had to just listen for the baby’s heart rate by sitting near the doors because the central monitoring strips were down. Somehow, the monitor was tracking the mothers heart rate and they didn’t realize it wasn’t the baby’s for a while. The baby was down for so long it had no brain activity after birth and they withdrew care. People will die without modern technology.”  **Q1.37 - r/medicine - Infant Death and Fetal Monitoring**: [In response to Baby Ransomware Death] “Reminds me of when I tried to put someone on telemetry for HyperK after being consulted by the OB unit... which meant they wheeled in a cardiac monitor and put it in the room but only the patient was watching it”  **Q1.38 - r/womenshealth - Impact on Cervical Screening:** “I used to always get my pap results back within a few days. (I get them yearly) Last year it took 11 days and it was abnormal. I had to get a colposcopy. It’s been over 2 weeks this year, and I still don’t have the results back. When I asked the dr’s office, they said things were taking longer because of a cyber attack that happened over 2months ago.”  **Q1.43 - r/hysterectomy - Post-op recovery from hysterectomy:** “For me, I woke up shaking, gave me some meds, stopped. They brought me a sprite zero to sip on and I was fine. Downside is my hospital computer system got hacked so I was in recovery for quite awhile waiting for a room to open up.” |
| **Section 8: Paediatrics & Neonates** | **Newborn Identification and Birth Certificates:** Several hospitals reported issues producing birth certifications and digital authentication of new births, precluding usually follow-up protocols (including safeguarding plans) [Abbhou et al, 2024]. | **Q1.44: r/babybumps - Issues with birth certificates:** “I gave birth at ascension with this cyber attack, the first day it happened and it did not affect my care at all as a patient! I feel bad for the nurses and doctors and all staff having to deal with this though. The only thing it’s affecting right now is the process of them doing the birth certificate and social security card because they have to manually enter everything.”  **Q1.45 - r/babybumps - Issues with birth certificates:** “There were some communication problems between nurses since some things weren't written down. I also left the hospital without the birth certificate info. They told me they won't know when they would be able to process his birth information to obtain his birth certificate.”  **Q1.46 - r/medicine - Critical deterioration in diabetes patients:** “Our group had to transfer critically ill children to neighboring facilities because we couldn’t do basic labs in DKA and other common illnesses.”  **Q1.47 - r/medicine - Children receiving poorer nutrition one wards:** “my *** is in a hospital now being attacked by ransomware and as a consequence he is not getting the right diet (bad for him), not getting his meds on time (not good either), can't get released as billing records not up to date as scanning system not working and many records not accessible. I”  **Q1.48 - r/cybersecurity - Socioeconomic impact of lost coverage:** “This is a tough one for us, as our *** is on medication where our insurance does not cover it, however there is a coupon that drops medication from $1000 to $50. Unfortunately, the coupon is run through the impacted system, and is not processing so we either have to Switch to a new med (not advised) or pay upfront.”  **Q1.49 - r/nursing - Impact on paediatric medications:** “A ransomware attack happened at a children's hospital where I live a couple of years ago and it was awful. My son couldn't even get his records from the outpatient clinics in order to get his specialty meds approved from insurance and he had to go over a month with no meds (22k a month for his meds without insurance) and the nurses were so stressed out and on the verge of tears at my son's appointment and I felt terrible for them.”  **Q1.50 - r/nursing - Impact on neonatal care:** “Had unplanned downtime for the latter half of a shift with a very sick baby that was needing drips and blood transfusions. Written orders, personal number exchanges with docs, running to pharmacy and blood bank… absolute nightmare. A”  **Q1.51 r/nursing - Impact on paediatric intensive/critical care:** “I showed up Sunday to surprise CVICU paper charting. But only like 9 PCU patients and on divert, all cases cancelled. Yesterday and today I was called off and only 5 or fewer patients now. They are trying to DC everyone. HR got sent home yesterday. I was told Blacksuit hacker group got into something, encrypted it with randomware, and then *** elected to shut down everything else for containment. Including for a while cafeteria payment system lol.” |
| **Section 9: Psychiatry, Neuropsychiatry and Neurology Patients** | **Increase in psychiatric presentations in week 2 as patients begin to re-present locally: “**We demonstrated a significantly sharp drop in the number of admissions to the pediatric and psychiatric emergency wards in the week of the attack, accompanied by a consistent increase in these admissions after the first 7 days. This was regardless of interventions made by the hospital administration and the system recovery status. We assume this patient population, opted to return to the nearest hospital in any case.” [Abbhou et al] | **Q1.52 - r/migraine - Lost prescription access:** “Has anyone had issues filling their qulipta with the cyber attack?..  **Q1.53 - r/migraine - Lost prescription access:** “My pharmacy got cyber-attacked, all medications are cash only for now: Yep, a US hospital network pharmacy got hit with a cyber attack and they have been unable to bill ANY insurance for medications since Wednesday.”  **Q1.54 - r/migraine - Lost prescription access:** “Anyone else having issues filling prescriptions because of the cyber attack? *** said there is a nationwide system outage and my normally free emgality injection with insurance + manufacturers coupon would be $400 and I can’t pay that. In just 6 months I’ve reduced my migraine days from 10-15 to 3 with the injection and hate to not use it.>> I’m having the same problem with my Aimovig. Normally it’s $5 with the coupon but because of the issues it’s over $600 this month. I’m hoping it gets sorted out this week before I need it.  **Q1.55 - r/therapists - Impact on virtual therapy: “**Patient here-must be true bc I make my virtual therapist appointments through *** and they've been telling me their system is updating and will be back within 1/2 hour, for a week. What a mess.”  **Q1.56 - r/adhdwomen - ADHD medications:** “due to my doctor's carelessness in writing prescriptions, I won't have any medication until next week. I am a student. fml.”  **Q1.57 - r/ADHD - Unfilled Prescriptions:** “All of us medicated folk have had so much trouble with shortages and pharmacies unable to fill our prescriptions. There’s currently yet another nationwide situation that will add to our difficulties.”  **Q1.58 - r/ADHD - New Medication Side Effects**: “My doctor just changed me to 20mg of adderall xr after successfully being on 10 mg dyanavel successfully for about 3 months. Insurance wouldn’t cover dyanavel and with that ransomware attack on *** pharmacy nationwide the coupon I was using wouldn’t work. So he switched me to adderall as insurance would cover it. Anyway. I’ve been on adderall for 4 days now and I feel tipsy all the time. I feel like I’m in a fog like you feel with you drink too much, except it’s all the time.”  **Q1.59 - r/migraine - Failure in Savings Cards:** “I’ve been using a Qulipta and a *** savings card at my local pharmacy for 18 months. Last week when I went to refill, the savings card wouldn’t work. The pharmacy stated it was still not working because sure of that Ransomware attack”.  **Q1.60 - r/cybersecurity - Suicide following therapy note breach:** "Hacker jailed for blackmailing therapy patients: At least 1 suicide attributed to an attacker that failed to extort the company and instead turned to the patients whos data he had. It might not happen to you or people you know directly, but it does happen."  **Q1.61 - r/adhdUK - Impact on ADHD patients:** “I use a hospital pharmacy and have been yet again (as if the shortage hasn’t been hell enough) have been unable to get meds for a week—And still don’t have them. So, got written up at work for a total ADHD mistake- leaving a project, half done walking away, forgetting that I was even supposed to finish project and going home at the end of the day, and then my manager found it and confronted me with a write up and a I don’t understand why the hell you just didn’t finish it… And all I could come up with is I have ADHD and I can’t get my medication again… And she has the nerve to say well. This is a good job if you have ADHD. Apparently effing not. I’m so sick of this shit so so so so sick of this shit”  **Q1.62 - r/anxiety - Cyberattack concerns inducing trauma symptoms:** “Having a panic attack had small one earlier and . I went to sleep tryied to ignored but I woke up and got on FB and everyone talking about the nationwide cell phone outage mainly att but did affect other providers too. So far it didn't affect mine but everyone keeps saying it was a cyber attack that either Russia or China are trying to kill our service so they can come attack ect. I know when there outages or events like this ppl like to make theories n conspiracies but it's triggering my severe PTSD and anxiety. Really freaking me out n scared as hell rn.”  **Q1.63 - r/anxiety - Cyberattack news inducing anxiety:** “I’ve been seeing so much about a cyber attack, and an attack on the powergrid and it’s making me so anxious. I don’t have the money to buy a generator and even more so I live in an apartment. I also live 6 hours away from my parents and my phone is the only way we can communicate until our schedules match up to plan a visit. I’m so anxious, I don’t even know what to believe anymore.”  **Q1.64 - r/adhdwomen - Fears of Data Breach:** “I’m starting my diagnosis process, I’ve been calling so many clinics but they no longer diagnose adults, and I found one that I’m going to try on Tuesday. But as I’m doing all my research a question was posed to me. Are there any long-term negative effects that come from being officially diagnosed? Could it become a problem later if it has to go on a sibling’s Family Medical History, or if there’s a data breach and it comes out that I’ve been diagnosed with a mental illness, or if I decide to adopt and it comes up in background checks? Is there anything else I should be cautious about?  **Q1.65 - r/ADHD_partners - Concerns about psychiatric data being breached:** “My *** (DX but unmedicated) was using one of those online therapy programs for about 6 or 7 months, and last night she told me she quit the program. I feel super conflicted about it. We had been talking about her possibly quitting for a couple of weeks, but she just went through with it out of the blue. She had some pretty valid points for why she quit, it wasn't just some spur of the moment thing; we're trying to build up some more savings and that was her biggest monthly expense that came out of her account, her therapist was giving a lot of "try mindfulness meditation" advice which she has been doing for years (thus why she was hoping a therapist would give her new advice, but that didn't work), and she found an article about those therapy programs selling personal data, and one of them (not the one she used but it still made her nervous) having a data breach and people private messages with their therapists being compromised.”  **Q1.66 - r/medicine - Impact on Neurology patients with acute bleeds:** “Let me tell you what I am a nurse in the emergency room at a XX XX hospital and it is a freaking disaster we are doing three times the work it's taking double the time not to mention critical patients that need to wait two to three times as long to get stat results for brain bleeds or blood clots. It |
| **Section 10: Impact on Healthcare Staff and Reports from Clinicians** | **Impact on staff influenced by seniority:** Junior members of staff, including residents, may be more vulnerable to cyberattack effects having trained exclusively on digital systems and less experience returning to paper-based methods (e.g. written prescriptions) [Zhao et al]. | **Q1.67 r/emergencymedicine - Cyberattack causing significant distress for staff:** SOS I cannot anymore. I'm not just talking epic. Literally everything, no computers, no phones, no imaging, doc halo, or ***. Can’t log into computers whatsoever, phones are just dead, can’t have downtime boards, no vocera, no call lights. Even the HVAC system!! We are a level one trauma and stroke center, and of course this happened on a Sunday. We’re running around and giving personal cell phone numbers to all the floors and admin won’t let us go on any type of diversion!!!!!!!!  **Q 1.68 - r/emergencymedicine - Cyberattack causing significant distress for staff:** “Im on night 3 of no imaging reads. No XR, CT, or US. We can order studies, but they can’t be read. Admin’s advice was “aren’t you a doctor? You can’t read a CT scan?”.  **Q1.69 - r/Nurse - Staff report being told not so share/discuss details of cyberattack:** “Did anyone else have their entire *** go down today? None of our staff (physicians, nurses, ancillary staff) could access any notes, imaging, charting, labs, orders.. nothing. Ours never goes out for more than a couple hours, but they're projecting this to last at least another 2 days. Super weird, was wondering if this was just a localized incident or some weird cyber attack BS. We were told to "keep quiet" about it, which always makes me suspicious as hell.”  **Q1.70 - r/hacking - Staff report being told not so share/discuss details of cyberattack:**"*** here were also down, no patient death reported due to this but its bad. Were having a total black out, we were told not to post online at the risk of our jobs. [Comment] Risk your job. Yup sounds like ***. It is the same at our spot in the northeast. [Comment] Same in North Texas!"  **Q1.71 - r/hacking - Staff report being told not so share/discuss details of cyberattack:** As a patient of a *** inpatient hospital, staff was told not to let patients know about the breach.  **Q1.72 -** **r/hacking - Staff report compromised CCTV systems:** Am a nurse in a *** pysch facility... I'm wondering if I'm safe at work... no conclusive word on whether or not the camera systems are operating or accessible. Risk management can't turn their computers on just like the rest of us. Never thought I'd be so stressed about the lack of eye in the sky action. Been very careful not to discuss the situation in front of patients. It's wracking my nerves for sure.  **Q1.73 - r/nursing - Cyberattack causing wider structural effects on air conditioning and patient alarm systems:** “Work at *** in the ***, here were the highlights: (1) staff called *** to have the a/c temp lowered (because ofc a site 2 hours away should control our a/c), staff was told NO, because the request wasn’t emailed (obv we can’t email) (2) call lights are connected to the “system” so they weren’t working, so every pt got a little old timey front desk bell, hilarity ensued (3) docs losing their shit because they suddenly had to hand write SHIT TON of orders for things like transfer of CABG to the ICU, more hilarity ensues Thank god we had a cold pasta lunch for nurses week.  [Comment] The bells are maybe better than whistles, which were presented as another option for a call light replacement."  **Q 1.74** - **r/nursing - Cyberattack causing wider structural effects on air conditioning and patient alarm systems:** Yes, had it happen at my hospital. Some of the doors wouldn’t even open. Our main supply room was one we couldn’t get into. We couldn’t even print hard copies of the MARs at first. Phones down. It was 5 days and functionality came back in a trickle. What a nightmare. Hope you’re back up soon.  **Q1.75- r/nursing - Staff discuss intentions to quit:** “How long are you giving them to get the computers back working before you quit, or will you eventually quit at all? I feel like I’ve been put in an impossible situation, and I know you all probably do too. They’re asking us to override every single medication. I understand there’s no other choice, but it will be the nurses in court and losing their license when something goes wrong with a patient because of this cyberattack. I imagine a judge would say “You knew the conditions were unsafe, and you continued to accept assignments at that hospital. You were still responsible for keeping the patient safe, and failing technology is no excuse for not doing do.” How long are you willing to put your license at risk before you put in your notice? Because for me, I really can’t decisively what the right thing to do is - I only started this job a few months ago.”  **Q1.76: r/nursing - Staff discuss ward based stress and intention to quit:** "I’m a new grad nurse at an *** facility on a tele/medsurg floor approaching the 3 month mark and I’ve been feeling extremely down over this cyberattack. Old nurses are gonna shoot me down for saying this but the paper charting system is absolutely atrocious and in no way was my unit prepared for any of this chaos that’s going on right now. Orders not being received, lab results not being faxed back on time, pharmacy not being prompt with meds, certain pt cares not being done/not documented, papers flying left and right. EVERY department is backed up. I’m supposed to be on my own now that my orientation is supposed to be over but I fear for my license as well as feel unsupported and anxious ass a new grad being thrown into this bullshit and will be telling my manager this tonight before my shift. (I already committed a med error because of paper MARS). There’s no ETA to when this will be fixed and in all honesty, I’m really not trying to stick around and find out when it will be fixed because I was already hating the job to begin with but tolerating it to at least gain the medsurg/tele experience that every new grad needs to build that foundation. Now it’s literally intolerable and I dread every shift to the bone and I’m not even on my own yet. What should I do? Most places probably wouldn’t take a new nurse with only 3 months of bedside exp. I’m at a complete loss right now." |
